# Detection of antibodies against the African parasite *Trypanosoma brucei* using synthetic glycosylphosphatidylinositol oligosaccharide fragments

**DOI:** 10.1007/s10719-025-10186-x

**Published:** 2025-06-24

**Authors:** Maurice Michel, Benoit Stijlemans, Dana Michel, Monika Garg, Andreas Geissner, Peter H. Seeberger, Daniel Varón Silva

**Affiliations:** 1 Biomolecular Systems Department, Max-Planck-Institute of Colloids and Interfaces, Am Mühlenberg 1, 14476 Potsdam, Germany; 2 Department of Biology, Chemistry and Pharmacy, Freie Universität Berlin, Arnimallee 22, 14195 Berlin, Germany; 3 Department of Oncology and Pathology, Science for Life Laboratory, Karolinska Institutet, Tomtebodavägen, Stockholm, 23A, 17121 Sweden; 4 Laboratory of Cellular and Molecular Immunology, Brussels Center for Immunology (BCIM), Vrije Universiteit Brussel, Brussels, 1050 Belgium; 5 Myeloid Cell Immunology Laboratory, VIB Center for Inflammation Research, Brussels, 1050 Belgium; 6 School of Life Sciences FHNW, Institute for Chemistry and Bioanalytics, Muttenz, 4132 Switzerland

**Keywords:** GPI, VSG, Human African Trypanosomiasis, Nagana, carbohydrate antigens, diagnostics

## Abstract

*Trypanosoma brucei* (*T. brucei*) parasites cause two major infectious diseases in Africa: African trypanosomiasis in humans (HAT) and Nagana in animals. Despite the enormous economic and social impact, vaccines and reliable diagnostic measures are still lacking for these diseases. The main obstacle to developing accurate diagnostic methods and an active vaccine is the parasite’s ability for antigenic variation, impairment of B cell maturation, and loss of B cell memory which collectively prevent the development of a long-lasting, effective immune response. The antigenic variation is sustained by random gene switching, segmental gene conversion, and altered glycosylation states of solvent-exposed regions of the corresponding variant surface glycoproteins (VSGs). These glycoproteins use a glycosylphosphatidylinositol (GPI) anchor for attachment to the membrane. GPIs of *T. brucei* have specific branched structures that are further heterogeneously galactosylated. Here, we synthesized a glycan fragment library containing *T. brucei* GPIs’ most prominent structural features and performed an epitope mapping using mice and human sera of infected specimens using glycan microarrays. The studies indicate that in contrast to VSGs, *T. brucei* GPIs are recognized by infection-induced short-lived Immunoglobulin M (IgM) and long-lasting Immunoglobulin G (IgG), suggesting a specific immune response against GPI structures. These findings enable the development of diagnostic tests based on synthetic antigens for the reliable diagnosis of human African trypanosomiasis and Nagana.

**Supplementary Information:**

The online version contains supplementary material available at 10.1007/s10719-025-10186-x.

## Introduction

Subspecies of the African extracellular parasite *T. brucei* cause two infectious diseases in rural areas of Africa, Human African Trypanosomiasis (HAT) and Nagana in animals. Limited diagnosis and treatment, a lack of trained point-of-care personnel, and restricted access to medical facilities have created a beneficial environment for the parasite and its vector, the tsetse fly [1, 2]. A *T. brucei* infection is characterized by a haemolymphatic phase that displays symptoms such as weakness and fever, and a neurologic phase associated with severe anemia, sleep cycle disruption and progressive mental deterioration [3]. The symptoms of the first phase of HAT are not uncommon in sub-Saharan Africa and often leave the infection undiagnosed in animals and humans. Thereby, it can progress to the second stage and becomes lethal if not treated by chemotherapy [4, 5]. 

*T. brucei* is a resilient parasite infecting humans. The parasite’s ability to persist in the face of both the innate and adaptive immune system is achieved by several mechanisms involving the heterogeneity and structural organization of cell surface antigens [6, 7]. 

The outer surface of *T. brucei* is covered by a dense coat of a single phenotype of a variant surface glycoprotein (VSG) attached to the membrane by a glycosylphosphatidylinositol (GPI) anchor [8, 9]. VSGs participate in the complement system inhibition, installation of a diffusion barrier, antibody scavenging, masking of other surface proteins and induce the production of (auto)antibodies [10–14]. Furthermore, *T. brucei* uses antigenic variation by randomly expressing one VSG construct out of several hundred genes in the genome. The possibility of switching the responsible gene between generations increases the probability of immune system evasion over several parasite generations [15–17]. Segmental gene conversion and mosaicism translate to a new, unique phenotype in the solvent-exposed N-terminal and C-terminal domains, respectively [18–23]. Recent reports also showed VSG sequences displaying up to three glycosylation sites, with a unique *O*-glycosylation at the top of the solvent-exposed N-terminus covering the amino acid sequence [21]. 

The heterogeneity of surface antigens hinders their application for developing consistent diagnostic methods and vaccines against *T. brucei* infections. Currently, trypanosomiasis diagnosis is divided into three stages [3, 22–24]. The first stage includes screening for infections by serological tests and analysis of clinical signs, i.e., swollen lymph nodes. The second and third stages involve microscopic confirmation of parasite presence in the blood (infection in phase 1) and the cerebrospinal fluid (infection in phase 2). A commonly used serological test is the card agglutination test for trypanosomiasis (CATT). This test shows improved thermostability, selectivity and specificity. But, it is limited to detecting infections of only a subset of *T. brucei gambiense (Tbg)* strains expressing the VSG LiTaT1.3 variant and does not work for *T. brucei rhodesiense (Tbr)* [25]. Furthermore, the test is not objectively reproducible and is based on fixed *Tbg* parasites, which demand a constant supply of cultivated parasites and trained medical personnel. In addition, determination of the infection phase is essential to select the appropriate therapy and the need to use crossing blood-brain barrier drugs for a phase 2 infection. In recent years, the number of intravenous infusions has been considerably reduced by combining chemotherapeutic drugs for trypanosome infections, thereby enhancing patients’ quality of life [26–28]. 

Current efforts to improve infection diagnosis focus on serological methods and antibody detection. Numerous lateral flow tests based on singular parasite-derived VSGs have recently been developed [29]. A dual-antigen lateral flow test using sVSG117 in combination with a cell lysate protein, rISG65 [30], as immunodiagnostic antigens was reported for detecting trypanosome infections in humans with a reasonable specificity [31]. Considering heterogeneity-based antigenic variation, this study suggests sVSG117 as either a dominant parent gene in segmental gene conversion and mosaicism or as a pre-existing reactive epitope located at the C-terminal domain of VSGs.

**Fig. 1:**
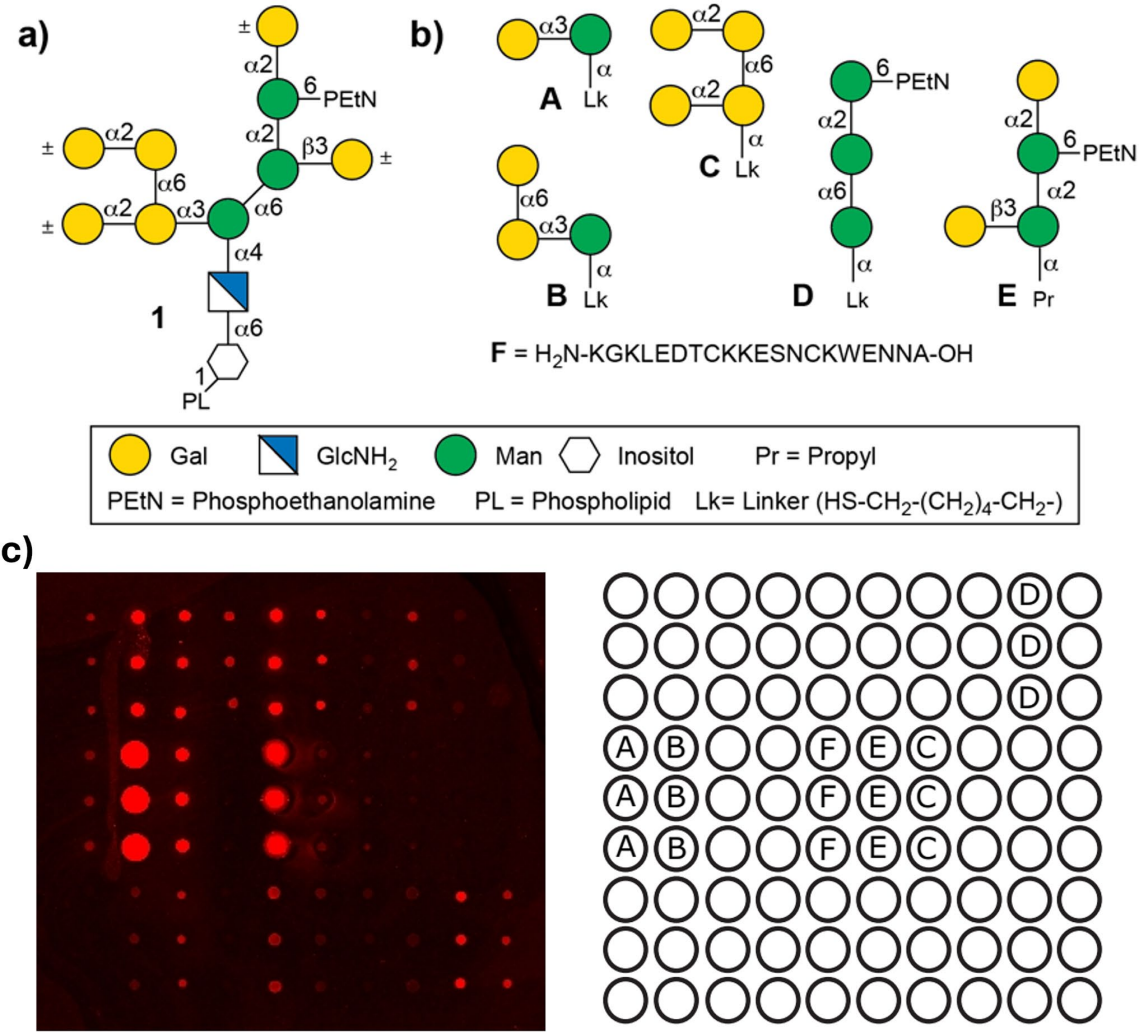
T. brucei GPI derivatives: (**a**) Representation of the *T. brucei* GPI core; (**b**) *T. brucei* GPI fragments containing mannose with different galactosylation patterns **A**-**E** and low variant CTD-peptide of VSG117 (**F**); (**c**) Representative Scan of a microarray indicating the positions of structures **A–F** (left) and Glycan-array printing pattern (right)

In contrast to VSGs, the structure of GPIs only depends on the glycosylation machinery of the cell. The structural variations include the presence or absence of galactoses attached to the conserved GPI pseudopentasaccharide core (Fig. 1a) [32]. We recently described the synthesis of diverse GPI glycolipids using a general convergent strategy and a set of fully orthogonal protecting groups [33]. We expanded this strategy to obtain a series of galactosylated GPI fragments from the GPI of *T. brucei* and its subspecies [34, 35]. The necessary introduction of α-galactoses required additional ester-based protecting groups and adapted synthetic strategies.

Synthetic GPI derivatives have shown potential application in serodiagnosis by determining anti-GPI antibodies in patients infected with *Toxoplasma gondii* and *Plasmodium falciparum* [36–41]. Hence, we hypothesized that the host immune system recognizes, besides the VSG protein core, also the C-terminal region of VSGs and more specifically the galactosylated structures of the trypanosome GPI glycans, inducing specific antibodies that could bind to synthetic structures and be used to detect an infection. The main advantage of investigating chemically synthesized GPI derivatives over VSGs derived from parasitic cultures is the higher accessibility and availability of greater homogeneous amounts. Here, we evaluate a series of synthetic GPI fragments with specific modifications of the natural *T. brucei* GPIs (Structures **A**-**F**, Fig. 1b) to determine HAT infections. We printed the synthetic compounds on glass slides and used the obtained glycan array to detect anti-GPI antibodies in the sera of mice infected with trypanosome parasites. We show that GPI glycan-specific recognition depends on the presence of α-galactose and demonstrate that these structures can be used to detect infection-induced short-term IgM and long-term IgG anti-GPI antibodies. Furthermore, we analyzed sera from *Tbg* and *Tbr* infected patients using the GPI array. We demonstrated for the first time the presence of specific antibodies recognizing synthetic GPI glycans and a peptide present in the VSG-GPI interface.

## Materials and methods

### General synthetic methods

All chemicals were reagent grade, and all solvents were anhydrous, high-purity grade and used as supplied except where noted otherwise. Unless otherwise stated, reactions were performed in oven-dried glassware under an inert argon atmosphere. The reaction molarity was 0.1 molar except where otherwise noted. Reagent-grade thiophene was dried over activated molecular sieves before use. Pyridine was distilled over CaH_2_ before use. Sodium hydride suspension was washed with hexane and THF and stored in an anhydrous environment. Benzyl bromide was passed through activated basic aluminum oxide before use. Before use, molecular sieves were powdered and activated by heating under a high vacuum. Analytical thin layer chromatography (TLC) was performed on Merck silica gel 60 F_254_ plates (0.25 mm). All compounds were visualized by UV irradiation and/or heating the plate after dipping it into a staining solution. The compounds were stained with cerium sulfate-ammonium molybdate (CAM) solution, basic potassium permanganate solution, acidic ninhydrin-acetone solution or a 3-methoxyphenol-sulfuric acid solution. Flash column chromatography was carried out using a forced flow of the indicated solvent on Fluka silica gel 60 (230–400 mesh, for preparative column chromatography).

^1^H, ^13^C and ^31^P-NMR spectra were recorded on a Varian 400 (400 MHz), a Varian 600 (600 MHz), a Bruker 400 (400 MHz) and a Bruker Ascend 400 (400 MHz) spectrometer in CDCl_3_ (7.26 ppm ^1^H, 77.1 ppm ^13^C), D_2_O (4.79 ppm ^1^H), MeOD (4.87 ppm and 3.31 ppm ^1^H, 49.00 ppm ^13^C), acetone-d6 (2.05 ppm and 2.84 ppm ^1^H, 206.26 ppm and 29.84 ppm ^13^C) unless otherwise stated. Coupling constants are reported in Hertz (Hz). Splitting patterns are indicated as s, singlet; d, doublet; t, triplet; q, quartet; br, broad singlet; dd, doublet of doublets; m, multiplet; dt, doublet of triplets; h, sextet for ^1^H NMR data. Signals were assigned using ^1^H-^1^H COSY, ^1^H-^1^H TOCSY, ^1^H^1^H NOESY, ^1^H-^1^H ROESY, ^1^H-^13^ C HSQC, ^1^H-^13^ C HMBC spectra and versions thereof. ESI mass spectral analyses were performed by the MS-service at the Institute for Chemistry and Biochemistry at the Free University of Berlin using a modified MAT 711 spectrometer, the MS-service at the Institute for Chemistry at the University of Potsdam using an ESI-Q-TOF micro spectrometer and a Waters Xevo G2-XS QTof coupled with an Acquity H-class UPLC. Infrared (FTIR) spectra were recorded as thin films on a Perkin Elmer Spectrum 100 FTIR spectrophotometer. Optical rotations were measured with a Schmidt & Haensch UniPol L 1000 at a concentration (c) expressed in g/100 mL. HPLC-supported purifications were conducted using Agilent 1100 and Agilent 1200 systems. Supercritical fluid chromatography was carried out using a Waters Investigator System.

### Ethics statement

All experiments concerning the mice complied with the ECPVA guidelines (CETS n° 123) and were approved by the VUB Ethical Committee (Permit Number: 14-220-29). Human serum samples used for this research were obtained from the WHO HAT Specimen Biobank and stored at the Pasteur Institute. WHO acquired patient consent, and serum samples were collected to develop new diagnostic tests [42]. 

### Patient sera

Patient infection status was determined by applying the CATT test, subsequent parasitological analysis and examination of clinical symptoms of HAT [42]. Serum samples were stored in the WHO HAT Specimen Biobank at − 80 °C, shipped to Potsdam on dry ice, divided into aliquots and stored at − 20 °C.

### Parasites, mice and infections

Clonal pleomorphic *T. brucei* AnTat 1.1E parasites were a kind gift from N. Van Meirvenne (Institute for Tropical Medicine, Belgium) and stored at − 80 °C. Female wild-type (WT) C57Bl/6 mice (7–8 weeks old) were obtained from Janvier and infected with 5 × 10^3^ AnTat1.1E trypanosomes (intraperitoneally (i.p.) in 200 µL HBSS (Hanks’ balanced salt solution, ThermoFisher Scientific)).

### Mice sera

Blood was collected from CO_2_ euthanized non-infected and *T. brucei* infected mice via cardiac puncture, centrifuged (15 min, 10.000xg, 4 °C), and serum was kept at -80 °C.

### Preparation of glycan microarray

The synthetic glycans were dissolved in sodium phosphate buffer (50 mM, pH 8.5 for amine linker compounds) or PBS buffer (pH 7.4 for thiols, including an equimolar amount of TCEP·HCl). The compounds were immobilized in four copies employing a piezoelectric spotting device (S3, Scienion) on maleimide-functionalized slides or epoxy slides (sciCHIPEPOXY, Scienion), in 50% relative humidity at 23 °C. The printed slides were stored for 18 h in a humidified chamber to complete the immobilization reaction. Afterwards, the slides were stored in a cooled environment. Before the experiment, the slides were washed three times with water, and the remaining maleimide or epoxy groups were quenched by incubating the slides in an aqueous solution of 100 mM ethanolamine in sodium phosphate buffer (50 mM, pH 9.01) for 1 h at 25 °C. The slides were rinsed three times with water and dried by centrifugation. Microarrays were blocked with BSA (2.5%, w/v) in PBS for 1 h at room temperature. Blocked slides were washed twice with PBS, centrifuged and incubated with a 1:15 dilution of mouse or human sera in PBS for 1 h. After washing with PBS, microarrays were incubated with goat anti-mouse IgG H + L Alexa 645 (Molecular Probes, 1:400), donkey anti-mouse IgM Alexa 594 (Dianova, 1:200), goat anti-human IgG-Fc Alexa488 (Dianova, 1:400) or goat anti-human IgM Alexa 594 (Molecular Probes, 1:200) in PBS containing 1% BSA for 1 h. The slides were then washed with PBS and double-distilled water, subsequently dried by centrifugation and analyzed using a fluorescence microarray scanner (Genepix^®^ 4300 A, Molecular Devices).

### Data processing and statistical analysis

Data were imported into GenePix Pro, and a mask was superimposed on the fluorescent area, separating it from the background. The difference between fluorescence (inside) and background (outside) was calculated for each position and normalized against the mean of all samples corresponding to the same synthetic structure. The resulting data from duplicate scans were imported into GraphPad Prism, and outliers were removed. ANOVA with multiple comparisons and an initial significance test by Tukey was performed. Then, an unpaired T-test with the following definition of significance was performed: not significant (ns) = *p* > 0,05; * = *p* < 0,05; ** = *p* < 0,01; *** = *p* < 0,001; **** = *p* < 0,0001. Receiver operating characteristic (ROC) curves were generated when *p* < 0,05 existed for a synthetic structure in either murine IgM or IgG scan and for one disease stage for human IgM or IgG infected with *Tbg* and *Tbr*, respectively.

## Results

### Selection of synthetic VSG-GPI fragments

We previously reported the synthesis of a series of *T. brucei* GPI fragments [34]. Using synthetic precursors of these molecules, we obtained a second series of compounds featuring specific GPI modifications and a linker for the immobilization and production of glycan microarrays. We focused on the most prominent structural feature in GPIs from *T. brucei*, which is the presence of α-galactosylation at the C-3 position of Man-I. Interestingly, the GPIs of VSG117, VSG221, and VSG121 variants have been investigated, revealing additional and heterogeneous galactosylation with up to two galactosides along a conserved 1→6 linkage [43]. Covering these structures in our library, we designed the substructures **A-C** to perform a specific GPI epitope mapping. To mimic the oligomannose part in parasitic GPIs, we added a trimannose structure (**D**) that lacks the mammalian phosphorylation of Man-I at the C2 position. Indeed, earlier reports suggested that the absence of this phosphorylation leads to recognition by antibodies generated during an immune response [36, 37]. As a final GPI fragment, the tetrasaccharide **E** [35], bearing two galactose residues, was included to address the heterogeneity of trypanosome VSG221 and VSG121 [34, 43]. A final structure, the peptide (KGKLEDTCKKESNCKWENNA) **F**, was designed to cover the VSG117 CTD that connects the protein to the GPI and is also part of a region with expected low structural diversity [31].

### IgM and IgG antibodies derived from *Trypanosoma brucei*-infected mice specifically recognize synthetic GPI fragments 

**Fig. 2:**
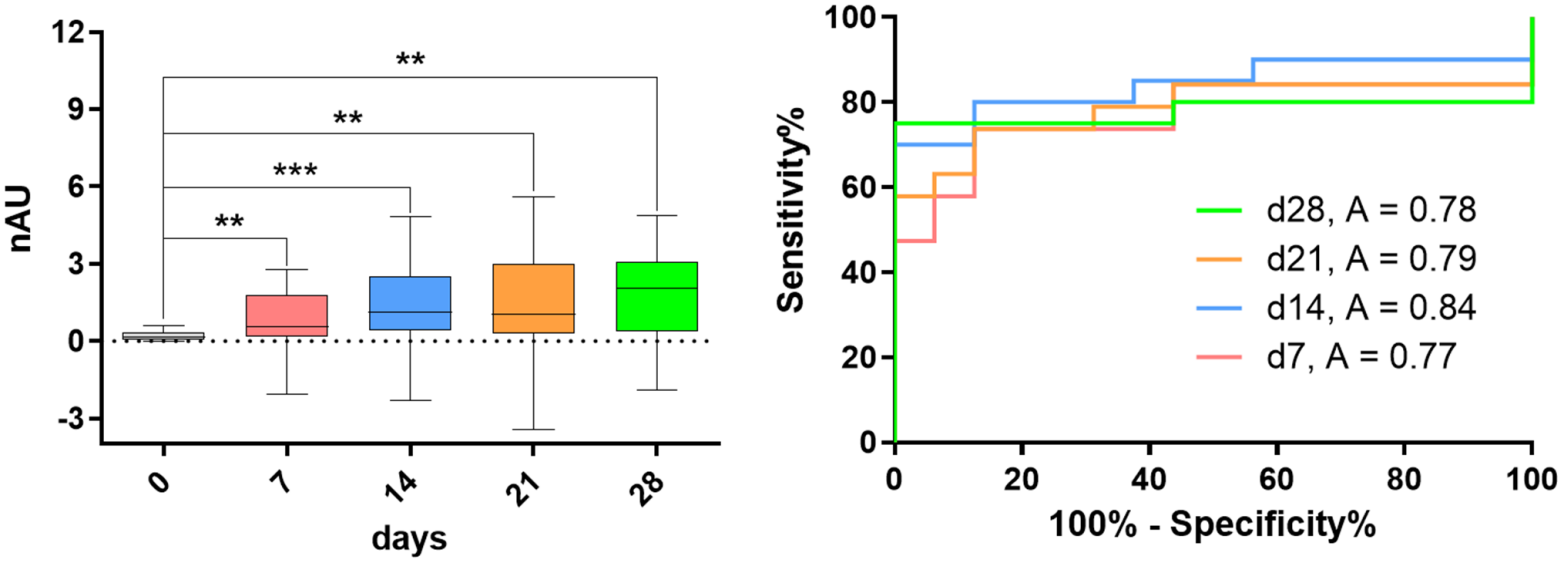
IgM antibodies from mouse sera recognize tetra-galactoside C: *left*: Box-Plot indicating an increase in GPI fragment recognition after infection; *right*: ROC curve for the same synthetic structure; ns *P* > 0.05; * *P* < 0.05; ** *P* < 0.01, *** *P* < 0.001, **** *P* < 0.0001

We prepared glycan microarrays using the synthetic compounds and initially incubated them with sera derived from 10 naïve and 40 AnTat1.1E-infected C57Bl/6 mice. Using a fluorescent secondary antibody, we performed a glycan recognition analysis (Fig. 1c) and observed low fluorescence levels for all antibody classes, indicating low anti-glycan antibody levels (SI). However, quantification and statistical evaluation revealed a three-fold increase in fluorescence levels for IgM and IgG antibodies from infected mice sera at days 7, 14, 21, and 28 post-infection (Fig. 2, SI). These findings suggested the presence of antibodies recognizing GPI structures from VSGs [7]. Further data evaluation was necessary to determine whether synthetic **A**-**F** structures are antigens suitable for detecting a trypanosome infection.

First, the IgM response was analyzed against the naïve control using ANOVA and an unpaired t-test (Fig. 2 and Supporting Information [SI]). For structures that showed significant recognition, a receiver operating characteristic (ROC) curve and the corresponding confidence intervals were calculated for the complete serum set (SI). ROC curves for α-galactoside **A** (average A = 0.79), tetra-galactoside **C** (A = 0.80), and tri-mannoside **D** (A = 0.75) identified these structures as potential diagnostic markers. The sensitivity and specificity determined in the in-lab trial test for α-galactoside **A** (74.85%/94.74%), tetra-galactoside **C** (66.51%/ 93.75%), and tri-mannoside **D** (66.84%/94.74%) are strong and suggest a high probability ratio for distinguishing infected and healthy specimens through the serological determination of specific anti-GPI antibodies in trypanosome-infected mice (SI).

We then analyzed the synthetic glycan recognition by IgG antibodies (SI). The ROC analysis of this study revealed significant values for α-galactoside **A** (average A = 0.70; 61.20%/95%) and tri-mannoside **D** (A = 0.83; 76.20%/94.44%), confirming their suitability to be recognized by antibodies found only in the sera of infected specimens (SI). With these promising results, we were motivated to investigate whether these findings could be transferred to the analysis of patient sera and endemic controls from sub-Saharan Africa, leading to the diagnosis of HAT based on antibody binding to small regions of the trypanosome surface antigens.

### Synthetic GPI fragments are diagnostic antigens of human African trypanosomiasis

In collaboration with the WHO trypanosome database at the Institut Pasteur, we obtained serum from *Tbg-* and *Tbr-*infected humans [42]. The initial datasets used in this study consisted of ten samples each from the endemic control group, as well as from stage 1 and stage 2 of the two distinct parasite infections.

**Fig. 3:**
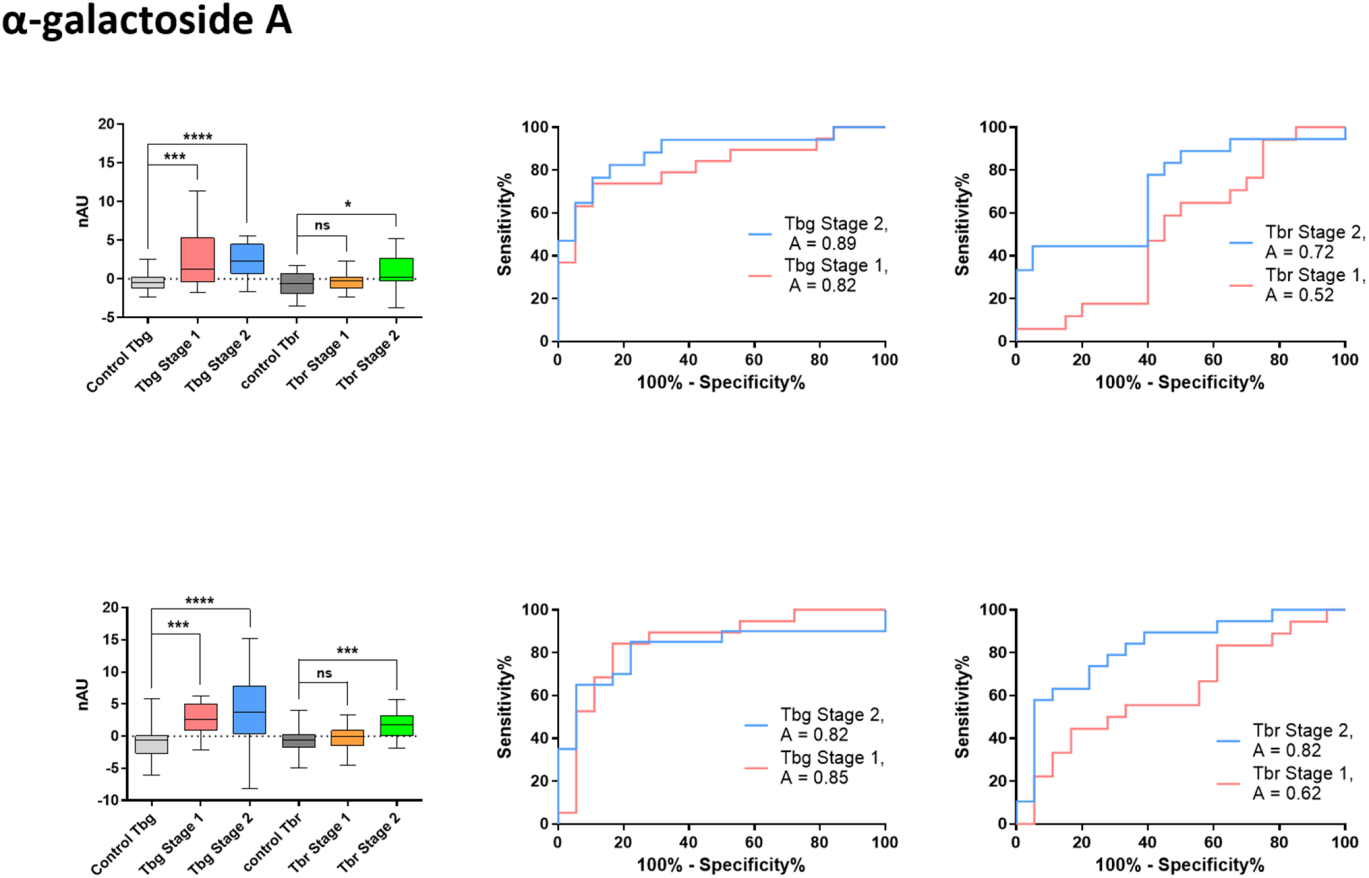
IgM antibodies in human sera from *Tbg-* or *Tbg*-infected patients recognize α-galactoside A and tetra-galactoside C: 1st row: Box-Plot and ROC curves indicating an increase in GPI fragment **A** recognition in specimens with *Tbg* and a weaker recognition focusing on stage 2 with *Tbr* infection; 2nd row: GPI fragment **C** recognition in specimens with *Tbg* and a weaker recognition focusing on stage 2 with *Tbr* infection; nsP > 0.05; * P < 0.05; ** P < 0.01, *** P < 0.001, **** P < 0.0001

Initial observations showed higher structure recognition and fluorescence levels compared to the analysis of mouse sera. A five-fold increase in fluorescence between endemic controls and infected specimens further indicated the recognition of synthetic GPI structures by human antibodies (Fig. 3). Similar to the analysis of mouse sera, the levels of IgM antibodies were statistically evaluated, enabling stage-independent detection of *Tbg* infection through the recognition of α-galactoside **A**, tetra-galactoside **C**, tetra-saccharide **E**, and peptide **F** (Table 1, Fig. 3 and SI). In contrast, IgM-mediated detection of *Tbr* infection was only feasible in sera from patients in the second disease stage, with α-galactoside **A** and tetra-galactoside **C** again providing the best results (SI).

**Fig. 4:**
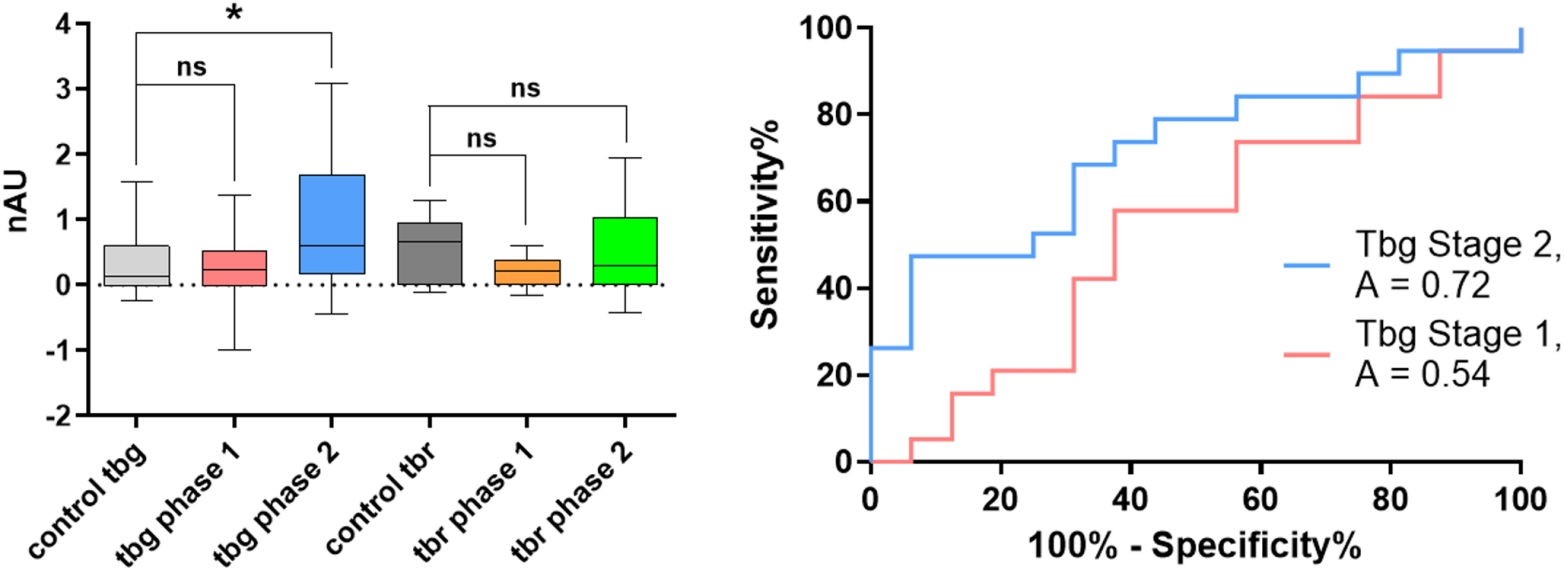
IgG antibodies in human sera from *Tbg*-infected patients and endemic controls interact with tetra-galactoside C: Box-Plot and ROC curves show an increase in GPI fragment **C** recognition in specimens with *Tbg* phase 2 infection; ns *P* > 0.05; * *P* < 0.05; ** *P* < 0.01, *** *P* < 0.001, **** *P* < 0.0001

An analysis of glycan recognition by IgG antibodies to detect *Tbg* infections identified tetra-galactoside **C** (Table 2 and Fig. 4) and tetra-saccharide **E** as the optimal structures for distinguishing infected from healthy individuals in disease stage 2 only (SI). None of the structures synthesized in this study exhibited potential in detecting *Tbr* infections based on anti-glycan IgG determinations.

**Table 1:** Sensitivity, specificity, corresponding confidence intervals, and alternative likelihood ratios of a test using IgM antibodies in human Sera from *Tbg-* or *Tbr*-infected patients and endemic controls that recognize synthetic substances A and C

Structure/Stage		Sensitivity%	95% CI	Specificity%	95% CI	Likelihood ratio
***Tb gambiense***						
**A**	**Stage 1**	63.16	38.36–83.71%	94.74	73.97–99.87%	12.00
	**Stage 2**	64.71	38.33–85.79%	94.74	73.97–99.87%	12.29
**C**	**Stage 1**	52.63	28.86–75.55%	94.44	72.71–99.86%	9.47
	**Stage 2**	65.00	40.78–84.61%	94.44	72.71–99.86%	11.70
***Tb rhodesiense***						
**A**	**Stage 1**	52.94	27.81–77.02%	55.00	31.53–76.94%	1.18
	**Stage 2**	44.44	21.53–69.24%	95.00	75.13–99.87%	8.89
**C**	**Stage 1**	22.22	6.409–47.64%	94.44	72.71–99.86%	4.00
	**Stage 2**	57.89	33.50–79.75%	94.44	72.71–99.86%	10.42

**Table 2:** Sensitivity, specificity, and corresponding confidence intervals and alternative likelihood ratios of a test using IgG antibodies in human Sera from *Tbg*-infected patients and endemic controls recognizing synthetic substance C

Structure/Stage		Sensitivity%	95% CI	Specificity%	95% CI	Likelihood ratio
***Tb gambiense***						
**C**	**Stage 1**	57.89	33.50–79.75%	62.50	35.43–84.80%	1.54
	**Stage 2**	47.37	24.45–71.14%	93.75	69.77–99.84%	7.58

## Discussion and conclusion

In contrast to other protozoan parasites that present free GPIs on their cell membranes, *T. brucei* GPIs are covered by a dense layer of glycoproteins [44]. Thus, it is essential to determine whether a host immune system can elicit an immune response against the buried glycolipid anchor and which structural domains are involved. VSGs and other surface proteins have been used to diagnose infections reliably [30, 31]. However, the high antigenic variation of the VSGs and limited stability of proteins in rural areas pose a challenge to this strategy.

This study focused on determining antibodies specific to synthetic galactosylated GPI structures, which offer greater chemical stability than glycoproteins [34, 35]. The approach relies on the cleavage of membrane-anchored VSG-GPI conjugates— typically through phosphoinositide phospholipase C-mediated hydrolysis— resulting in the release of a soluble form of the VSG (sVSG) that retains the glycosylinositolphosphate (GIP) glycan, commonly referred to as the GIP-sVSG. This process may expose the GPI glycans structures to the host immune system, eliciting an immune response [45]. Although only a limited number of *T. brucei* GPI anchor versions are known [43], a wide range of VSG sets are processed during hydrolysis or phagocytosis [15]. These variations may lead to a polyclonal antibody response to the limited GPI modifications, which may facilitate the diagnosis of trypanosome infections. To evaluate this immune response, we selected microarrays to assess GPI-glycan antibody recognition in serum samples.

Antibodies from *T. brucei-*infected mice recognized the small synthetic GPI structures. The sera of infected mice presented anti-glycan IgM antibodies binding to the structures α-galactoside **A**, tetra-galactoside **C**, and tri-mannoside **D.** IgM levels effectively distinguished the sera of naïve mice from infected animals several weeks post-infection (Figs. 2 and 3). Moreover, the IgM response progressively increased during the course of infection, suggesting that the murine immune response likely encountered many VSG-GPI variants [15]. Evaluation of the IgG antibodies also showed a difference in response between infected and healthy specimens, where infection-induced IgG effectively recognized α-galactoside **A** and tri-mannoside **D**. The sensitivity for both IgM and IgG antibodies was comparable and moderate, with values ranging from 66.51 to 74.85% for IgM and 61.20–76.20% for IgG. Although these levels are typically observed in early-stage antibody-based diagnoses of trypanosome infection [30, 31], the specificity determined in our test meets current field standards (85–97% for CATT, 96–99% for LATEX) [45–50]. Synthetic structures **A**, **C** and **D** show specificity values between 93.75% and 94.74% for IgM and 94.44% and 95.00% for IgG. The overlap of the structures giving the best serostatus differentiation suggests using galactosylated structures and the non-mammalian GPI backbone to detect trypanosome infections in mice.

Based on these promising results, the glycan array of structures **A-F** was used to screen a set of human sera from endemic controls and *Tbg* and *Tbr-*infected patients from disease stages 1 and 2. Sera from *Tbg* patients showed IgM antibody recognition of structures **A**,** C**,** E** and **F**., suggesting that these antigens could be considered for diagnosis regardless of the infection stage. The detection presented a moderate sensitivity, ranging from 35 to 80%, and high specificity, ranging from 90 to 95%. In contrast, only compounds **A** and **C** were diagnostic antigens for infections with *Tbr*, and this was only in the second stage of the disease. The test sensitivity was lower compared to IgM, with values ranging from 44 to 58%, but it maintained a high specificity, ranging from 94.5 to 95.0%. Using IgG as a readout for *Tbg* infection, compounds **C** and **E** showed the best results with sensitivities of 47% and 67%, respectively, and a specificity of 94%.

The results suggest that fully synthetic GPI structures can be used as diagnostic antigens for HAT, primarily caused by *Tbg* infections. The inability to detect *Tbr* infections may be associated with the heterogeneity of the released GPI structures or the sample size. However, apart from the fact that the Mannose-I residue may bear between two and four galactoses in any *T. brucei* species, there are currently no reports in the literature indicating differences in the glycosylation patterns [32, 51]. Another potential confounding factor could arise from the differences in immune responses between the two parasites. For instance, in contrast to *Tbr* (which causes an aggressive and acute infection lasting for several weeks to months), *Tbg* leads to a more progressive disease (lasting up to three years) with less inflammation, which has been correlated with less B-cell loss [52]. Thus, the difference in antibody signals observed between the two infections (stage 1 and stage 2) may be associated with differences in B-cell responses due to variations in inflammatory immune responses.

The recognition of multiple structures suggests the possibility of improving the test sensitivity through structure optimization. In contrast, the high test specificity indicates the effectiveness of GPI antigens in reducing false negatives. Summarizing all the analyses, the tetra-αgalactoside structure **C** arises as the most suitable structure for diagnosing infected individuals from distinct geographical areas.

When analyzing structures **A** and **C**, it became clear that **C** contains the terminal α-galactose structure twice. Thus, it can be regarded as a multivalent display of the precursor structure **A** with enhanced binding affinity and improved selectivity. Compared to others, the apparent improved immunogenicity of these galactoside structures may lie in the enrichment of the screened compound set.

The specific function or biological relevance of adding α-(1–3) galactose to the GPIs within the parasite or mammalian host remains unclear. However, this carbohydrate moiety is a post-translational modification that may contribute to the proper protein folding, stability and intracellular movement of proteins. Additionally, humans and some primates lack the enzyme needed to produce the α(1–3) galactose (α-Gal) epitope, a carbohydrate found on the surface of many pathogens and mammalian cells. As a result, this glycosylation triggers an immunogenic response in humans, leading to the production of IgG and IgM antibodies against α-Gal, which can provide some protection against pathogens carrying this epitope. Notably, humans can develop IgG antibodies specific to this antigen [53] due to continued environmental exposure to the galactose-α-1,3-galactose epitope found in red meat or fat. Moreover, people with α-galactose syndrome are allergic to galactose-α-1,3-galactose by mounting a strong IgE response. Hence, trypanosomes may exploit this mechanism to trigger and amplify the humoral immune response against the VSG-GPI component as a decoy strategy, aiming to exhaust the immune system and drive antibody production toward regions (i.e. α(1–3) galactose (α-Gal) epitope) that are not or less exposed on intact parasites. In addition, VSGs form a dense coat on the surface of the trypanosome, serving as a macromolecular diffusion barrier for the parasite [54]. The predicted three-dimensional structure of the GPI suggests that it could significantly contribute to the surface coat integrity as a diffusion barrier [55]. The anchors on mature VSGs contain a heterogenously branched structure of α-galactose attached to the 3-*O* position of the mannose residue adjacent to the glucosamine. Since the galactosylation levels correlate with VSG subclass, the galactose residues may play a space-filling role in accommodating the different three-dimensional structures of the carboxyl-terminal domains. These variations may allow more efficient packaging of the VSG anchor at the cell surface [56]. 

The positive results obtained with compound **D** in the analysis of human sera do not necessarily reflect an exclusive response to trypanosome-derived GPI. This structure is conserved in the GPIs of other protozoan parasites, such as *Toxoplasma gondii* and *Plasmodium falciparum*, and the binding may result from cross-reactivity with antibodies from different infections [25, 27] In addition, humans with Chagas disease and certain gut microbacteria may carry antibodies against α-galactoside structures [57, 58], highlighting the need for specific antigens. Further characterization of the sera and a record of previous patient infections should help to clarify the origin of the recognition.

Compound **E** was immobilized using the phosphoethanolamine moiety; as a result, the structure was attached closer to the glass surface and reverted to the natural orientation of GPIs on the cell membrane. This orientation did not impact antibody binding, and a broad recognition by antibodies was still observed. These results suggest a certain level of VSG-GPI release via hydrolysis, a phagocytosis mechanism, or overlapping epitope recognition. Future applications in a field trial may prioritize diagnosing infection over determining stage. Under these conditions, compounds **A** and **C** are crucial for establishing serostatus and enabling an IgM-based diagnosis for HAT. Our findings indicate that using fragments of complex GPI structures in *T. brucei* for glycan-based diagnostics presents a cost-effective and stable solution for future clinical applications and developing point-of-care (PoC) devices for HAT diagnostics. Thus, future studies must tackle two main challenges: optimizing the glycan structure and presentation to enhance sensitivity, and identifying the appropriate diagnostic technology for PoC devices. Synthetic compounds will facilitate optimization without incurring high antigen production costs and will allow for improved analysis through larger quantities of glycans or alternative presentation systems, such as beads or modified nanomaterials. Additionally, different glycan presentations enable integration with bead-based and electrochemical sensors [59, 60], which are highly suitable for PoC diagnostics and compatible with enzymatic processes to amplify the recognition signal and improve sensitivity.

One limitation of the analysis performed in this study is the number of sera used. Future investigations may elaborate on this test and increase the subject number to overcome the sensitivity limitations. Establishing a diagnostic test based on fully synthetic antigens may support or replace serological tests using recombinant antigens and parasite cultivation-dependent requirements [61, 62]. 

## Electronic supplementary material

Below is the link to the electronic supplementary material.

Supplementary Material 1

Supplementary Material 2 Supplementary Material 2

Supplementary Material 3 Supplementary Material 3

Supplementary Material 4 Supplementary Material 4

Supplementary Material 5 Supplementary Material 5

Supplementary Material 6 Supplementary Material 6

Supplementary Material 7 Supplementary Material 7

Supplementary Material 8 Supplementary Material 8

Supplementary Material 9 Supplementary Material 9

## Data Availability

Data is provided within the manuscript or supplementary information files.
