# Supplementary material for: Detection of antibodies against the African parasite Trypanosoma brucei using synthetic glycosylphosphatidylinositol oligosaccharide fragments
Source: Glycoconj J. 2025 Jun 24;42(3-4):147–58. doi: 10.1007/s10719-025-10186-x (PMC12350571; doi:10.1007/s10719-025-10186-x)
Supplement: Supplementary file 1 — Supplementary Material 1 [file 10719_2025_10186_MOESM1_ESM.docx]

**Detection of Antibodies Against the African Parasite *Trypanosoma brucei* Using Synthetic glycosylphosphatidylinositol oligosaccharide fragments**

Maurice Michel,^[a, b, c, *]^ Benoît Stijlemans,^[d, e]^ Dana Michel,^[a, b]^ Monika Garg,^[a, b]^ Andreas Geissner,^[a, b]^ Peter H. Seeberger^[a, b]^ and Daniel Varón Silva^[a, b, f *]^

1. Max-Planck-Institute of Colloids and Interfaces, Biomolecular Systems Department, Am Mühlenberg 1, 14476 Potsdam (Germany)
2. Freie Universität Berlin, Department of Biology, Chemistry and Pharmacy, Arnimallee 22, 14195 Berlin (Germany)
3. Department of Oncology and Pathology, Karolinska Institutet, Science for Life Laboratory, Tomtebodavägen 23A, 17121 Stockholm (Sweden)

Brussels Center for Immunology, Vrije Universiteit Brussel, 1050 Brussels, Belgium.

Myeloid Cell Immunology Laboratory, VIB Center for Inflammation Research, 1050 Brussels, Belgium.

1. School of Life Sciences FHNW, Institute for Chemistry and Bioanalytics, 4132 Muttenz, Switzerland.

* Corresponding author: daniel.varon@fhnw.ch and maurice.michel@ki.se

**Readout Microarray**

**IgM Mouse**

**
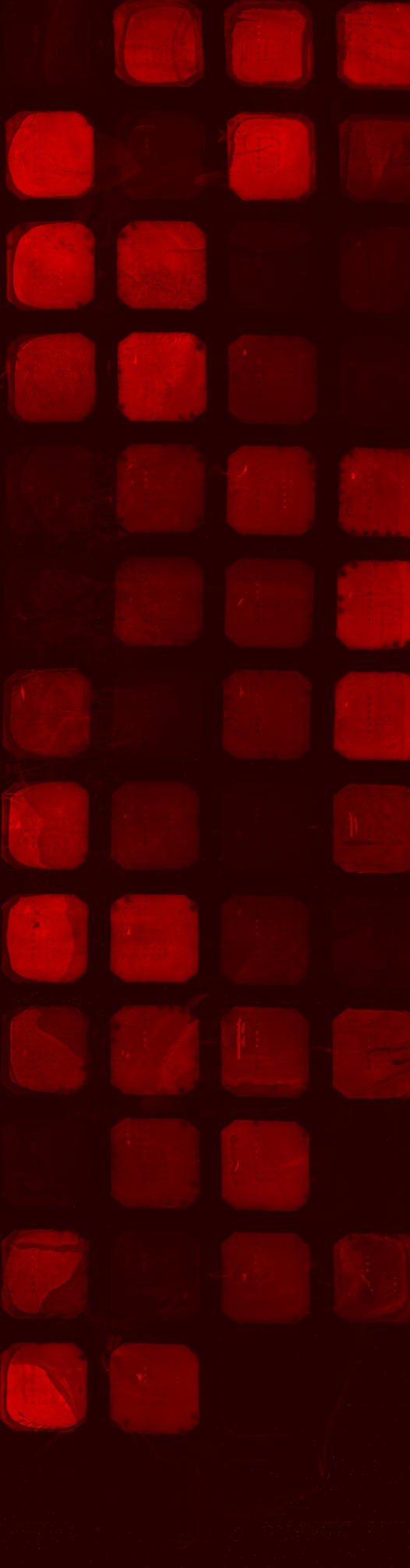

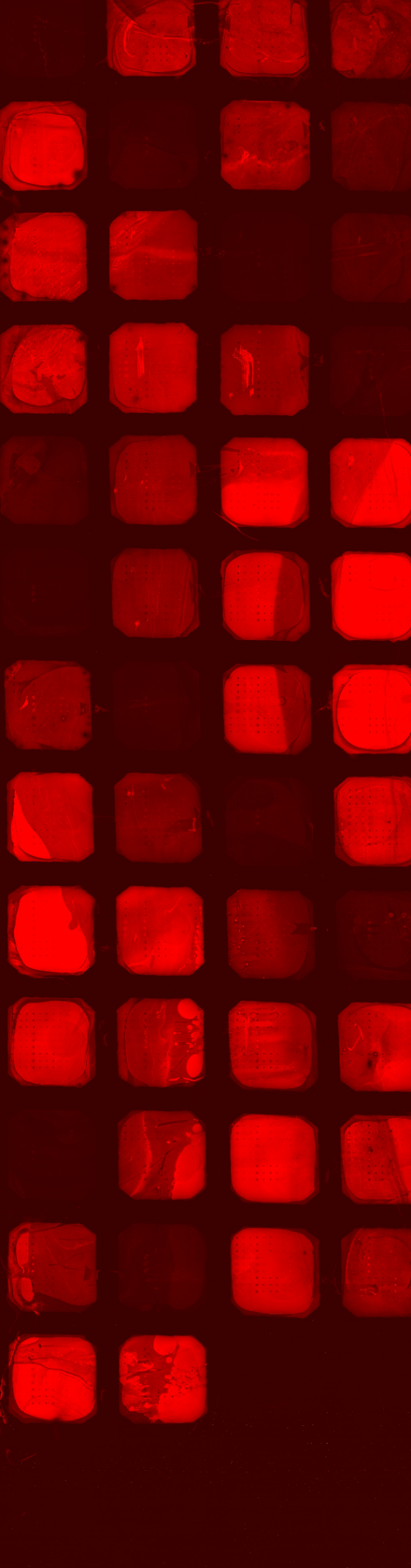
**

**IgG Mouse**

**
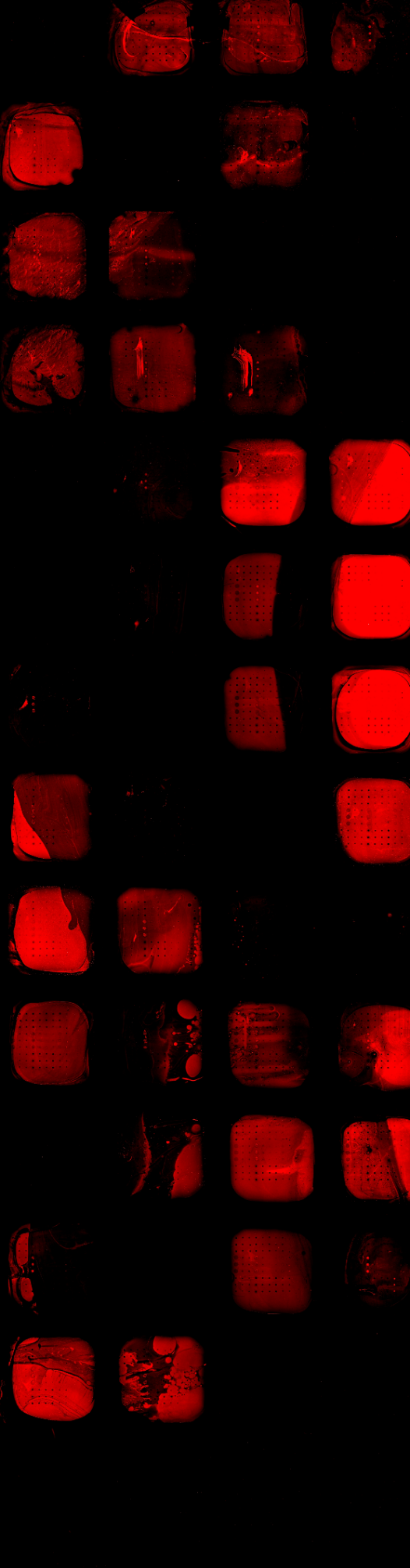

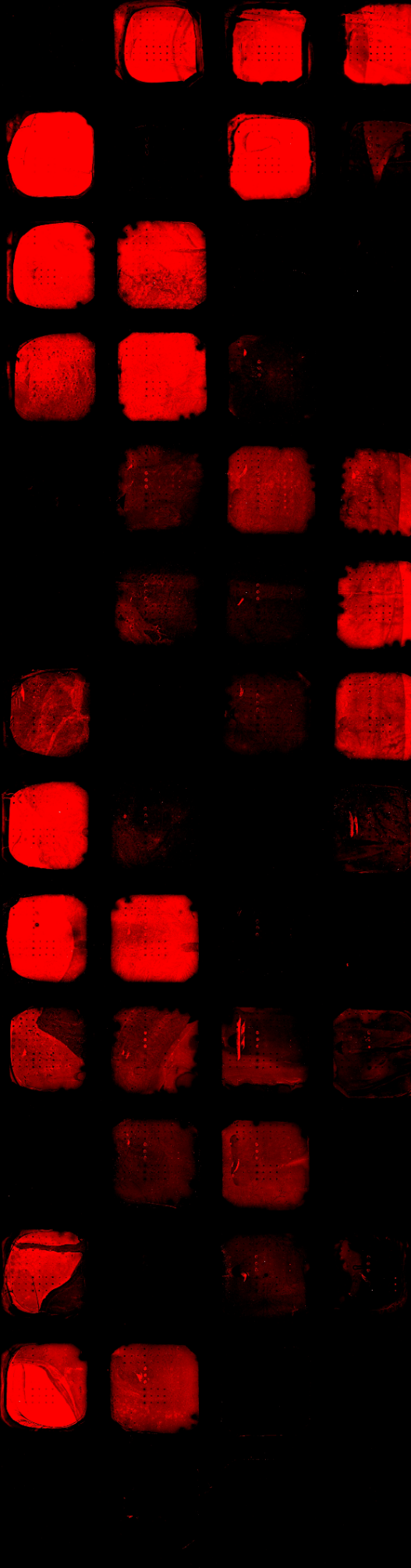
**

**IgM human**

**
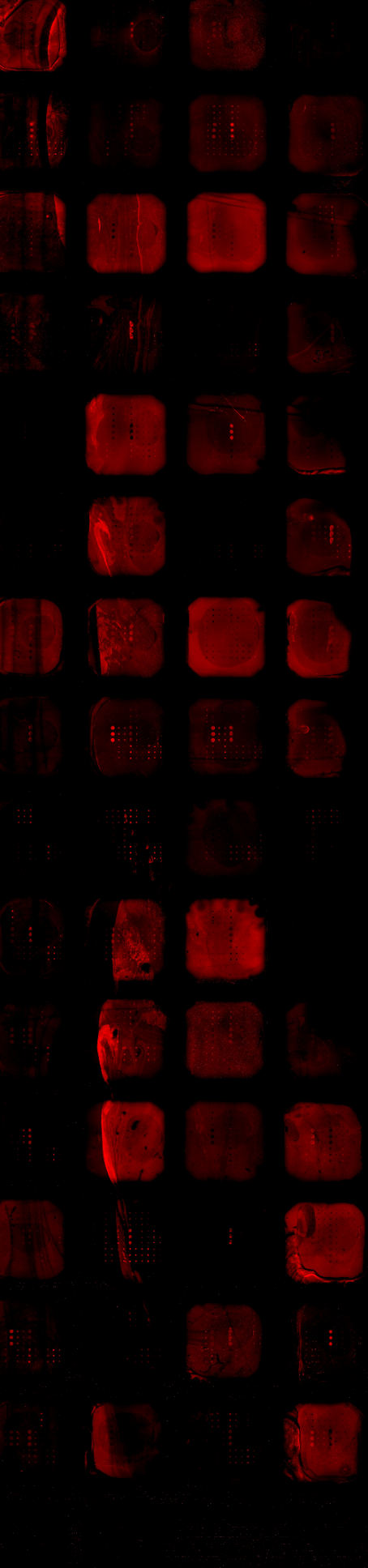

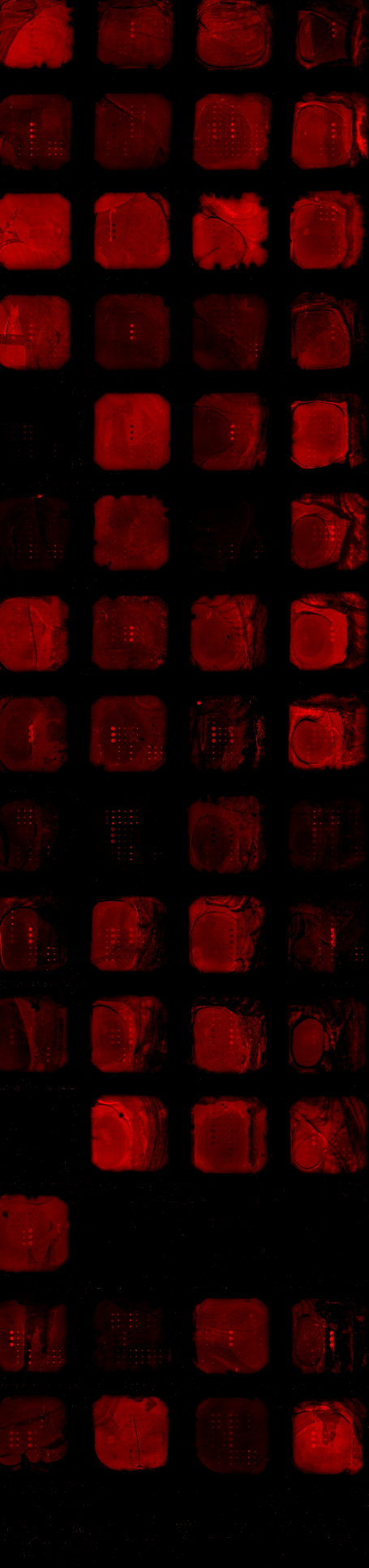
**

**IgG human**

**
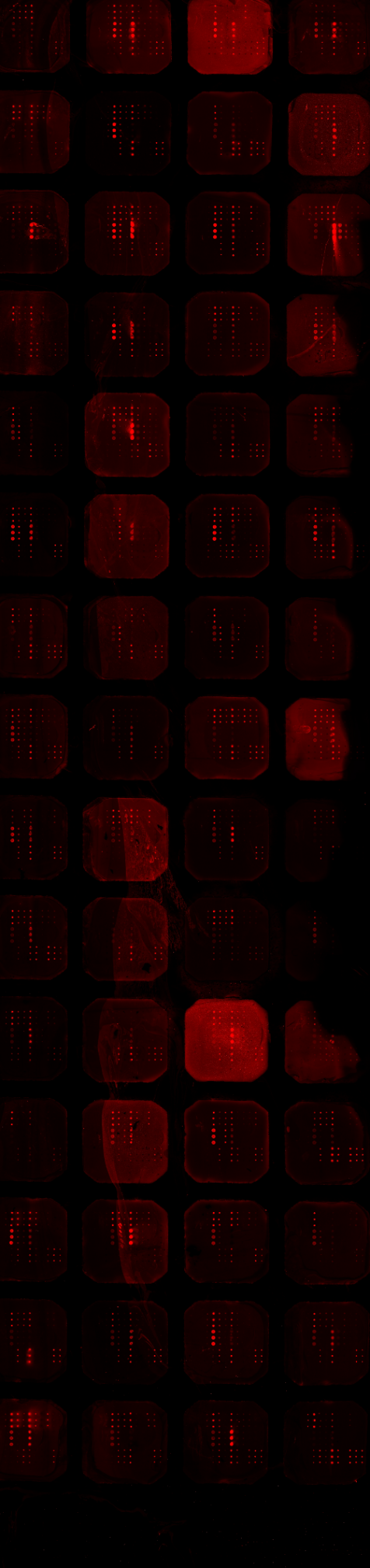

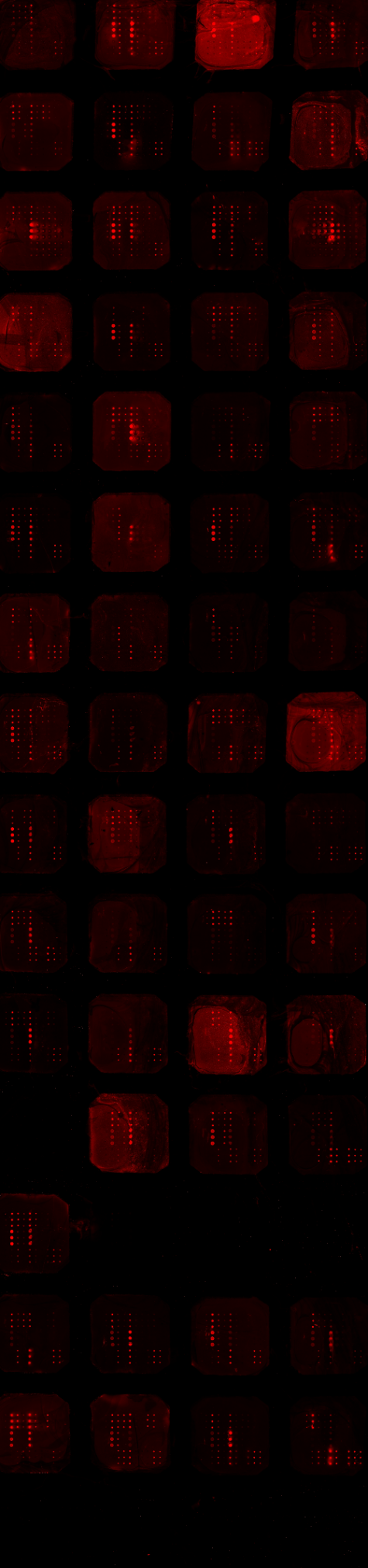
**

**Data Evaluation: Box-Plot and ROC curve for all data sets.** Comparative Box-Plot of normalized fluorescence levels caused by antibody recognition by sera of different diseases stages, i.e. mouse naïve, d7, d14, d21 and d21 and human stage 1 and stage 2. Using GraphPadPrism 7.0, a significance test was performed using Tukey, followed by a two-way ANOVA and the calculation of receiver operation characteristics (ROC) curves. The area under the ROC curve was determined for the capability of the diagnostic tool to distinguish between A) non-infected and infected individuals and B) whether IgM and/or IgG are suitable antibody classes for this purpose.

**IgM Mouse**

**IgG Mouse**

**IgM human**

**IgG human**

**Diagnostic power**

Sensitivity, specificity and the respective 95% confidence intervals (CI) using the criteria of highest likelihood ratio for *T. brucei gambiense* and *T. brucei rhodesiense* infections are calculated as descriptors during generation of ROC curves. In a clinical setting, high likelihood ratios are preferred. This expectation allows for a moderate sensitivity as long as high specificity is maintained.

**IgM mouse**

| **Structure/day** | **Sensitivity%** | **95% CI** | **Specificity%** | **95% CI** | **Likelihood ratio** |
| --- | --- | --- | --- | --- | --- |
|  |  |  |  |  |  |
| Gal-Man d7 | 63,16 | 38,36% to 83,71% | 94,74 | 73,97% to 99,87% | 12,00 |
| Gal-Man d14 | 93,75 | 69,77% to 99,84% | 94,74 | 73,97% to 99,87% | 17,81 |
| Gal-Man d21 | 62,50 | 35,43% to 84,80% | 94,74 | 73,97% to 99,87% | 11,88 |
| Gal-Man d28 | 80,00 | 56,34% to 94,27% | 94,74 | 73,97% to 99,87% | 15,20 |
|  |  |  |  |  |  |
| Gal-Gal-Man d7 | 33,33 | 13,34% to 59,01% | 94,74 | 73,97% to 99,87% | 6,33 |
| Gal-Gal-Man d14 | 50,00 | 27,20% to 72,80% | 94,74 | 73,97% to 99,87% | 9,50 |
| Gal-Gal-Man d21 | 53,33 | 26,59% to 78,73% | 94,74 | 73,97% to 99,87% | 10,13 |
| Gal-Gal-Man d28 | 35,29 | 14,21% to 61,67% | 94,74 | 73,97% to 99,87% | 6,71 |
|  |  |  |  |  |  |
| Gal-Gal-(Gal)-Gal d7 | 57,89 | 33,50% to 79,75% | 93,75 | 69,77% to 99,84% | 9,26 |
| Gal-Gal-(Gal)-Gal d14 | 70,00 | 45,72% to 88,11% | 93,75 | 69,77% to 99,84% | 11,20 |
| Gal-Gal-(Gal)-Gal d21 | 63,16 | 38,36% to 83,71% | 93,75 | 69,77% to 99,84% | 10,11 |
| Gal-Gal-(Gal)-Gal d28 | 75,00 | 50,90% to 91,34% | 93,75 | 69,77% to 99,84% | 12,00 |
|  |  |  |  |  |  |
| P-Man-Man-Man d7 | 80,00 | 56,34% to 94,27% | 94,74 | 73,97% to 99,87% | 15,20 |
| P-Man-Man-Man d14 | 84,21 | 60,42% to 96,62% | 94,74 | 73,97% to 99,87% | 16,00 |
| P-Man-Man-Man d21 | 40,00 | 19,12% to 63,95% | 94,74 | 73,97% to 99,87% | 7,60 |
| P-Man-Man-Man d28 | 63,16 | 38,36% to 83,71% | 94,74 | 73,97% to 99,87% | 12,00 |
|  |  |  |  |  |  |
| VSG117ctdp d7 | 85,00 | 62,11% to 96,79% | 95,00 | 75,13% to 99,87% | 17,00 |
| VSG117ctdp d14 | 40,00 | 19,12% to 63,95% | 95,00 | 75,13% to 99,87% | 8,00 |
| VSG117ctdp d21 | 75,00 | 50,90% to 91,34% | 95,00 | 75,13% to 99,87% | 15,00 |
| VSG117ctdp 28 | 30,00 | 11,89% to 54,28% | 95,00 | 75,13% to 99,87% | 6,00 |
|  |  |  |  |  |  |
| Gal-(p)-Man-(Gal)-Man d7 | 47,06 | 22,98% to 72,19% | 94,12 | 71,31% to 99,85% | 8,00 |
| Gal-(p)-Man-(Gal)-Man d14 | 55,00 | 31,53% to 76,94% | 94,12 | 71,31% to 99,85% | 9,35 |
| Gal-(p)-Man-(Gal)-Man d21 | 36,84 | 16,29% to 61,64% | 94,12 | 71,31% to 99,85% | 6,26 |
| Gal-(p)-Man-(Gal)-Man d28 | 38,89 | 17,30% to 64,25% | 94,12 | 71,31% to 99,85% | 6,61 |

**IgG Mouse**

| Structure/day | Sensitivity% | 95% CI | Specificity% | 95% CI | Likelihood ratio |
| --- | --- | --- | --- | --- | --- |
|  |  |  |  |  |  |
| Gal-Man d7 | 52,63 | 28,86% to 75,55% | 95,00 | 75,13% to 99,87% | 10,53 |
| Gal-Man d14 | 68,42 | 43,45% to 87,42% | 95,00 | 75,13% to 99,87% | 13,68 |
| Gal-Man d21 | 43,75 | 19,75% to 70,12% | 95,00 | 75,13% to 99,87% | 8,75 |
| Gal-Man d28 | 80,00 | 56,34% to 94,27% | 95,00 | 75,13% to 99,87% | 16,00 |
|  |  |  |  |  |  |
| Gal-Gal-Man d7 | 70,00 | 45,72% to 88,11% | 94,44 | 72,71% to 99,86% | 12,60 |
| Gal-Gal-Man d14 | 55,00 | 31,53% to 76,94% | 94,44 | 72,71% to 99,86% | 9,90 |
| Gal-Gal-Man d21 | 65,00 | 40,78% to 84,61% | 94,44 | 72,71% to 99,86% | 11,70 |
| Gal-Gal-Man d28 | 38,89 | 17,30% to 64,25% | 94,44 | 72,71% to 99,86% | 7,00 |
|  |  |  |  |  |  |
| Gal-Gal-(Gal)-Gal d7 | 55,56 | 30,76% to 78,47% | 93,75 | 69,77% to 99,84% | 8,89 |
| Gal-Gal-(Gal)-Gal d14 | 52,63 | 28,86% to 75,55% | 93,75 | 69,77% to 99,84% | 8,42 |
| Gal-Gal-(Gal)-Gal d21 | 55,56 | 30,76% to 78,47% | 93,75 | 69,77% to 99,84% | 8,89 |
| Gal-Gal-(Gal)-Gal d28 | 50,00 | 27,20% to 72,80% | 93,75 | 69,77% to 99,84% | 8,00 |
|  |  |  |  |  |  |
| P-Man-Man-Man d7 | 89,47 | 66,86% to 98,70% | 94,44 | 72,71% to 99,86% | 16,11 |
| P-Man-Man-Man d14 | 94,74 | 73,97% to 99,87% | 94,44 | 72,71% to 99,86% | 17,05 |
| P-Man-Man-Man d21 | 50,00 | 27,20% to 72,80% | 94,44 | 72,71% to 99,86% | 9,00 |
| P-Man-Man-Man d28 | 70,59 | 44,04% to 89,69% | 94,44 | 72,71% to 99,86% | 12,71 |
|  |  |  |  |  |  |
| VSG117ctdp d7 | 75,00 | 50,90% to 91,34% | 93,33 | 68,05% to 99,83% | 11,25 |
| VSG117ctdp d14 | 65,00 | 40,78% to 84,61% | 93,33 | 68,05% to 99,83% | 9,75 |
| VSG117ctdp d21 | 50,00 | 27,20% to 72,80% | 93,33 | 68,05% to 99,83% | 7,50 |
| VSG117ctdp 28 | 75,00 | 50,90% to 91,34% | 93,33 | 68,05% to 99,83% | 11,25 |
|  |  |  |  |  |  |
| Gal-(p)-Man-(Gal)-Man d7 | 63,16 | 38,36% to 83,71% | 94,74 | 73,97% to 99,87% | 12,00 |
| Gal-(p)-Man-(Gal)-Man d14 | 65,00 | 40,78% to 84,61% | 94,74 | 73,97% to 99,87% | 12,35 |
| Gal-(p)-Man-(Gal)-Man d21 | 63,16 | 38,36% to 83,71% | 94,74 | 73,97% to 99,87% | 12,00 |
| Gal-(p)-Man-(Gal)-Man d28 | 60,00 | 36,05% to 80,88% | 94,74 | 73,97% to 99,87% | 11,40 |

**IgM human**

| Structure/Disease Stage | Sensitivity% | 95% CI | Specificity% | 95% CI | Likelihood ratio |
| --- | --- | --- | --- | --- | --- |
|  |  |  |  |  |  |
| Tb gambiense |  |  |  |  |  |
| Gal-Man Stage 1 | 63,16 | 38,36% to 83,71% | 94,74 | 73,97% to 99,87% | 12,00 |
| Gal-Man Stage 2 | 64,71 | 38,33% to 85,79% | 94,74 | 73,97% to 99,87% | 12,29 |
|  |  |  |  |  |  |
| Gal-Gal-Man Stage 1 | 21,05 | 6,052% to 45,57% | 94,44 | 72,71% to 99,86% | 3,79 |
| Gal-Gal-Man Stage 2 | 77,78 | 52,36% to 93,59% | 55,56 | 30,76% to 78,47% | 1,75 |
|  |  |  |  |  |  |
| Gal-Gal-(Gal)-Gal Stage 1 | 52,63 | 28,86% to 75,55% | 94,44 | 72,71% to 99,86% | 9,47 |
| Gal-Gal-(Gal)-Gal Stage 2 | 65,00 | 40,78% to 84,61% | 94,44 | 72,71% to 99,86% | 11,70 |
|  |  |  |  |  |  |
| p-Man-Man-Man Stage 1 | 40,00 | 19,12% to 63,95% | 90,00 | 68,30% to 98,77% | 4,00 |
| p-Man-Man-Man Stage 2 | 21,05 | 6,052% to 45,57% | 95,00 | 75,13% to 99,87% | 4,21 |
|  |  |  |  |  |  |
| Gal-(p)-Man-(Gal)-Man Stage 1 | 65,00 | 40,78% to 84,61% | 94,12 | 71,31% to 99,85% | 11,05 |
| Gal-(p)-Man-(Gal)-Man Stage 2 | 80,00 | 56,34% to 94,27% | 94,12 | 71,31% to 99,85% | 13,60 |
|  |  |  |  |  |  |
| VSG117ctdp Stage 1 | 35,00 | 15,39% to 59,22% | 95,00 | 75,13% to 99,87% | 7,00 |
| VSG117ctdp Stage 2 | 55,00 | 31,53% to 76,94% | 90,00 | 68,30% to 98,77% | 5,50 |
|  |  |  |  |  |  |
| Tb rhodesiense |  |  |  |  |  |
| Gal-Man Stage 1 | 52,94 | 27,81% to 77,02% | 55,00 | 31,53% to 76,94% | 1,18 |
| Gal-Man Stage 2 | 44,44 | 21,53% to 69,24% | 95,00 | 75,13% to 99,87% | 8,89 |
|  |  |  |  |  |  |
| Gal-Gal-Man Stage 1 | 93,33 | 68,05% to 99,83% | 50,00 | 26,02% to 73,98% | 1,87 |
| Gal-Gal-Man Stage 2 | 100,00 | 79,41% to 100,0% | 38,89 | 17,30% to 64,25% | 1,64 |
|  |  |  |  |  |  |
| Gal-Gal-(Gal)-Gal Stage 1 | 22,22 | 6,409% to 47,64% | 94,44 | 72,71% to 99,86% | 4,00 |
| Gal-Gal-(Gal)-Gal Stage 2 | 57,89 | 33,50% to 79,75% | 94,44 | 72,71% to 99,86% | 10,42 |
|  |  |  |  |  |  |
| p-Man-Man-Man Stage 1 | 70,00 | 45,72% to 88,11% | 60,00 | 36,05% to 80,88% | 1,75 |
| p-Man-Man-Man Stage 2 | 21,05 | 6,052% to 45,57% | 90,00 | 68,30% to 98,77% | 2,11 |
|  |  |  |  |  |  |
| Gal-(p)-Man-(Gal)-Man Stage 1 | 31,58 | 12,58% to 56,55% | 95,00 | 75,13% to 99,87% | 6,32 |
| Gal-(p)-Man-(Gal)-Man Stage 2 | 25,00 | 8,657% to 49,10% | 95,00 | 75,13% to 99,87% | 5,00 |
|  |  |  |  |  |  |
| VSG117ctdp Stage 1 | 33,33 | 13,34% to 59,01% | 88,89 | 65,29% to 98,62% | 3,00 |
| VSG117ctdp Stage 2 | 10,53 | 1,301% to 33,14% | 94,44 | 72,71% to 99,86% | 1,90 |

**IgG human**

| Structure/Disease Stage | Sensitivity% | 95% CI | Specificity% | 95% CI | Likelihood ratio |
| --- | --- | --- | --- | --- | --- |
|  |  |  |  |  |  |
| Tb gambiense |  |  |  |  |  |
| Gal-Man Stage 1 | 87,50 | 61,65% to 98,45% | 35,29 | 14,21% to 61,67% | 1,35 |
| Gal-Man Stage 2 | 55,56 | 30,76% to 78,47% | 82,35 | 56,57% to 96,20% | 3,15 |
|  |  |  |  |  |  |
| Gal-Gal-Man Stage 1 | 12,50 | 1,551% to 38,35% | 93,75 | 69,77% to 99,84% | 2,00 |
| Gal-Gal-Man Stage 2 | 93,75 | 69,77% to 99,84% | 37,50 | 15,20% to 64,57% | 1,50 |
|  |  |  |  |  |  |
| Gal-Gal-(Gal)-Gal Stage 1 | 57,89 | 33,50% to 79,75% | 62,50 | 35,43% to 84,80% | 1,54 |
| Gal-Gal-(Gal)-Gal Stage 2 | 47,37 | 24,45% to 71,14% | 93,75 | 69,77% to 99,84% | 7,58 |
|  |  |  |  |  |  |
| p-Man-Man-Man Stage 1 | 16,67 | 3,578% to 41,42% | 94,12 | 71,31% to 99,85% | 2,83 |
| p-Man-Man-Man Stage 2 | 35,00 | 15,39% to 59,22% | 94,12 | 71,31% to 99,85% | 5,95 |
|  |  |  |  |  |  |
| Gal-(p)-Man-(Gal)-Man Stage 1 | 27,78 | 9,695% to 53,48% | 93,75 | 69,77% to 99,84% | 4,44 |
| Gal-(p)-Man-(Gal)-Man Stage 2 | 60,00 | 36,05% to 80,88% | 93,75 | 69,77% to 99,84% | 9,60 |
|  |  |  |  |  |  |
| VSG117ctdp Stage 1 | 35,00 | 15,39% to 59,22% | 75,00 | 50,90% to 91,34% | 1,40 |
| VSG117ctdp Stage 2 | 50,00 | 27,20% to 72,80% | 75,00 | 50,90% to 91,34% | 2,00 |
|  |  |  |  |  |  |
| Tb rhodesiense |  |  |  |  |  |
| Gal-Man Stage 1 | 75,00 | 47,62% to 92,73% | 73,68 | 48,80% to 90,85% | 2,85 |
| Gal-Man Stage 2 | 22,22 | 6,409% to 47,64% | 94,74 | 73,97% to 99,87% | 4,22 |
|  |  |  |  |  |  |
| Gal-Gal-Man Stage 1 | 35,00 | 15,39% to 59,22% | 95,00 | 75,13% to 99,87% | 7,00 |
| Gal-Gal-Man Stage 2 | 22,22 | 6,409% to 47,64% | 95,00 | 75,13% to 99,87% | 4,44 |
|  |  |  |  |  |  |
| Gal-Gal-(Gal)-Gal Stage 1 | 100,00 | 80,49% to 100,0% | 55,00 | 31,53% to 76,94% | 2,22 |
| Gal-Gal-(Gal)-Gal Stage 2 | 41,18 | 18,44% to 67,08% | 75,00 | 50,90% to 91,34% | 1,65 |
|  |  |  |  |  |  |
| p-Man-Man-Man Stage 1 | 17,65 | 3,799% to 43,43% | 94,74 | 73,97% to 99,87% | 3,35 |
| p-Man-Man-Man Stage 2 | 53,33 | 26,59% to 78,73% | 94,74 | 73,97% to 99,87% | 10,13 |
|  |  |  |  |  |  |
| Gal-(p)-Man-(Gal)-Man Stage 1 | 25,00 | 7,266% to 52,38% | 90,00 | 68,30% to 98,77% | 2,50 |
| Gal-(p)-Man-(Gal)-Man Stage 2 | 33,33 | 13,34% to 59,01% | 95,00 | 75,13% to 99,87% | 6,67 |
|  |  |  |  |  |  |
| VSG117ctdp Stage 1 | 40,00 | 19,12% to 63,95% | 80,00 | 56,34% to 94,27% | 2,00 |
| VSG117ctdp Stage 2 | 25,00 | 8,657% to 49,10% | 90,00 | 68,30% to 98,77% | 2,50 |

**Synthetic structures**

*6-(benzylthio)hexyl 2,3,4-O-tri-benzyl-6-O-chloroacetyl-α-D-galactopyranosyl-(1→3)-2-O-acetyl-4-O-benzyl-6-O-tert-butyldiphenylsilyl-α-D-mannopyranoside* **(4)**

*Step 1*

10 mg of [IrCOD(PMePh_2_)_2_]PF_6_ were added to 2 ml THF. Hydrogen was bubbled through the suspension until the catalyst dissolved. The solution was transferred to a second flask, where it dissolved disaccharide **1**[1] (0.050 mmol, 0.055 g). The reaction was stirred at room temperature overnight. THF was evaporated under reduced pressure and the residue was dissolved in an 8:1 mixture of acetone and water. Mercury oxide (0.005 mmol, 1.0 mg) and mercury chloride (0.250 mmol, 67.8 mg) were added and the solution was stirred for one hour at room temperature. The reaction was quenched by adding saturated NaHCO_3_ ‑solution and the resulting mixture was extracted with DCM. The combined organic phases were dried over Na_2_SO_4_, filtered and concentrated under reduced pressure. The residue was purified by silica column chromatography using hexane and ethyl acetate as eluent. Product **2** was obtained in 65% yield (0.032 mmol, 0.034 g) as colorless oil. **R_f_** = 0.1 (4:1, Hex/AcOEt); **^1^H‑NMR** (400 MHz, CDCl_3_): δ = 7.66 – 7.62 (m, 2H, H_Ar._), 7.55 (dt, *J*= 6.8 Hz, 1.4 Hz, 2H, H_Ar._), 7.35 – 7.17 (m, 16H, H_Ar._), 7.16 – 6.98 (m, 10H, H_Ar._), 5.25 (d, *J*= 3.6 Hz, 1H, Gal‑1), 5.13 – 5.09 (m, 2H, Man‑1, Man‑2), 5.00 (d, *J*= 11.6 Hz, 1H, -CH_2_-), 4.86 (d, *J*= 11.6 Hz, 1H, -CH_2_-), 4.77 – 4.67 (m, 2H, -CH_2_-), 4.62 (d, *J*= 12.0 Hz, 1H, -CH_2_-), 4.57 – 4.48 (m, 3H, -CH_2_-), 4.27 – 4.17 (m, 2H), 4.10 – 4.04 (m, 2H), 4.01 – 3.96 (m, 4H), 3.93 – 3.78 (m, 5H), 3.73 (dd, *J* = 11.4, 1.7 Hz, 1H), 2.02 (s, 3H, -CH_3_), 1.01 (s, 9H, -C(-CH_3_)_3_) ppm; **^13^C‑NMR** (101 MHz, CDCl_3_): δ = 170.8 (C=O), 167.3 (C=O), 138.8 (C_Ar._), 138.6 (C_Ar._), 138.4 (C_Ar._), 138.1 (C_Ar._), 136.1 (C_Ar._), 135.6 (C_Ar._), 134.0 (C_Ar._), 133.2 (C_Ar._), 129.8 (C_Ar._), 129.7 (C_Ar._), 128.6 (C_Ar._), 128.6 (C_Ar._), 128.3 (C_Ar._), 128.3 (C_Ar._), 128.0 (C_Ar._), 127.8 (C_Ar._), 127.7 (C_Ar._), 127.7 (C_Ar._), 127.6 (C_Ar._), 127.6 (C_Ar._), 127.5 (C_Ar._), 127.3 (C_Ar._), 126.9 (C_Ar._), 126.7 (C_Ar._), 99.7 (Gal‑1), 92.4 (Man‑1), 78.8, 77.5, 77.4, 77.2, 76.8, 75.6, 75.0, 74.7, 74.5, 74.5, 74.3, 73.5, 73.1, 73.0, 72.6, 69.2, 65.6, 62.8, 40.9, 27.0, 21.2, 19.6 ppm; **ESI-MS**: m/z M_calcd_ for C_60_H_67_ClO_13_Si = 1058.4039; M_found_ = 1081.3901 [M+Na]^+^; **[α]_D_^20^** = 40.07 (c = 0.1 g/L in CHCl_3_); **FTIR**: ν = 2934.63, 1745.15, 1455.60, 1241.04, 1061.56 cm^-1^.

*Step 2*

Hemiacetal **2** (0.032 mmol, 0.034 g) was dissolved in DCM at 0°C. Trichloroacetonitrile (0.256 mmol, 25.7 µl) and DBU (0.003 mmol, 0.5 µl) were added and the reaction was stirred until TLC indicated full conversion. The resulting mixture was concentrated under reduced pressure and was purified by silica column chromatography using hexane and ethyl acetate as eluent. Product **3** was obtained in 84% yield (0.027 mmol, 0.033 g) as colorless oil and was used directly for the next step. **R_f_** = 0.45 (4:1, Hex/AcOEt); **^1^H‑NMR** (400 MHz, CDCl_3_): δ = 8.62 (s, 1H, =NH), 7.63 (dt, *J*= 6.9 Hz, 1.5 Hz, 2H, H_Ar._), 7.58 – 7.53 (m, 2H, H_Ar._), 7.37 – 7.01 (m, 26H), 6.29 (d, *J*= 2.0 Hz, 1H, Man‑1), 5.24 (d, *J*= 2.3 Hz, 1H, Gal‑1), 5.13 – 5.07 (m, 2H, Man‑2, -CH_2_-), 4.89 (d, *J*= 11.4 Hz, 1H, -CH_2_-), 4.82 – 4.63 (m, 4H, -CH_2_-), 4.58 – 4.50 (m, 3H), 4.19 – 4.16 (m, 2H), 4.10 – 3.75 (m, 11H), 2.05 (s, 3H, -CH_3_), 1.00 (s, 9H, -C(-CH_3_)_3_) ppm.

*Step 3*

Imidate **3** (0.027 mmol, 0.033 g) and 6-Thiobenzylhexanol (0.012 g, 0.054 mmol) were coevaporated three times with toluene, dried under high vacuum and dissolved in DCM. Freshly activated powdered molecular sieves were added and the suspension was stirred for 10 minutes. TMSOTf (0.008 mmol, 1.5 µl) was added at 0°C. After TLC indicated full conversion, the reaction was quenched by adding saturated NaHCO_3_-solution and extracted with DCM. The combined organic phases were dried over MgSO_4_, filtered and evaporated. The residue was purified by silica gel chromatography giving product **5** quantitative yield (0.027 mmol, 0.034 g) as colorless oil. **R_f_** = 0.45 (4:1, Hex/AcOEt); **^1^H‑NMR** (400 MHz, CDCl_3_): δ = 7.64 (dt, *J*= 6.7 Hz, 1.5 Hz, 2H, H_Ar._), 7.57 (dt, *J*= 6.8 Hz, 1.4 Hz, 2H, H_Ar._), 7.35 – 7.06 (m, 29H, H_Ar._), 7.00 – 6.96 (m, 2H, H_Ar._), 5.19 (d, *J*= 3.6 Hz, 1H, Gal‑1), 5.04 (dd, *J*= 3.4 Hz, 1.7 Hz, 1H, Man‑1), 4.98 (d, *J*= 11.4 Hz, 1H, -CH_2_-), 4.87 (d, *J*= 11.6 Hz, 1H, -CH_2_-), 4.77 – 4.68 (m, 4H, -CH_2_-, Man‑1), 4.66 (d, *J*= 12.1 Hz, 1H, ‑CH_2_-), 4.55 – 4.50 (m, 2H, -CH_2_-), 4.47 (d, *J*= 11.5 Hz, 1H, -CH_2_-), 4.22 – 4.08 (m, 2H), 4.04 – 3.73 (m, 13H), 3.66 – 3.40 (m, 2H), 3.29 (dt, *J*= 9.7 Hz, 6.8 Hz, 1H), 2.37 – 2.27 (m, 4H, -CH_2_-), 2.00 (s, 3H, -CH_3_), 1.57 – 1.12 (m, 8H), 0.99 (s, 9H) ppm; **^13^C‑NMR** (101 MHz, CDCl_3_): δ = 170.8 (C=O), 167.0 (C=O), 138.8 (C_Ar._), 138.6 (C_Ar._), 138.4 (C_Ar._), 138.2 (C_Ar._), 136.1 (C_Ar._), 135.7 (C_Ar._), 133.9 (C_Ar._), 133.2 (C_Ar._), 129.7 (C_Ar._), 129.7 (C_Ar._), 128.9 (C_Ar._), 128.6 (C_Ar._), 128.6 (C_Ar._), 128.5 (C_Ar._), 128.3 (C_Ar._), 128.3 (C_Ar._), 128.3 (C_Ar._), 128.0 (C_Ar._), 127.8 (C_Ar._), 127.7 (C_Ar._), 127.7 (C_Ar._), 127.6 (C_Ar._), 127.6 (C_Ar._), 127.5 (C_Ar._), 127.3 (C_Ar._), 127.1 (C_Ar._), 127.0 (C_Ar._), 127.0 (C_Ar._), 100.0 (Gal‑1), 97.1 (Man‑1), 78.8, 77.5, 77.4, 77.2, 76.8, 76.6, 75.7, 74.8, 74.7, 74.5, 74.3, 73.5, 73.1, 72.8, 72.6, 69.1, 67.7, 65.4, 64.6, 62.9, 40.9, 36.4, 36.4, 31.4, 29.9, 29.4, 29.2, 28.8, 28.6, 28.6, 26.9, 25.8, 25.7, 21.2, 19.5 ppm; **ESI-MS**: m/z M_calcd_ for C_73_H_85_ClO_13_SSi: 1264.5169; M_found_ = 1287.5035 [M+Na]^+^; **[α]_D_^20^** = 65.75 (c = 0.1 g/L in CHCl_3_); **FTIR**: ν = 3032.53, 2932.25, 2859.17, 2211.79, 2162.52, 1743.16, 1496.93, 1454.99, 1429.28, 1364.17, 1239.58, 1135.16, 1101.34, 1059.38, 1028.84, 824.05, 798.05, 737.48, 700.11 cm^-1^.

*6-Mercaptohexyl α-D-galactopyranosyl-(1→3)-α-D-mannopyranoside* ***(A)***

*Step 1*

Disaccharide **5** (0.027 mmol, 0.034 g) was dissolved in a mixture of DCM and Methanol. Acetyl chloride (0.1 ml) was added dropwise and the reaction mixture was stirred until TLC indicated full conversion. The green reaction was diluted with DCM, quenched with saturated NaHCO_3_-solution and extracted with DCM. The combined organic phases were dried over MgSO_4_, filtered and evaporated. The residue was purified by silica gel chromatography giving product **6** in 48% (0.013 mmol, 0.012 g) as colorless oil. **R_f_** = 0.2 (2:1, Hex/AcOEt); **^1^H‑NMR** (400 MHz, CDCl_3_): δ = 7.34 – 7.07 (m, 25H, H_Ar._), 5.06 – 5.01 (m, 2H, -CH_2_-, Gal‑1), 4.86 (d, *J*= 11.7 Hz, 1H, -CH_2_-), 4.77 – 4.58 (m, 5H, -CH_2_-, Man‑1), 4.53 – 4.48 (m, 2H, -CH_2_-), 4.09 – 3.99 (m, 3H), 3.90 – 3.79 (m, 3H), 3.78 – 3.65 (m, 3H), 3.62 (s, 2H), 3.60 – 3.43 (m, 3H), 3.32 – 3.21 (m, 2H), 2.33 (t, *J*= 7.3 Hz, 2H), 1.52 – 1.41 (m, 4H), 1.31 -1.17 (m, 4H) ppm; **^13^C‑NMR** (101 MHz, CDCl_3_): δ = 138.5 (C_Ar._), 138.4 (C_Ar._), 138.4 (C_Ar._), 138.2 (C_Ar._), 128.8 (C_Ar._), 128.5 (C_Ar._), 128.4 (C_Ar._), 128.4 (C_Ar._), 128.3 (C_Ar._), 128.3 (C_Ar._), 127.9 (C_Ar._), 127.8 (C_Ar._), 127.7 (C_Ar._), 127.6 (C_Ar._), 127.6 (C_Ar._), 127.5 (C_Ar._), 126.8 (C_Ar._), 100.0 (Gal‑1), 98.7 (Man‑1), 82.5, 78.5, 77.3, 77.2, 77.0, 76.9, 76.7, 75.3, 74.9, 74.3, 73.3, 73.2, 73.1, 71.7, 71.4, 69.7, 67.7, 63.1, 61.9, 36.2, 31.2, 29.2, 29.0, 28.6, 25.7 ppm; **ESI-MS**: m/z M_calcd_ for C_53_H_64_O_11_S = 908.4169; M_found_ = 931.4075 [M+Na]^+^; **[α]_D_^20^** = 30.76 (c = 0.1 g/L in CHCl_3_); **FTIR**: ν = 3393.73, 3032.10, 2930.62, 2163.30, 2036.82, 1497.01, 1454.56, 1352.26, 1096.52, 1068.04, 1043.31, 738.63, 698.46, 682.08, 660.41 cm^-1^.

*Step 2*

Ammonia (10 ml) was condensed in a flask at ‑78°C and two drops of methanol were added. Sodium was added in small pieces until a dark blue color was established. Triol **6** (0.013 mmol, 0.012 g) was dissolved in 1 ml THF and added to the ammonia solution. The reaction was stirred for 1 h, subsequently adding more sodium when the blue color disappeared. The reaction was quenched by adding methanol and ammonia was blown off using a stream of nitrogen. The pH of the resulting solution was adjusted to 7-8 with glacial acetic acid. The residue was concentrated under reduced pressure and purified by size exclusion using 5% ethanol in water as eluent and RP-HPLC (hypercarb column 150×10mm, ThermoFisher, 5 µ, acetonitrile in water 0-100% in 60 min). Product **A** was obtained in 50% yield (6.540 µmol, 0.3 mg) as white solid. **^1^H-NMR** (400 MHz, D_2_O): δ = 5.12 (d, *J* = 4.1 Hz, 1H, Gal‑1), 4.72 (1H, Man‑1), 4.01 – 3.21 (m, 16H), 1.51 – 1.42 (m, 4H), 1.24 – 1.05 (m, 4H) ppm; **^13^C-NMR** (151 MHz, D_2_O): δ = 100.0 (Gal‑1), 99.4 (Man‑1), 72.7, 71.2, 69.1, 68.6, 66.4, 66.0, 64.6, 30.7, 21.9 ppm; **ESI-MS**: m/z M_calcd_ for C_18_H_34_O_11_S = 458.1822; M_found_ = 937.3439 [2M+Na]^+^

*2,3,4-O-tri-benzyl-6-O-chloroacetyl-α-D-galactopyranosyl-(1→6)-2,3,4-O-tri-benzyl-α-D-galactopyranosyl-(1→3)-2-O-acetyl-4-O-benzyl-6-O-tert-butyldiphenylsilyl-α-D-mannopyranosyl trichloracetimidate* (**9**)

*Step 1*

Hydrogen was bubbled through a suspension of 10 mg of [IrCOD(PMePh_2_)_2_]PF_6_ in 2 ml THF until the catalyst was dissolved. The solution was transferred to a second flask containing trisaccharide **7**[1] (0.014 mmol, 0.022 g) and the resulting reaction mixture was stirred under hydrogen atmosphere at room temperature overnight. The THF was evaporated under reduced pressure and the residue was dissolved in an 8:1 mixture of acetone and water. Mercury oxide (0.001 mmol, 0.2 mg) and mercury chloride (0.070 mmol, 19.0 mg) were added and the mixture was stirred for one hour at room temperature. The reaction was quenched by adding saturated NaHCO_3_ ‑solution and the resulting mixture was extracted with DCM. The combined organic phases were dried over Na_2_SO_4_, filtered and concentrated under reduced pressure. The residue was purified by silica column chromatography using hexane and ethyl acetate as eluent. Product **8** was obtained in 45% yield (6.430 µmol, 9.6 mg) as colorless oil. **R_f_** = 0.1 (4:1, Hex/AcOEt); **^1^H‑NMR** (400 MHz, CDCl_3_): δ = 7.64 (d, *J*= 7.3 Hz, 2H, H_Ar._), 7.55 (d, *J*= 7.3 Hz, 2H, H_Ar._), 7.39 – 6.99 (m, 41H, H_Ar._), 5.29 (s, 1H, Gal‑1), 5.06 (s, 1H, Man‑1), 4.95 (d, *J*= 11.8 Hz, 1H, Gal‘‑1), 4.88 – 4.80 (m, 3H, Gal‑1, Gal‘‑6, -CH_2_-), 4.76 – 4.42 (m, 13H, ‑CH_2_-), 4.36 – 4.22 (m, 2H), 4.11 (s, 1H, Man‑1), 4.06 – 3.61 (m, 16H), 3.08 (d, *J*= 10.1 Hz, Man-6), 2.00 (s, 3H, -CH_3_), 0.98 (s, 9H, -C(-CH_3_)_3_) ppm; **^13^C‑NMR** (101 MHz, CDCl_3_): δ = 170.8 (C=O), 167.2 (C=O), 138.9 (C_Ar._), 138.7 (C_Ar._), 138.6 (C_Ar._), 138.5 (C_Ar._), 138.3 (C_Ar._), 138.2 (C_Ar._), 138.1 (C_Ar._), 137.5 (C_Ar._), 136.2 (C_Ar._), 135.7 (C_Ar._), 129.6 (C_Ar._), 128.6 (C_Ar._), 128.6 (C_Ar._), 128.5 (C_Ar._), 128.5 (C_Ar._), 128.4 (C_Ar._), 128.3 (C_Ar._), 128.2 (C_Ar._), 128.2 (C_Ar._), 128.1 (C_Ar._), 128.0 (C_Ar._), 127.8 (C_Ar._), 127.7 (C_Ar._), 127.7 (C_Ar._), 127.7 (C_Ar._), 127.6 (C_Ar._), 127.6 (C_Ar._), 127.4 (C_Ar._), 127.3 (C_Ar._), 126.9 (C_Ar._), 99.1 (Gal‑1), 97.3 (Gal‑1), 92.0 (Man‑1), 79.0, 78.6, 77.5, 77.4, 77.2, 76.8, 75.7, 75.2, 74.9, 74.6, 74.5, 73.9, 73.4, 73.4, 73.3, 73.1, 72.8, 72.6, 70.0, 68.0, 64.9, 62.9, 40.8 (-CClH_2_), 26.9 (-*C*(-CH_3_)_3_), 21.4 (‑C(‑*C*H_3_)_3_), 19.6 (-CH_3_) ppm; **ESI-MS**: m/z M_calcd_ for C_87_H_95_ClO_18_Si = 1490.5976; M_found_ = 1513.5905 [M+Na]^+^; **[α]_D_^20^** = 54.35 (c = 0.1 g/L in CHCl_3_); **FTIR**: ν = 3453.56, 3033.13, 2930.22, 2172.92, 2128.27, 2037.40, 1966.26, 1738.57, 1497.85, 1455.58, 1429.05, 1361.71, 1241.71, 1103.51, 1059.90, 1028.48, 825.28, 739.79, 698.76, 664.27 cm^-1^.

*Step 2*

Hemiacetal **8** (6.430 μmol, 9.6 mg) was dissolved in DCM at 0°C. Trichloroacetonitrile (0.051 mmol, 5.1 µl) and DBU (0.643 μmol, 0.1 µl) were added and the reaction was stirred for until TLC indicated full conversion. The resulting mixture was concentrated under reduced pressure and was purified by silica column chromatography using hexane and ethyl acetate as eluent. Imidate **9** was obtained in 95% yield (6.110 µmol, 10.0 mg) as colorless oil and used for the next step. **R_f_** = 0.6 (4:1, Hex/AcOEt); **^1^H‑NMR** (400 MHz, CDCl_3_): δ = 8.58 (s, 1H, =NH), 7.67 – 7.55 (m, 5H), 7.38 – 7.01 (m, 40H), 6.25 (s, 1H, Man‑1), 5.24 – 5.13 (m, 2H), 4.94 – 4.38 (m, 22H), 4.18 – 3.76 (m, 24H), 3.60 – 3.43 (m, 3H), 1.99 (s, 3H, -CH_3_), 1.00 (s, 9H, -C(-CH_3_)_3_) ppm.

*6-Mercaptohexyl α-D-galactopyranosyl-(1→6)-α-D-galactopyranosyl-(1→3)-α-D-mannopyranoside* **(B)**

*Step 1*

Imidate **9** (6.110 μmol, 10.0 mg) and 6-thiobenzyl-1-hexanol (0.031 mmol, 7.0 mg) were coevaporated three times with toluene, dried under high vacuum and dissolved in DCM. Freshly activated powdered molecular sieves were added and the suspension was stirred for 10 minutes. TMSOTf (0.002 μmol, 0.4 µl) was added and the reaction was stirred at 0°C. After TLC indicated full conversion, the reaction was quenched by adding saturated NaHCO_3_-solution and extracted with DCM. The combined organic phases were dried over MgSO_4_, filtered and evaporated. The residue was purified by silica gel chromatography giving trisaccharide **10** in 67% yield (4.120 µmol, 7.0 mg) as colorless oil. The compound was directly used for the next step. **R_f_** = 0.50 (4:1, Hex/AcOEt); **^1^H‑NMR** (400 MHz, CDCl_3_): δ = 7.74 – 7.60 (m, 4H, H_Ar._), 7.42 – 7.07 (m, 46H, H_Ar._), 5.45 – 4.44 (m, 18H), 4.25 – 3.25 (m, 23H), 2.41 – 2.31 (m, 2H), 2.02 (s, 1.5H, -CH_3_), 1.99 (s, 1.5H, -CH_3_), 1.63 – 1.18 (m, 8H), 1.04 (s, 9H, -C(-CH_3_)_3_) ppm.

*Step 2*

Trisaccharide **10** (4.120 μmol, 7.0 mg) was dissolved in a mixture of DCM and Methanol. Acetyl chloride (0.1 mL) was added dropwise and the reaction was stirred until TLC indicated full conversion. The green reaction solution was diluted with DCM, quenched with saturated NaHCO_3_-solution and extracted with DCM. The combined organic phases were dried over MgSO_4_, filtered and evaporated. The residue was purified by silica gel chromatography giving triol **11** in 83% yield (3.430 µmol, 4.6 mg) as colorless oil. **R_f_** = 0.2 (2:1, Hex/AcOEt); **^1^H-NMR** (400 MHz, CDCl_3_): δ = 7.72 – 7.69 (m, 1H, H_Ar._), 7.53 - 7.51 (m, 1H, H_Ar._), 7.42 – 7.18 (m, 38H, H_Ar._), 5.12 – 3.08 (m, 39H), 2.36 – 2.33 (m, 2H), 1.42 – 1.19 (m, 8H) ppm; **ESI-MS**: m/z M_calcd_ for C_80_H_92_O_16_S = 1340.6106; M_found_ = 1363.5980 [M+Na]^+^; **[α]_D_^20^** = 10.54 (c = 0.1 g/L in CHCl_3_); **FTIR**: ν = 3400.89, 2925.13, 2855.51, 2310.45, 2219.20, 2196.67, 2163.72, 2143.15, 2053.58, 2024.55, 1986.08, 1941.06, 1725.99, 1455.76, 1376.12, 1096.72, 826.02, 737.91, 698.77, 663.35 cm^-1^.

*Step 3*

Ammonia was condensed (10 ml) at ‑78°C and two drops of methanol were added. Sodium was added in small pieces until a dark blue color was established. Triol **11** (3.430 μmol, 4.6 mg) was dissolved in 1 ml THF and added to the ammonia solution. The reaction was stirred for 1 h, subsequently adding more sodium when the blue color disappeared. The reaction was quenched by adding methanol and ammonia was blown off using a stream of nitrogen. The pH of the resulting solution was adjusted to 7-8 with glacial acetic acid. The residue was concentrated under reduced pressure and purified by size exclusion using 5% ethanol in water as eluent to give the trisaccharide **B** in 71% yield (2.440 µmol, 3.0 mg). **^1^H‑NMR** (600 MHz, D_2_O): δ = 5.12 (d, *J*= 4.1 Hz, 1H, Gal‑1), 4.71 (s, 1H, Gal’‑1), 4.65 (d, *J*= 1.9 Hz, 1H, Man‑1), 4.02 – 3.18 (m, 14H), 2.64 (t, *J*= 7.2 Hz, 2H), 1.60 - 1.45 (m, 4H), 1.31 – 1.23 (m, 4H) ppm; **^13^C-NMR** (151 MHz, D_2_O): δ = 100.7, 99.5 (x2), 78.4, 72.6, 71.3, 69.7, 69.2, 68.6, 67.7, 65.9, 61.4, 61.2, 60.7, 38.0, 28.1, 27.0, 24.9 ppm; **ESI-MS**: m/z M_calcd_ for C_24_H_44_O_16_S = 620.2350; M_found_ = 1261.4429 [2M+Na]^+^.

*6-Mercaptohexyl α-D-galactopyranosyl-(1→2)-α-D-galactopyranosyl-(1→6)-2-O-(α-D-galactopyranosyl)-α-D-galactopyranoside* **(C)**

*Step 1*

In a round bottom flask, 10 mg of [IrCOD(PMePh_2_)_2_]PF_6_ were added to 2 mL THF. Hydrogen was bubbled through the suspension until the catalyst dissolved. This solution was transferred to a second flask, where it dissolved of tetrasaccharide **12**[1] (0.080 mmol, 0.200 g). The reaction was stirred at room temperature overnight. THF was evaporated under reduced pressure and the residue was dissolved in an 8:1 mixture of acetone and water. mercury oxide (0.8 µmol, 1.7 mg) and mercury chloride (0.40 mmol, 108.6 mg) were added and the solution was stirred for one hour at room temperature. The reaction was quenched by adding sat. NaHCO_3_ solution and the resulting mixture was extracted three times with DCM. The combined organic phases were dried over Na_2_SO_4_, filtered and concentrated under reduced pressure. The residue was purified by silica column chromatography using hexane and ethyl acetate as eluent. Product **13** was obtained in 81% yield (0.064 mmol, 0.159 g) as colorless oil and directly used for the next step. **R_f_** = 0.1 (3:1, Hex/AcOEt).

*Step 2*

Hemiacetal **13** (0.024 mmol, 0.059 g) was dissolved in DCM at 0°C. Trichloroacetonitril (0.192 mmol, 19.3 µl) and DBU (2.400 µmol, 0.4 µl) were added and the reaction was stirred until TLC indicated full conversion. The resulting mixture was concentrated under reduced pressure and purified by silica column chromatography using hexane and ethyl acetate as eluent. After several reaction cycles Product **14** was obtained in 11% yield (2.680 µmol, 7.000 mg) as colorless oil and used in the next step. **R_f_** = 0.6 (3:1, Hex/AcOEt).

*Step 3*

Imidate **14** (0.019 mmol, 0.050 g) and 6-Thiobenzylhexanol (0.057 mmol. 0.013 g) were co-evaporated three times with toluene and dried under high vacuum. The compound mixture was dissolved in the solvent and 4 Å MS was added. TMSOTf (5.700 µmol, 1.0 µl) was added and the reaction was stirred until TLC indicated full conversion. The reaction mixture was diluted with DCM and quenched by adding sat. NaHCO_3_ solution. The mixture was extracted three times with DCM and the combined organic phases were dried over Na_2_SO_4_, filtered and concentrated under reduced pressure. The residue was purified by silica column chromatography using hexane and ethyl acetate as eluent. Product **15** was obtained in 20% yield (3.740 µmol, 10.0 mg) as colorless oil. **R_f_** = 0.55 (4:1, Hex/AcOEt) **^1^H‑NMR** (400 MHz, CDCl_3_): δ = 7.89 – 6.56 (m, 82H, H_Ar._), 5.85 – 5.77 (m, 2H), 5.52 – 5.46 (m, 2H), 5.16 – 5.04 (m, 5H), 4.95 – 3.03 (m, 53H), 2.30 -2.25 (m, 2H), 1.50 -1.13 (m, 8H) ppm. **^13^C-NMR from HSQC** (101 MHz, CDCl_3_): δ = 131.8, 131.7, 127.7 (x), 127.0 (x), 125.9 (x), 113.4 (x), 96.8, 95.7, 95.3, 94.4, 78.8 (x2), 78.4 (x2), 76.5, 76.4, 76.0, 75.9, 75.3, 74.5 (x6), 74.4 (x2), 73.5 (x8), 73.2, 72.7 (x5), 72.3 (x3), 71.9 (x4), 71.8 (x2), 71.6, 68.5 (x4), 68.3 (x2), 68.1 x4), 67.8 (x2), 60.2, 55.3 (x4), 29.8, 22.6, 20.9, 14.1 (x2) ppm.

*Step 4*

10 mL ammonia were condensed in a flask and methanol (2 drops) was added. Sodium was added in small pieces until a dark blue color established. Tetrasaccharide **15** (3.700 µmol, 10.0 mg) was dissolved in THF and added to the ammonium solution at ‑78°C. At this temperature the reaction was stirred for 1 h. The reaction was quenched by adding methanol and ammonia was blown off using a stream of nitrogen. The pH of the resulting solution was adjusted with glacial acetic acid to 7-8. The reaction concentrated under reduced pressure and the residue was purified by size exclusion using 5% ethanol in water as eluent. A final HPLC purification on a hypercarb column (150×10mm, ThermoFisher, 5 µ) using a 0-100% gradient of acetonitrile in water in 60 min delivered product **C** in 20% (7.480 µmol, 5.860 mg) as white solid. **^1^H‑NMR** (400 MHz, D_2_O): δ = 5.14 – 4.99 (m, 4H), 4.07 – 3.44 (m, 16H), 2.82 – 2.77 (m, 2H), 1.68 – 1.28 (m, 4H) ppm. **ESI-MS**: m/z M_calcd_ for C_30_H_54_O_21_S = 782.2878; M_found_ = 1581.6877 [M_2_+NH_4_]^+^.

*6-(Benzylthio)-hexyl 2-O-acetyl-3,4-di-O-benzyl-6-hydroxy-α-D-mannopyranoside (****18****)*

*Step 1*

A mixture of mannosyl trichloroacetamidate donor **16**[2] (1.138 mmol, 0.800 g) and 6-Thiobenzylhexanol (3.41 mmol, 0.766 g) was coevaporated three times with toluene and dried under high vacuum for 1 h. After that the reaction mixture was dissolved in DCM followed by the addition of molecular sieves. The reaction stirred for 10 min at 0°C and TMSOTf (0.228 mmol, 41.0 µl) was added. After 1.30 h, the reaction mixture was quenched using triethylamine, filtered and concentrated. The residue was purified using silica gel column chromatography to obtain **17** in 72% yield (0.819 mmol, 625 mg) as colorless oil. **R_f_** = 0.6 (4:1, Hex/AcOEt), **^1^H‑NMR** (400 MHz, CDCl_3_): δ = 7.26 – 7.08 (m, 15H), 5.20 (dd, *J* = 3.3, 1.8 Hz, 1H), 4.79 (d, *J* = 10.7 Hz, 1H), 4.64 – 4.58 (m, 2H), 4.53 (d, *J* = 10.7 Hz, 1H), 4.45 (d, *J* = 11.1 Hz, 1H), 3.95 – 3.74 (m, 4H), 3.59 (d, *J* = 2.4 Hz, 2H), 3.52 (qd, *J* = 6.7, 3.7 Hz, 2H), 3.22 (dt, *J* = 9.7, 6.6 Hz, 1H), 2.29 (dd, *J* = 9.3, 5.4 Hz, 2H), 2.00 (s, 3H), 1.42 (dt, *J* = 14.5, 7.7 Hz, 4H), 1.25 – 1.16 (m, 4H), 0.97 (d, *J* = 4.6 Hz, 21H) ppm; **^13^C‑NMR** (101 MHz, CDCl_3_): δ = 170.6, 138.6, 138.5, 138.0, 128.8, 128.4, 128.4, 128.4, 128.3, 128.1, 128.0, 127.7, 127.6, 126.8, 97.3, 78.2, 75.3, 74.2, 72.8, 71.8, 69.0, 67.4, 62.7, 36.2, 31.2, 29.2, 29.0, 28.6, 25.7, 21.1, 18.0, 17.9, 12.0 ppm; **ESI-MS**: m/z M_calcd_ for C_44_H_64_O_7_SSi = 764.4142; M_found_ = 787.4054 [M+Na]^+^.

*Step 2*

To a solution of **17** (0.784 mmol, 0.6 mg) in ACN (10 ml) and DCM (5 ml), water (100 μl) and Sc(OTf)_3_ (2.353 mmol, 1.2 mg) were added and the solution was heated to 50°C for 3 h. The reaction was quenched with pyridine (100 μl) and the solvents were removed *in vacuo*. The residue was co-evaporated with toluene and purified by silica gel column chromatography to obtain **18** in 75% yield (0.588 mmol, 0.360 g) as colorless oil. **R_f_** = 0.2 (4:1, Hex/EtOAc); **^1^H‑NMR** (400 MHz, CDCl_3_): δ = 7.48 – 6.88 (m, 15H), 5.34 (dd, *J* = 3.4, 1.8 Hz, 1H), 4.90 (d, *J* = 10.9 Hz, 1H), 4.77 – 4.65 (m, 2H), 4.61 (d, *J* = 10.8 Hz, 1H), 4.53 (d, *J* = 11.2 Hz, 1H), 3.97 (dd, *J* = 9.3, 3.3 Hz, 1H), 3.79 (qd, *J* = 11.9, 5.4 Hz, 3H), 3.71 – 3.57 (m, 4H), 3.34 (dt, *J* = 9.6, 6.5 Hz, 1H), 2.39 (t, *J* = 7.3 Hz, 2H), 2.13 (s, 3H), 1.53 (q, *J* = 7.4 Hz, 4H), 1.39 – 1.23 (m, 4H) ppm; **^13^C‑NMR** **from HSQC** (101 MHz, CDCl_3_, coupled): δ = 128.8, 128.4, 128.4, 128.4, 128.3, 128.1, 128.0, 127.7, 127.6, 126.8, 97.9, 78.1, 75.3, 74.3, 71.8, 71.7, 68.8, 67.8, 62.1, 36.2, 31.3, 29.3, 28.6, 26.0, 21.3 ppm; **ESI-MS**: m/z M_calcd_ for C_35_H_44_O_7_S = 608.2808; M_found_ = 631.2714 [M+Na]^+^.

6-(Benzylthio)-hexyl 2-*O*-acetyl-3,4-*O*-benzyl-6-hydroxy-α-D-mannopyranosyl-(1→2)-3,4,6-tri-*O*-benzyl-α-D-mannopyranosyl-(1→6)-2-*O*-acetyl-3,4-di-*O*-benzyl-α-D-mannopyranoside (**21**)

*Step 1*

A mixture of trichloroacetamidate donor **19**[2] (0.411 mmol, 0.466 g) and acceptor **18** (0.411 mmol, 0.250 g) was coevaporated three times with toluene and dried under high vacuum for 1 h. After that the reaction mixture was dissolved in a 2:1 mixture of thiophene and DCM followed by addition of molecular sieves. The reaction was stirred for 10 min at 0°C and TBSOTf (0.082 mmol, 20.0 µl) was added. After 1 h, the reaction was quenched using triethylamine, filtered and concentrated. The residue was purified using silica gel column chromatography to obtain trisaccharide **20** in 46% yield (0.189 mmol, 0.300 g) as colorless oil. **R_f_** = 0.6 (6:1, Hex/AcOEt); **^1^H‑NMR** (400 MHz, CDCl_3_): δ = 7.38 – 6.88 (m, 40H), 5.46 – 5.20 (m, 2H), 5.06 (s, 1H), 4.86 – 4.73 (m, 4H), 4.69 – 4.47 (m, 7H), 4.46 – 4.29 (m, 5H), 4.08 (s, 1H), 3.99 – 3.81 (m, 7H), 3.77 (t, *J* = 9.4 Hz, 1H), 3.69 – 3.59 (m, 6H), 3.54 – 3.42 (m, 4H), 3.30 – 3.20 (m, 1H), 2.32 (t, *J* = 7.3 Hz, 2H), 2.02 (s, 3H), 2.00 (s, 3H), 1.52 – 1.40 (m, 4H), 1.28 – 1.16 (m, 4H), 1.00 (d, *J* = 4.6 Hz, 21H) ppm; **^13^C‑NMR** (101 MHz, CDCl_3_): δ = 170.4, 170.2, 138.8, 138.6, 138.5, 138.4, 138.3, 138.1, 138.0, 137.8, 128.8, 128.5, 128.4, 128.4, 128.3, 128.2, 128.2, 127.9, 127.8, 127.8, 127.7, 127.6, 127.6, 127.5, 127.5, 127.3, 126.9, 98.9 (x2), 97.6, 79.6, 78.6, 77.9, 77.2, 75.2, 75.0, 74.9, 74.3, 73.9, 73.8, 73.3, 73.2, 73.1, 71.8, 71.7, 71.5, 70.5, 68.9, 68.9, 68.6, 67.7, 66.1, 62.5, 36.2, 31.2, 29.2, 29.0, 28.6, 25.8, 21.1, 21.0, 18.0, 17.9, 12.0 ppm; **ESI-MS**: m/z M_calcd_ for C_93_H_116_O_18_SSi = 1580.7652; M_found_ = 1603.7544 [M+Na]^+^.

*Step 2*

To a solution of trimannose **20** (0.190 mmol, 0.300 mg) in ACN (5 ml) and DCM (3 ml), water (50 μl) and Sc(OTf)_3_ (0.569 mmol, 0.280 g) were added and the solution was heated to 50 °C for 6 h. The reaction was quenched with pyridine (50 μl) and the solvents were removed *in vacuo*. The residue was co-evaporated with toluene and purified through silica gel column chromatography to obtain alcohol **21** in 70% yield (0.133 mmol, 190.0 mg) as colorless oil. **R_f_** = 0.2 (4:1, Hex/AcOEt); **^1^H‑NMR** (400 MHz, CDCl_3_): δ = 7.37 – 7.06 (m, 40H), 5.49 (t, *J* = 2.4 Hz, 1H), 5.37 – 5.31 (m, 1H), 5.00 (s, 2H), 4.90 – 4.79 (m, 3H), 4.75 – 4.56 (m, 7H), 4.52 – 4.35 (m, 5H), 4.04 (d, *J* = 2.2 Hz, 1H), 3.99 – 3.80 (m, 6H), 3.79 – 3.47 (m, 12H), 3.32 (dd, *J* = 9.5, 6.5 Hz, 1H), 2.38 (t, *J* = 7.3 Hz, 2H), 2.10 (s, 3H), 2.09 (s, 3H), 1.51 (dt, *J* = 13.9, 7.0 Hz, 4H), 1.37 – 1.28 (m, 4H) ppm; **ESI-MS**: m/z M_calcd_ for C_84_H_96_O_18_S = 1424.6317; M_found_ = 1447.6229 [M+Na]^+^.

*Triethylammonium 2-O-acetyl-3,4-O-benzyl-6-O-(2-N-benzyloxycarbonyl)aminoethyl-phosphonato-α-D-mannopyranosyl-(1→2)-3,4,6-tri-O-benzyl-α-D-mannopyranosyl-(1→6)-1-O-(6-thiobenzyl)hexyl-2-O-acetyl-3,4-O-benzyl-α-D-mannopyranose* (**D**)

*Step 1*

Alcohol **21** (0.088 mmol, 0.125 g) and 2-amino-(benzyloxy-carbonyl)-H-phosphonate (0.438 mmol, 0.105 g) were co evaporated three times with pyridine. The residue was dissolved in pyridine (2 ml) and PivCl (0.263 mmol, 33.0 μl) was added dropwise. The solution was stirred for 2 h at room temperature before water (15 μl) and iodine (0.316 mmol, 80.0 mg) were added. The red solution was stirred for 1 h and quenched with sat. Na_2_S_3_O_3_ solution. The reaction mixture was diluted with chloroform and dried over Na_2_SO_4_. The reaction mixture was filtered and concentrated *in* *vacuo*. The crude residue was purified through flash column chromatography on deactivated (1% TEA in CHCl_3_) silica gel using methanol and chloroform an eluent to give phosphate **22** as yellow oil in 85% yield (0.075 mmol, 125.0 mg). **R_f_** = 0.4 (10% MeOH in DCM); **^1^H-NMR** (600 MHz, CDCl_3_): δ = 7.37 – 7.07 (m, 45H), 6.65 (t, *J* = 5.0 Hz, 1H), 5.50 (t, *J* = 2.1 Hz, 1H), 5.37 (dd, *J* = 3.4, 1.8 Hz, 1H), 5.09 – 5.00 (m, 3H), 4.94 (d, *J* = 2.0 Hz, 1H), 4.90 – 4.80 (m, 4H), 4.79 – 4.67 (m, 3H), 4.64 – 4.53 (m, 4H), 4.52 – 4.42 (m, 3H), 4.37 (dd, *J* = 11.7, 5.4 Hz, 2H), 4.27 (dt, *J* = 11.6, 3.8 Hz, 1H), 4.18 – 4.11 (m, 1H), 4.09 (t, *J* = 2.3 Hz, 1H), 4.02 – 3.86 (m, 7H), 3.82 (t, *J* = 9.6 Hz, 1H), 3.79 – 3.74 (m, 2H), 3.74 – 3.69 (m, 1H), 3.68 (s, 2H), 3.61 (dt, *J* = 9.8, 6.3 Hz, 2H), 3.56 (dd, *J* = 11.2, 4.7 Hz, 1H), 3.52 – 3.48 (m, 1H), 3.40 – 3.32 (m, 3H), 2.39 (t, *J* = 7.4 Hz, 2H), 2.11 (s, 3H), 2.07 (s, 3H), 1.57 – 1.48 (m, 4H), 1.37 – 1.23 (m, 4H) ppm; **^13^C‑NMR** (151 MHz, CDCl_3_): δ = 170.4, 170.0, 156.6, 138.9, 138.6, 138.6, 138.5, 138.2, 138.1, 137.9, 137.0, 128.8, 128.4, 128.4, 128.3, 128.3, 128.2, 128.2, 128.2, 128.1, 128.0, 127.9, 127.8, 127.7, 127.7, 127.6, 127.5, 127.4, 127.4, 127.4, 127.3, 126.8, 99.5, 98.8, 97.6, 79.3, 78.6, 77.9, 74.9, 74.9, 74.6, 74.4, 74.0, 73.9, 73.1, 71.8, 71.8, 71.7, 71.6, 71.6, 70.6, 69.0, 68.9, 68.7, 67.8, 66.3, 66.2, 64.3, 64.3, 64.2, 42.5, 36.3, 31.3, 29.3, 29.1, 28.7, 25.8, 21.1, 21.1 ppm; **^31^P‑NMR** (243 MHz, CDCl_3_): δ = 1.29 ppm; **ESI-MS**: m/z M_calcd_ for C_100_H_121_N_2_O_22_PS = 1764.7869; M_found_ = 1783.8053 [M+H_3_O]^+^.

*Step 2*

Phosphate **22** (0.030 mmol, 50.0 mg) was dissolved in THF (2 mL) and *tert-*butanol (2 drops). This solution was added to approximately 10 ml ammonia which was condensed at -78°C. Small fresh cut pieces of sodium were added till a dark blue color was established. The reaction was stirred for 35 min at -78°C. The reaction was quenched with MeOH (2 mL) and ammonia was blown off using a stream of nitrogen. The solution was adjusted with concentrated acetic acid to pH 7. Water was removed by freeze drying and the residue was purified using a Sephadex super fine G-25 (GE Healthcare) column (1 cmx20 cm) to yield compound **D** as a white solid mixture of free thiol and disulphide in 35% yield (0.011 mmol, 19.0 mg). **^1^H‑NMR** (400 MHz, D_2_O): δ = 4.97 (s, 1.5H), 4.89 (s, 1.5H), 4.70 (s, 1.5H), 4.03 – 3.93 (m, 8H), 3.85 – 3.71 (m, 10H), 3.67 – 3.53 (m, 12H), 3.42 (dt, *J* = 10.9, 6.5 Hz, 2H), 3.14 (t, *J* = 4.9 Hz, 3H), 2.64 (t, *J* = 7.2 Hz, 1H), 2.41 (t, *J* = 7.1 Hz, 2H), 1.49 (q, *J* = 6.8 Hz, 6H), 1.32 – 1.12 (m, 6H) ppm; **^13^C‑NMR** (101 MHz, D_2_O): δ = 102.3, 99.8, 97.9, 78.9, 72.7, 72.0, 71.9, 71.1, 70.7, 70.1, 70.0, 69.8, 67.87, 66.8, 66.7, 66.2, 66.1, 64.7, 61.8, 60.8, 40.0, 32.8, 28.3, 27.1, 24.8, 23.6 ppm; **^31^P‑NMR** (162 MHz, D_2_O): δ = 0.25 ppm; **ESI-MS**: m/z M_calcd_ for C_26_H_50_NO_19_PS = 743.2435; M_found_ = 766.2354 [M+Na]^+^.

*VSG117 CTD peptide (KGKLEDTCKKESNCKWENNA)* (**F**)

In a fritted reaction vessel 200 mg trityl-ChemMatrix® resin (substitution grade 0.62 mmol/g) were swollen in anhydrous DCM for 2 hours. After DCM was drained, a solution of 10% AcBr in anhydrous DCM (14.00 ml) was added to the resin and the slurry was shaken for 4 hours. The resin was washed numerous times with anhydrous DCM and a solution of 350 mg Fmoc-Thr(OtBu)-OH and 400 μ l DIPEA in 10 ml anhydrous DCM was added to the resin and shaken for 16 hours. The resin was washed neatly with DCM and the efficiency of the coupling was determined by Fmoc quantification. The resin was capped using a mixture of methanol, DIPEA and DCM (2:1:17) for 10 minutes. Coupling reagents used were DIC and Oxyma. The coupling reagents were prepared as solutions in DMF with 1 M Oxyma (with 0.1 M DIPEA) and 0.5 M DIC. Amino acids were added as 0.2 M solutions in DMF. All amino acids were coupled twice in five-fold excess. The temperature during coupling is 50°C and the coupling time is 10 minutes, except arginine. Arginine was carried out at room temperature for 20 minutes and the second coupling was at 50°C for 10 minutes. For all amino acids Fmoc was removed three times using 20% piperidine in DMF without microwave for 5 minutes. TFA/TIPS/water (190:5:5/v:v:v) was added and the resin (100 mg when synthesis was started) was shaken for 3 hours. The cleavage solution was collected and the resin was washed with another 8 ml TFA. The solution was concentrated under nitrogen and crushed out with ice cold diethyl ether. The precipitate was centrifuged and washed two more times with ice cold diethyl ether. The resulting peptide was dried, dissolved, lyophilized and purified over RP-HPLC. MALDI: M_calcd_ for oxidized peptide = 2325.584; M_found_ = 2358.070


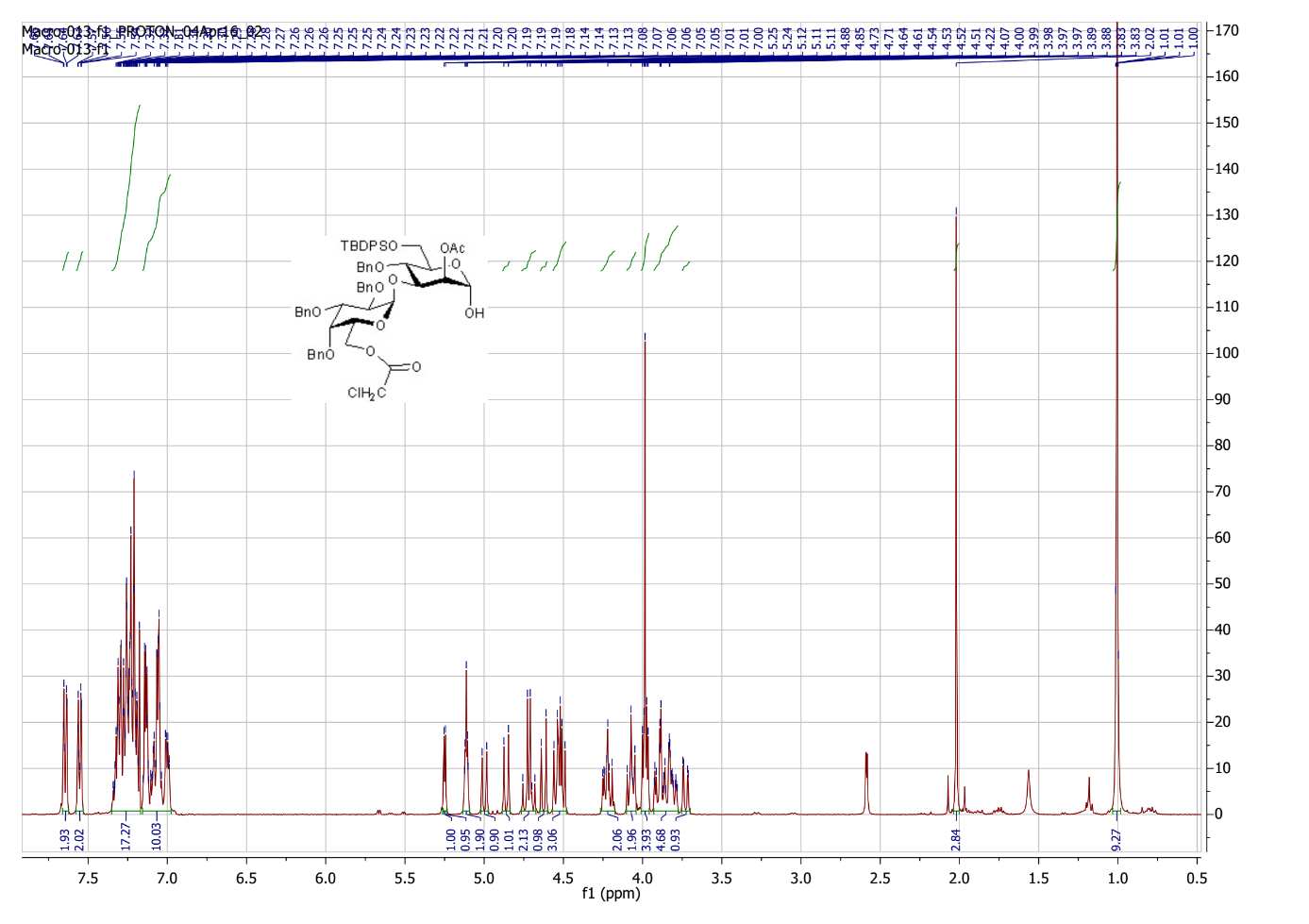

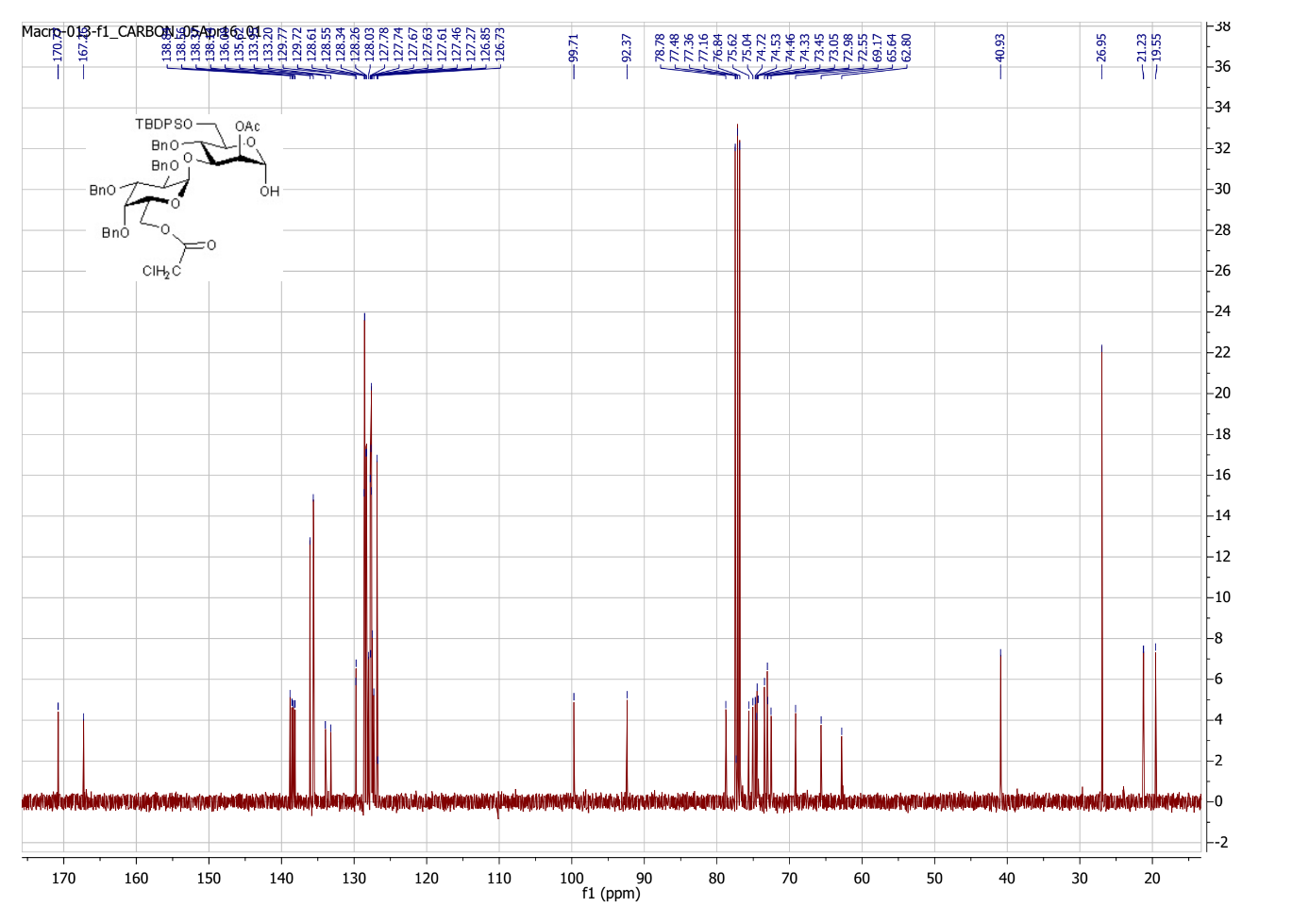


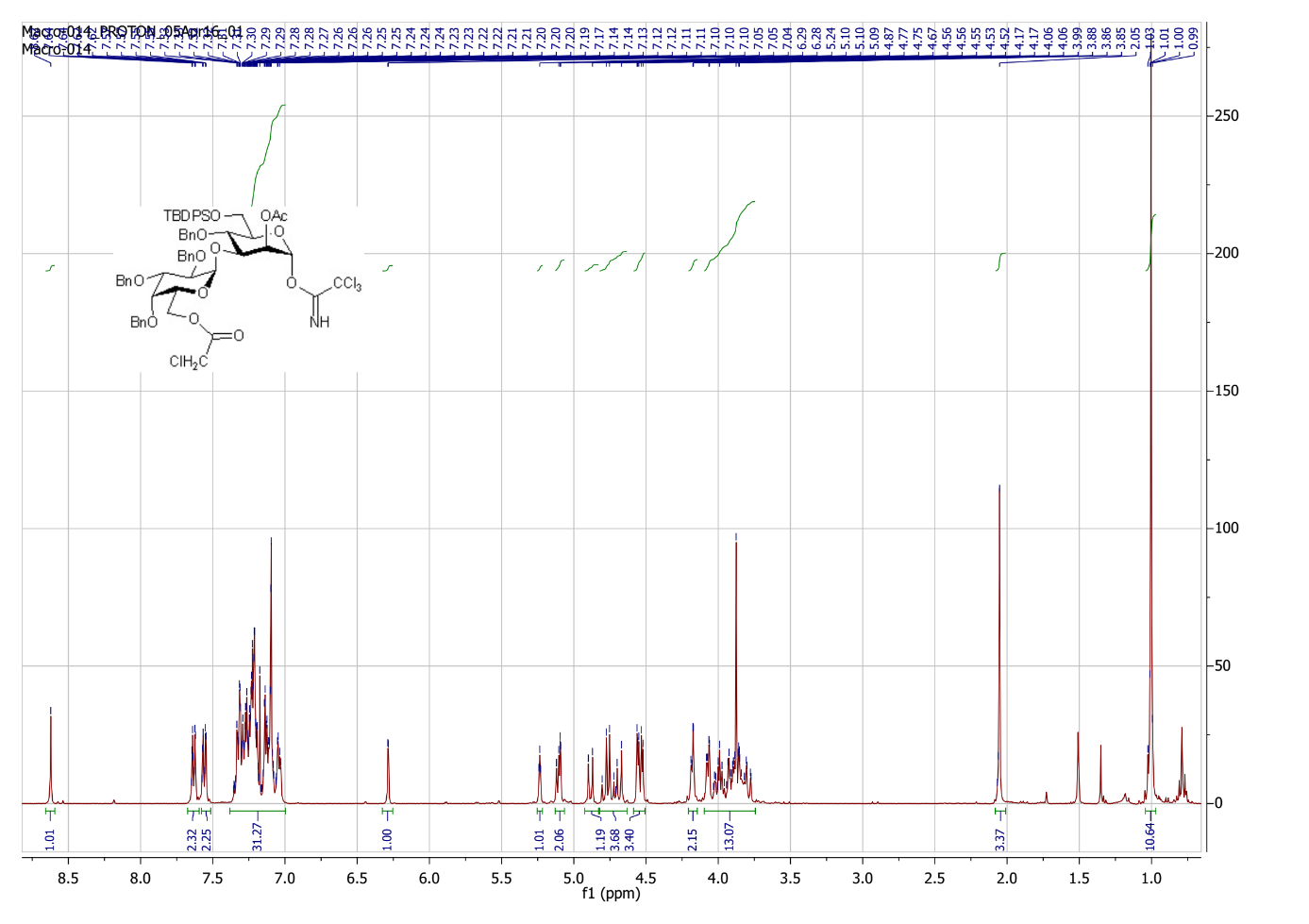


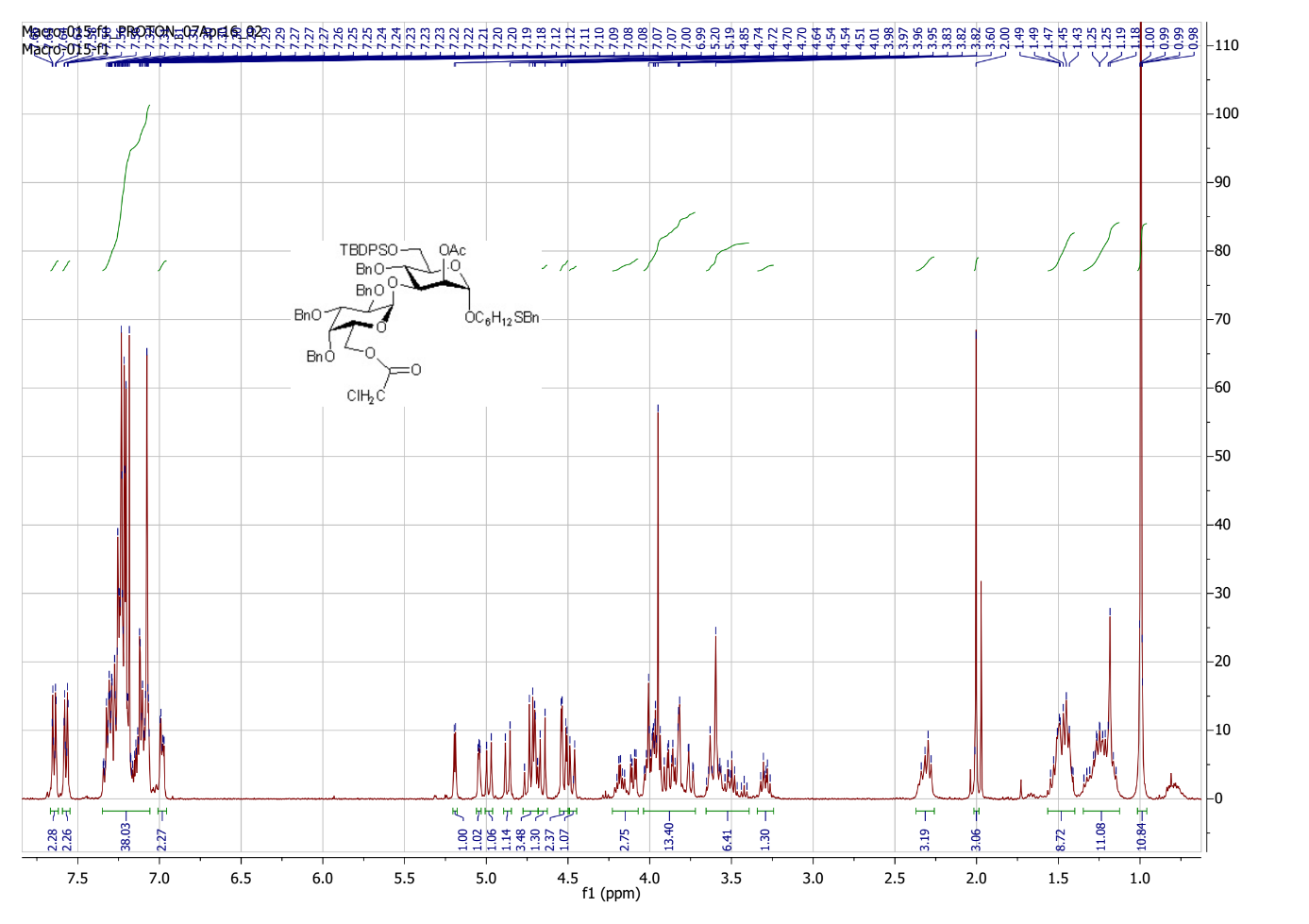


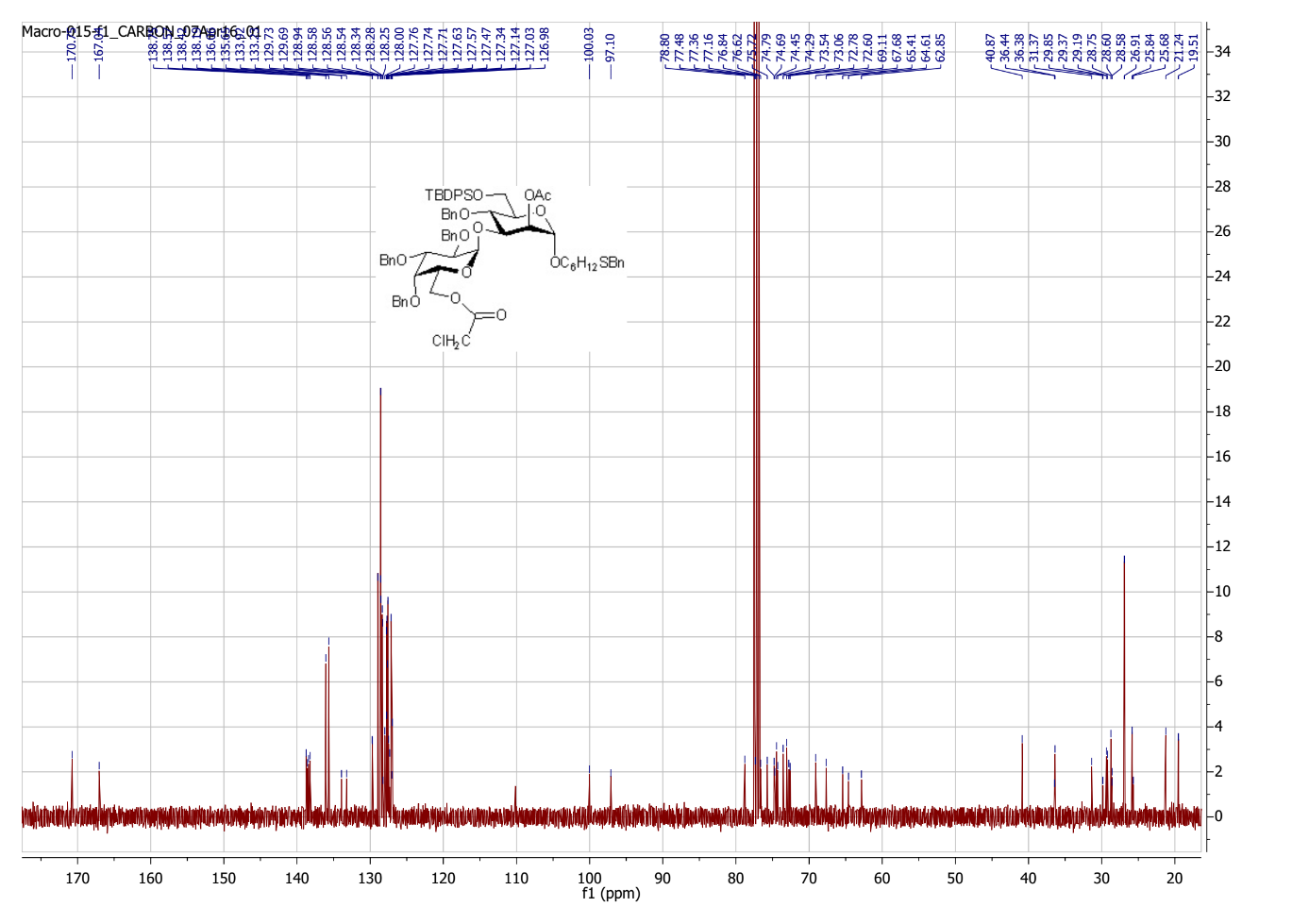


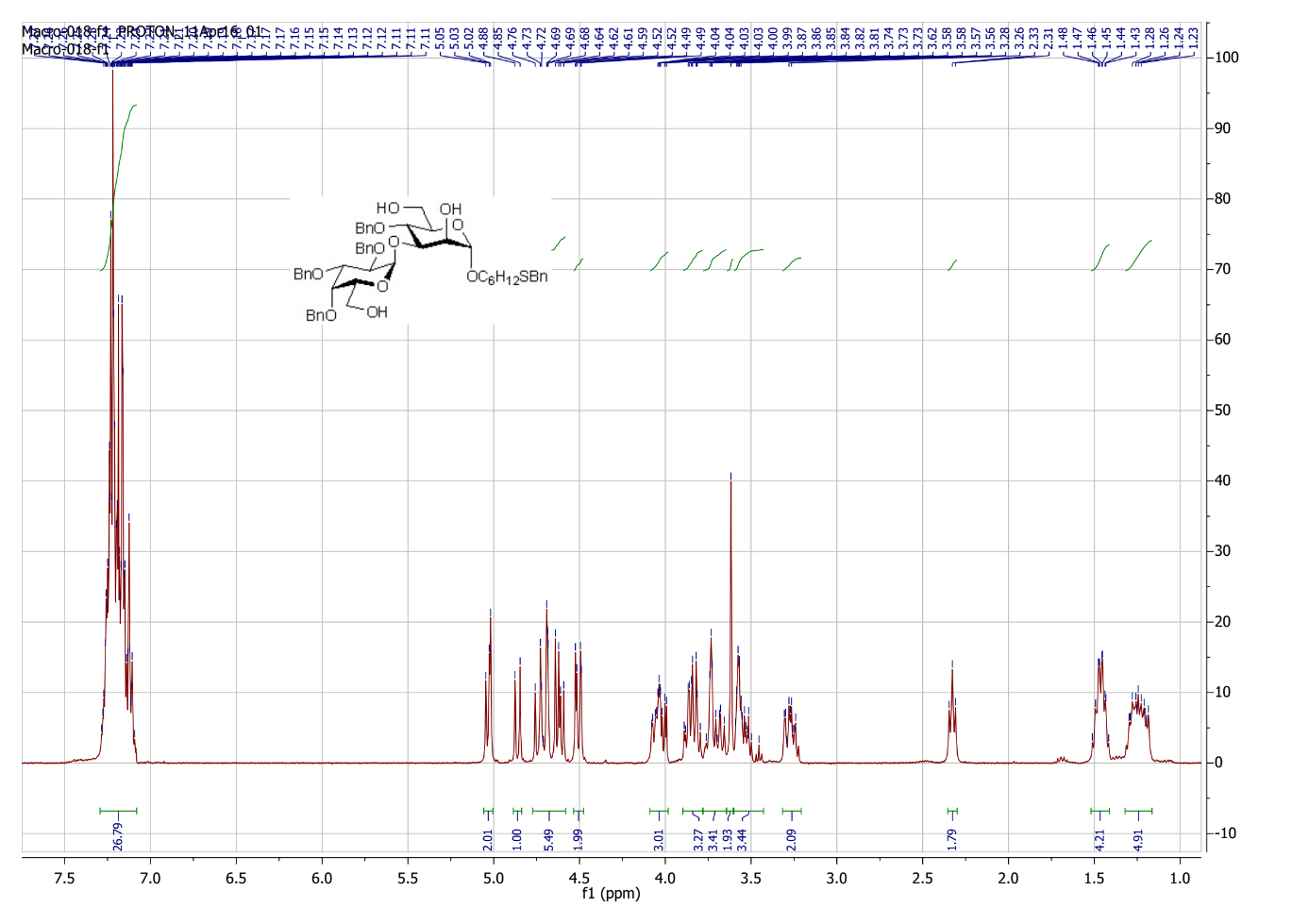


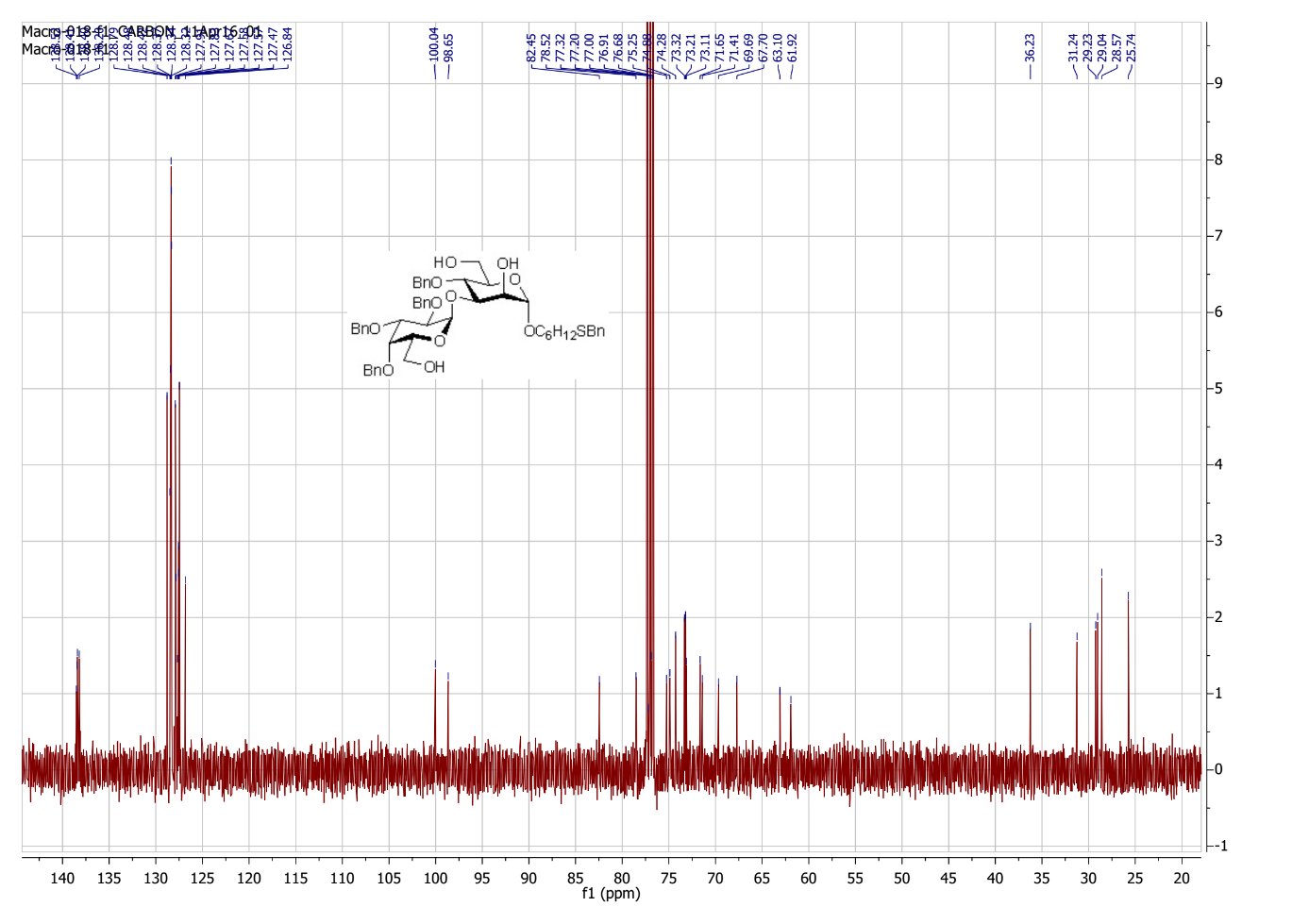


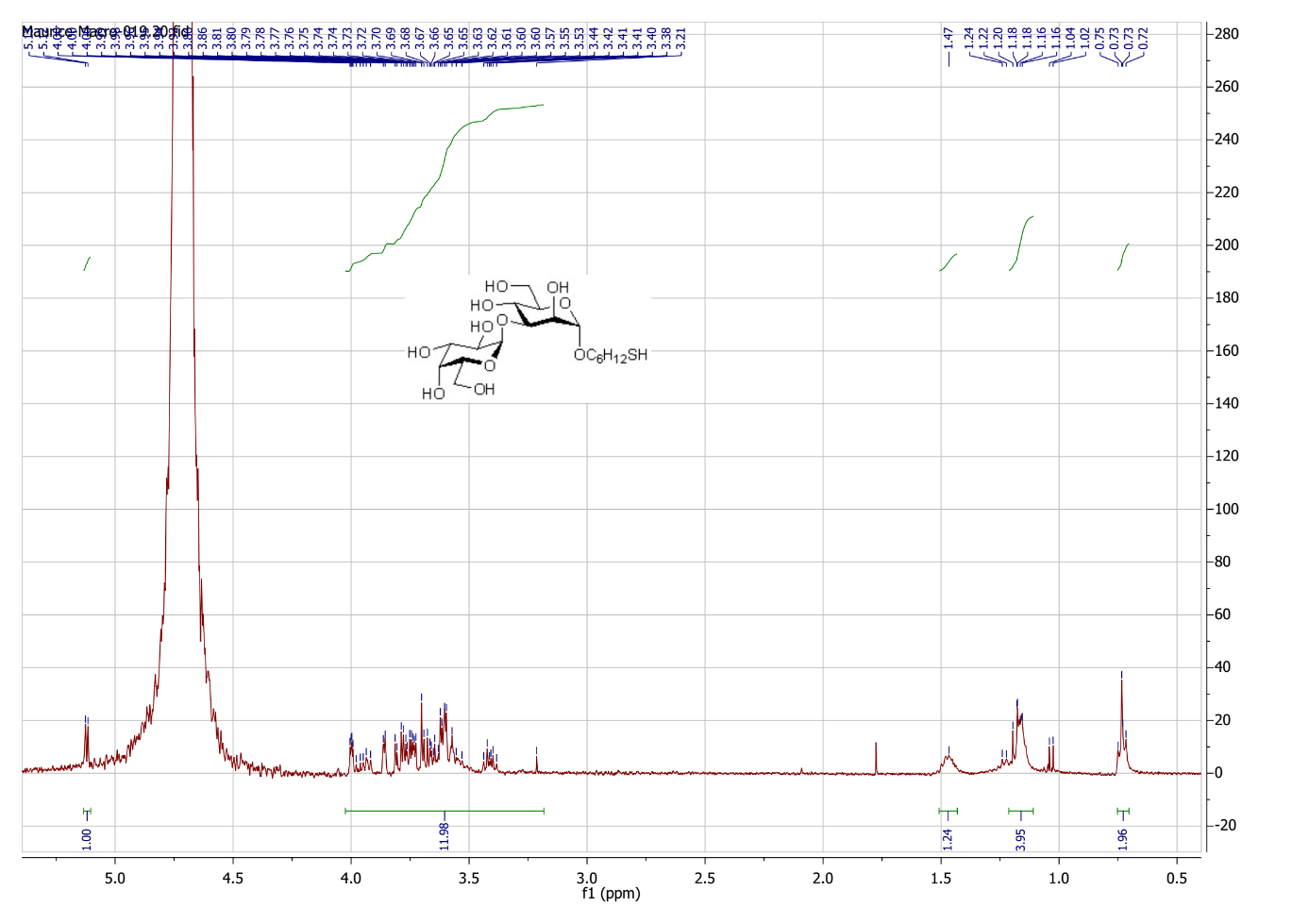


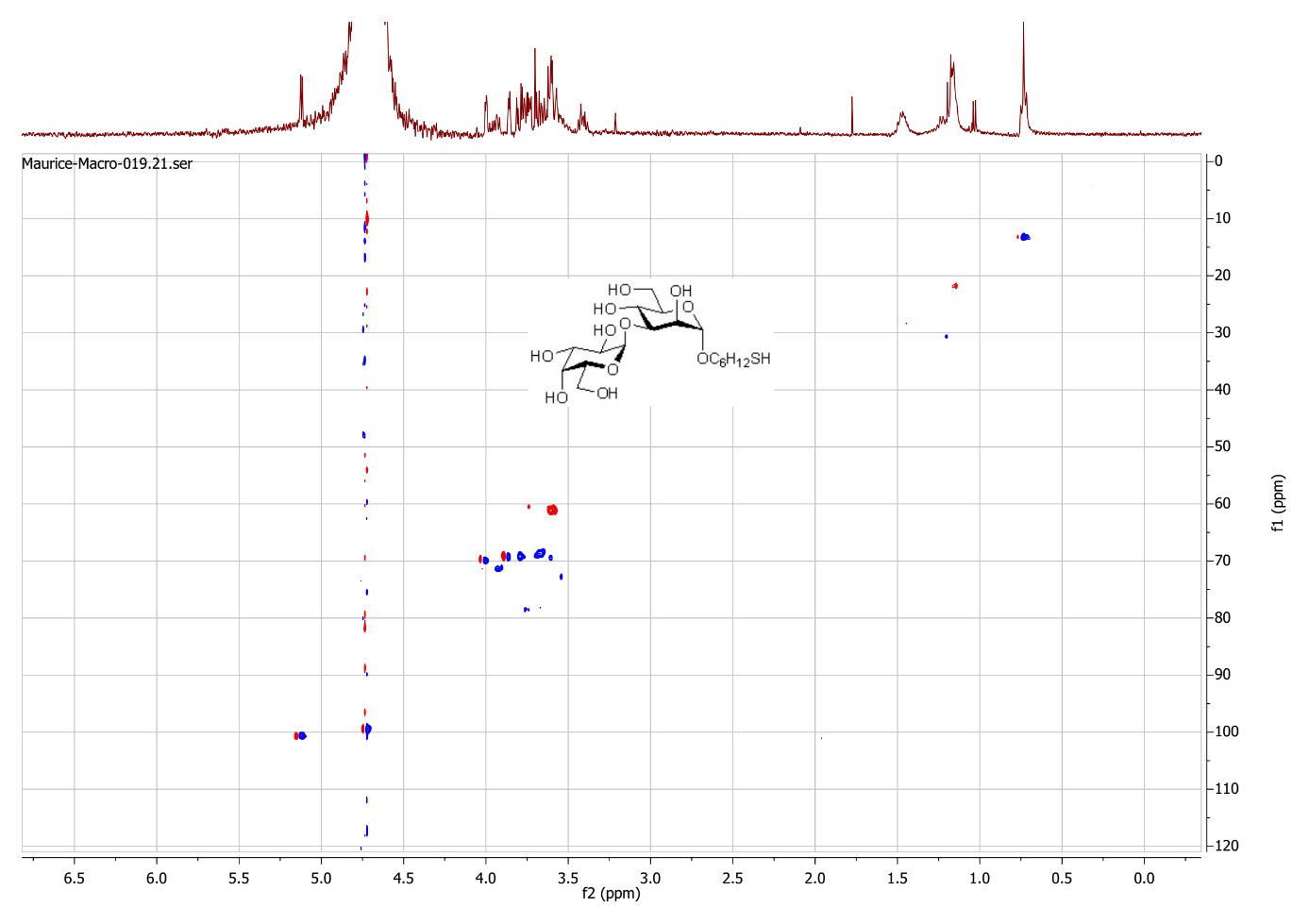


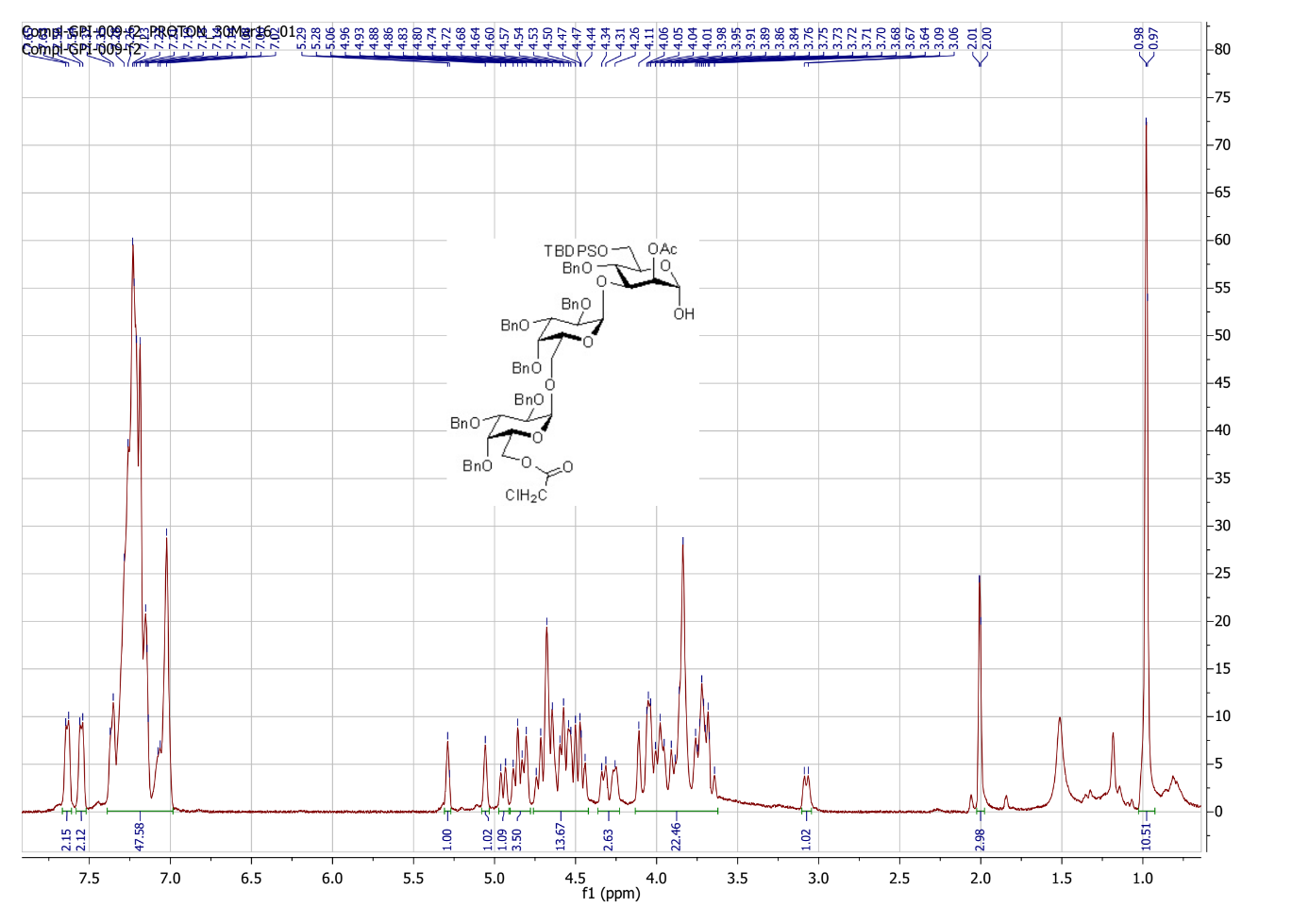


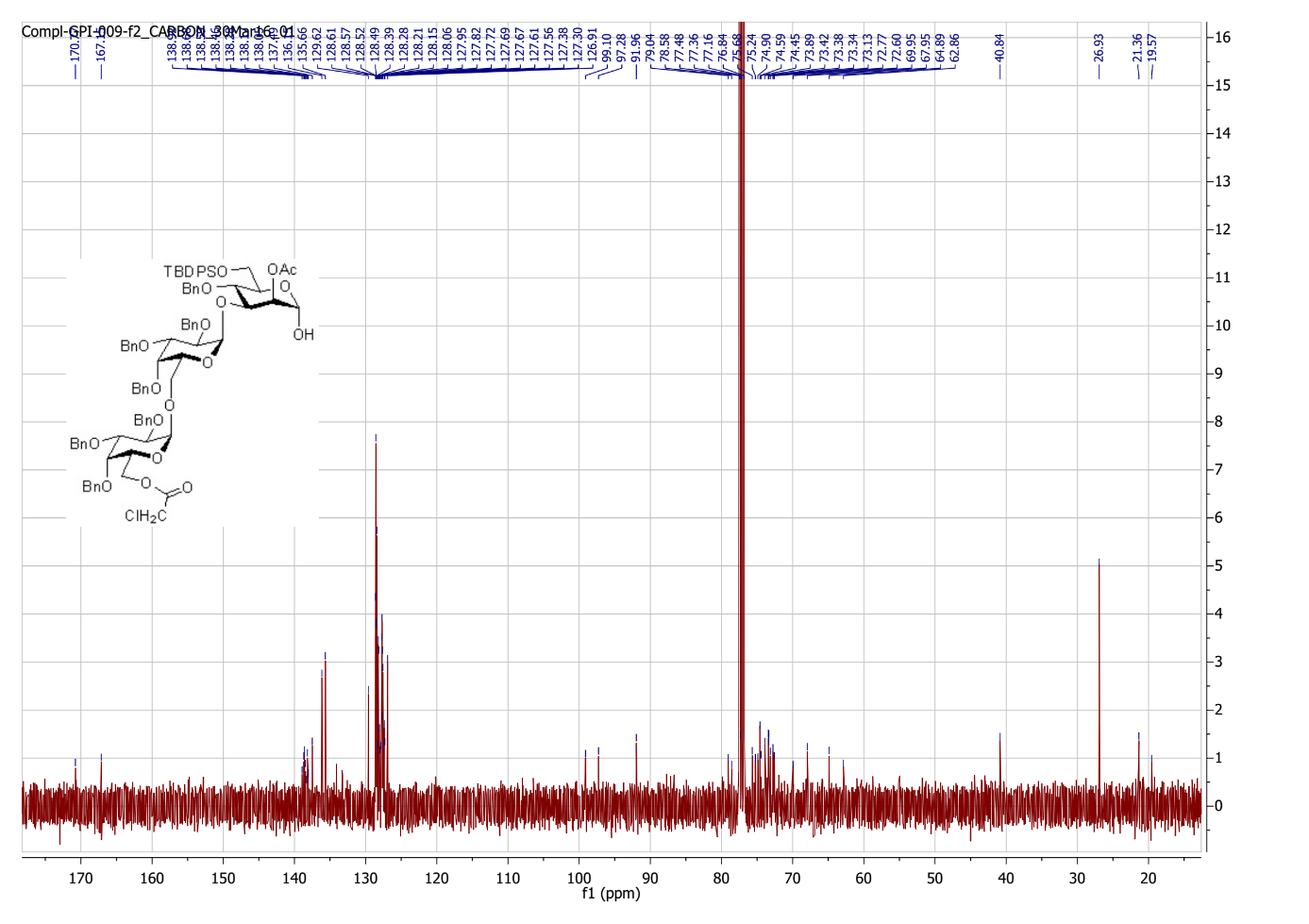


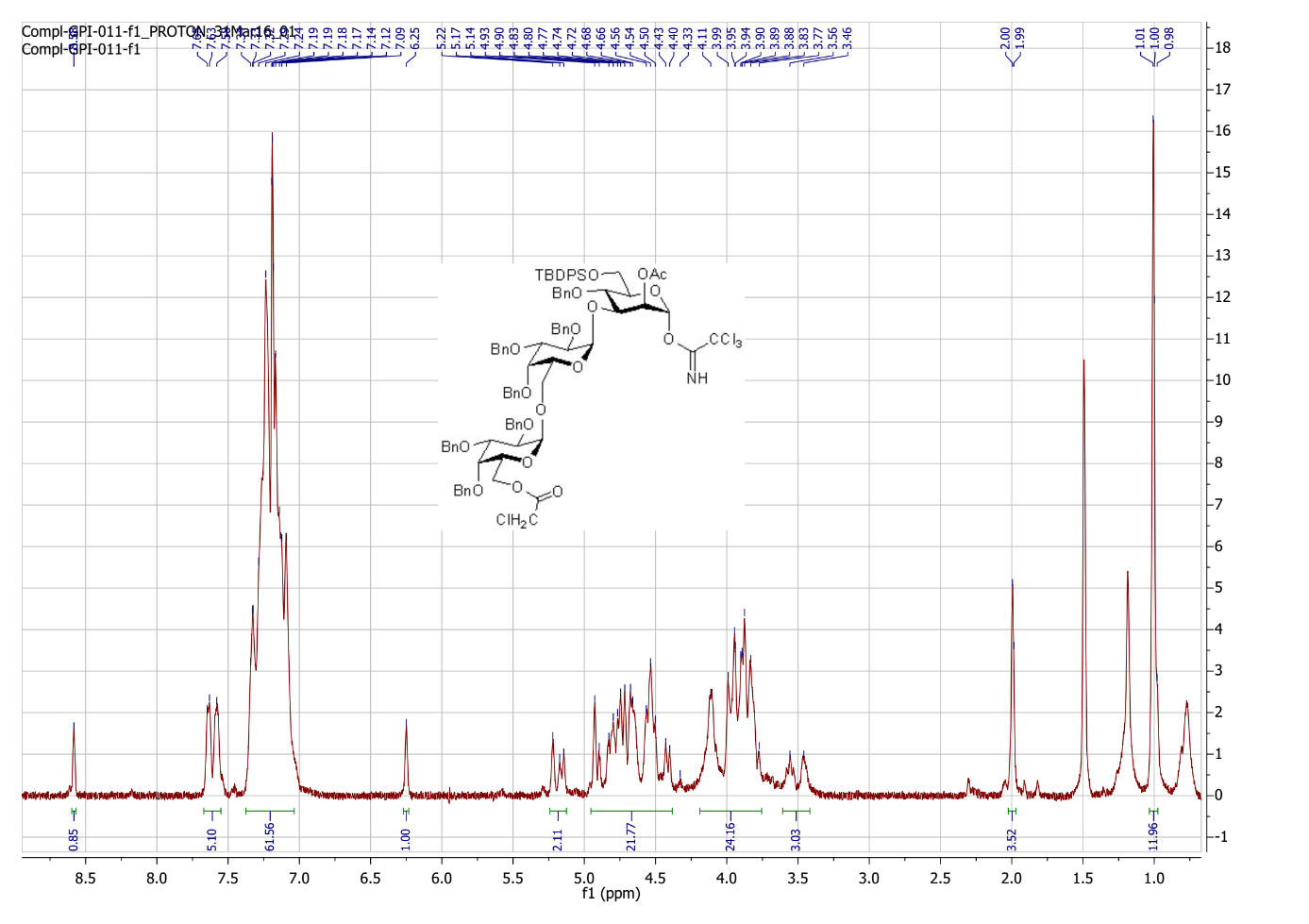


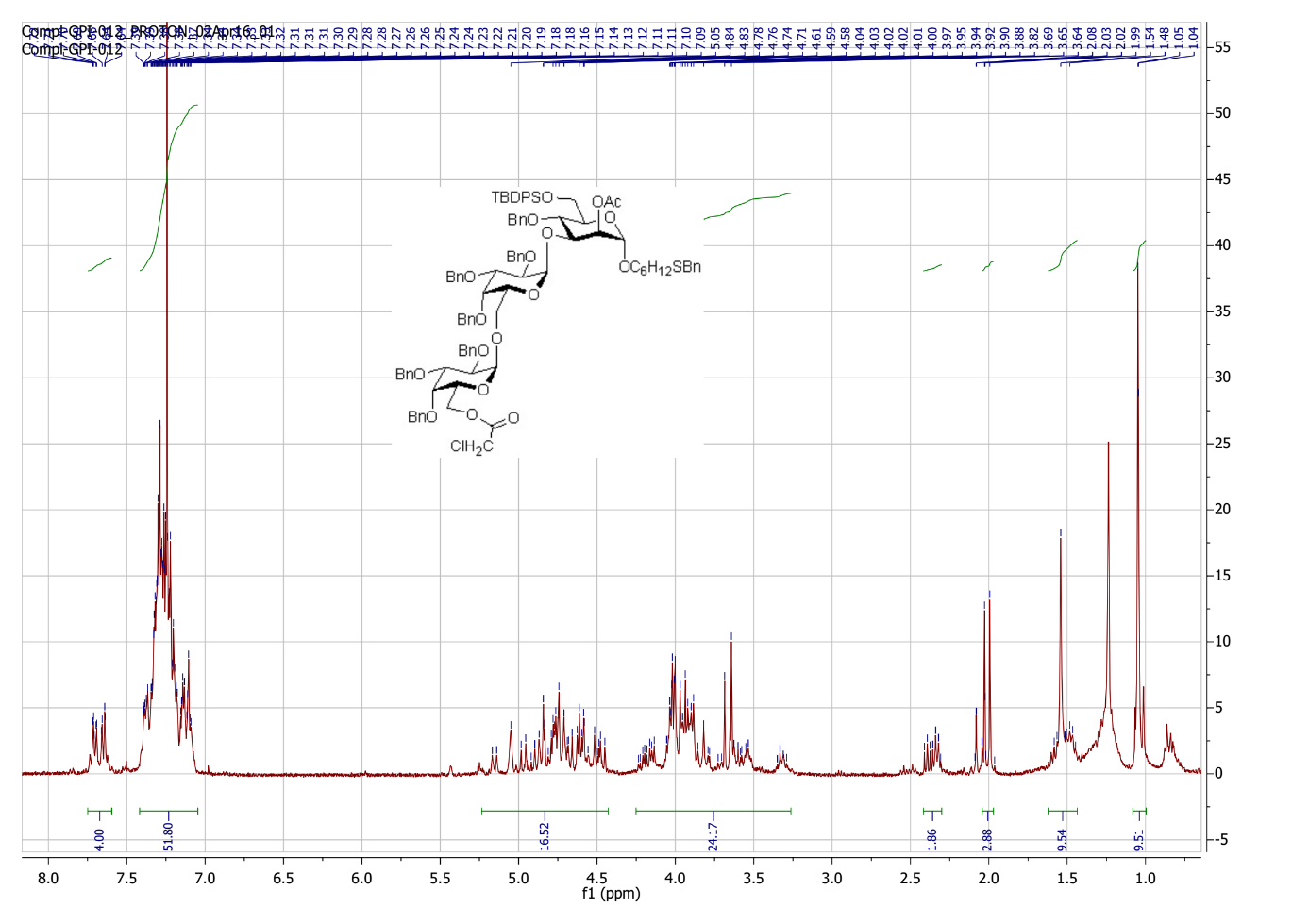


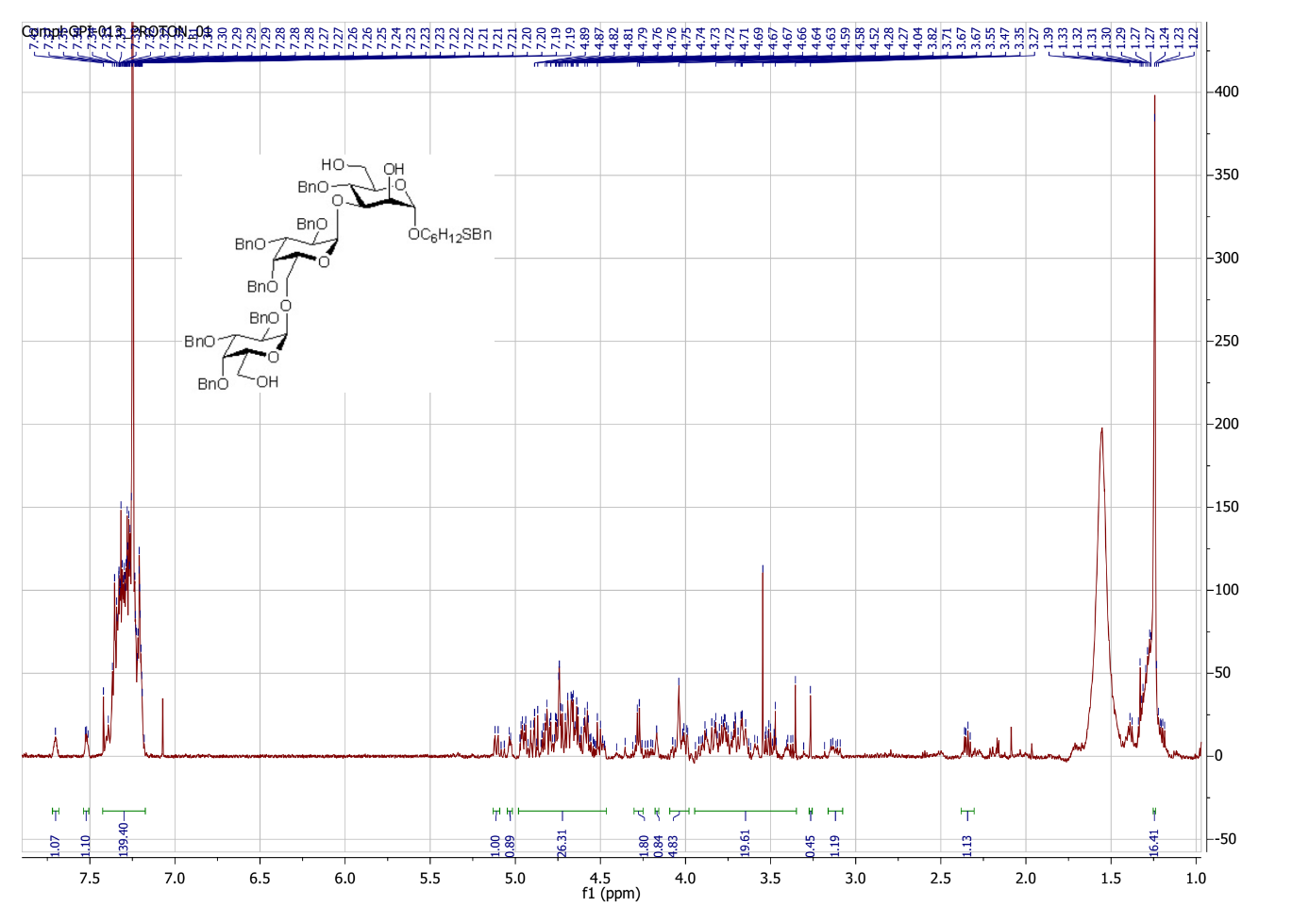


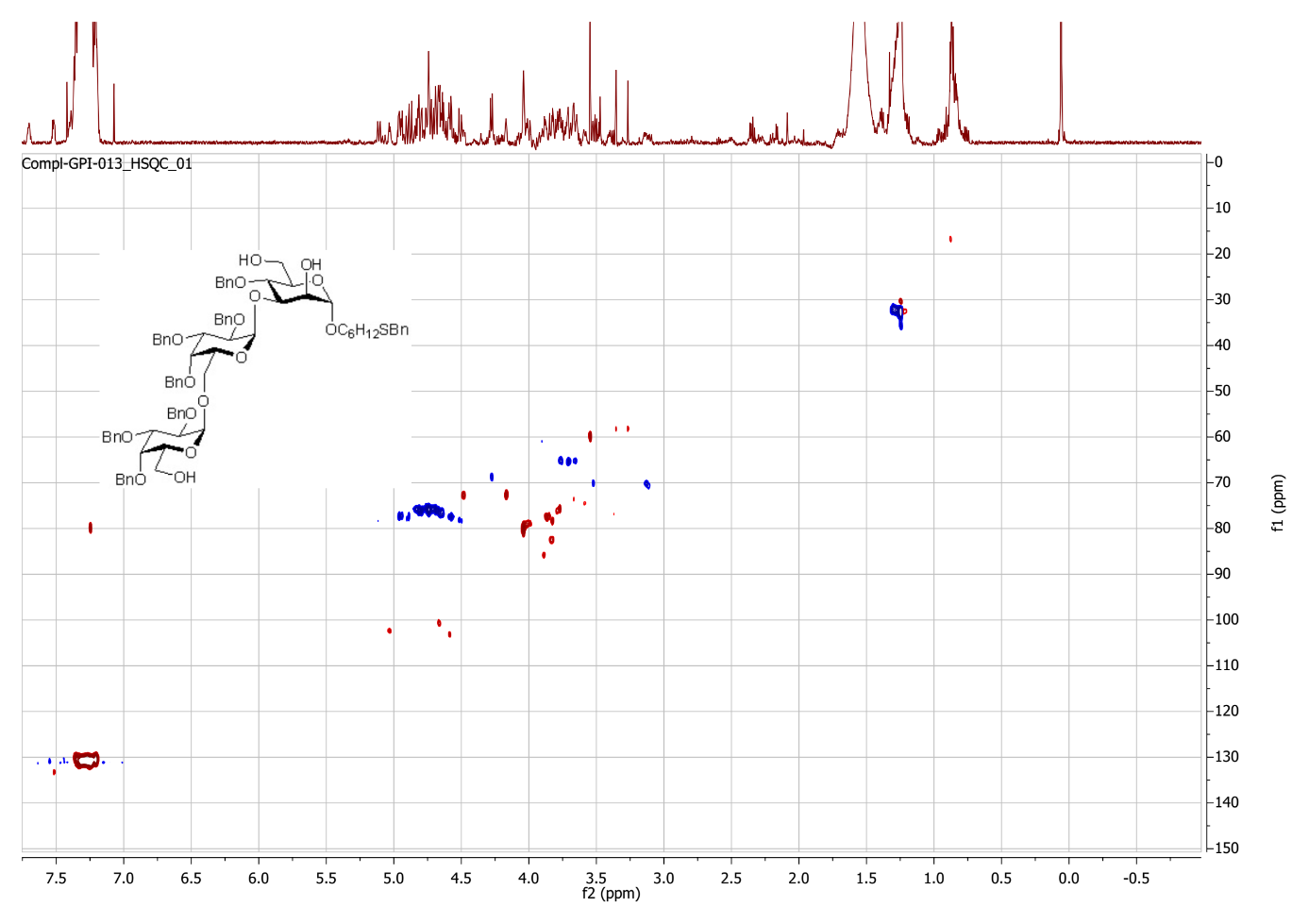


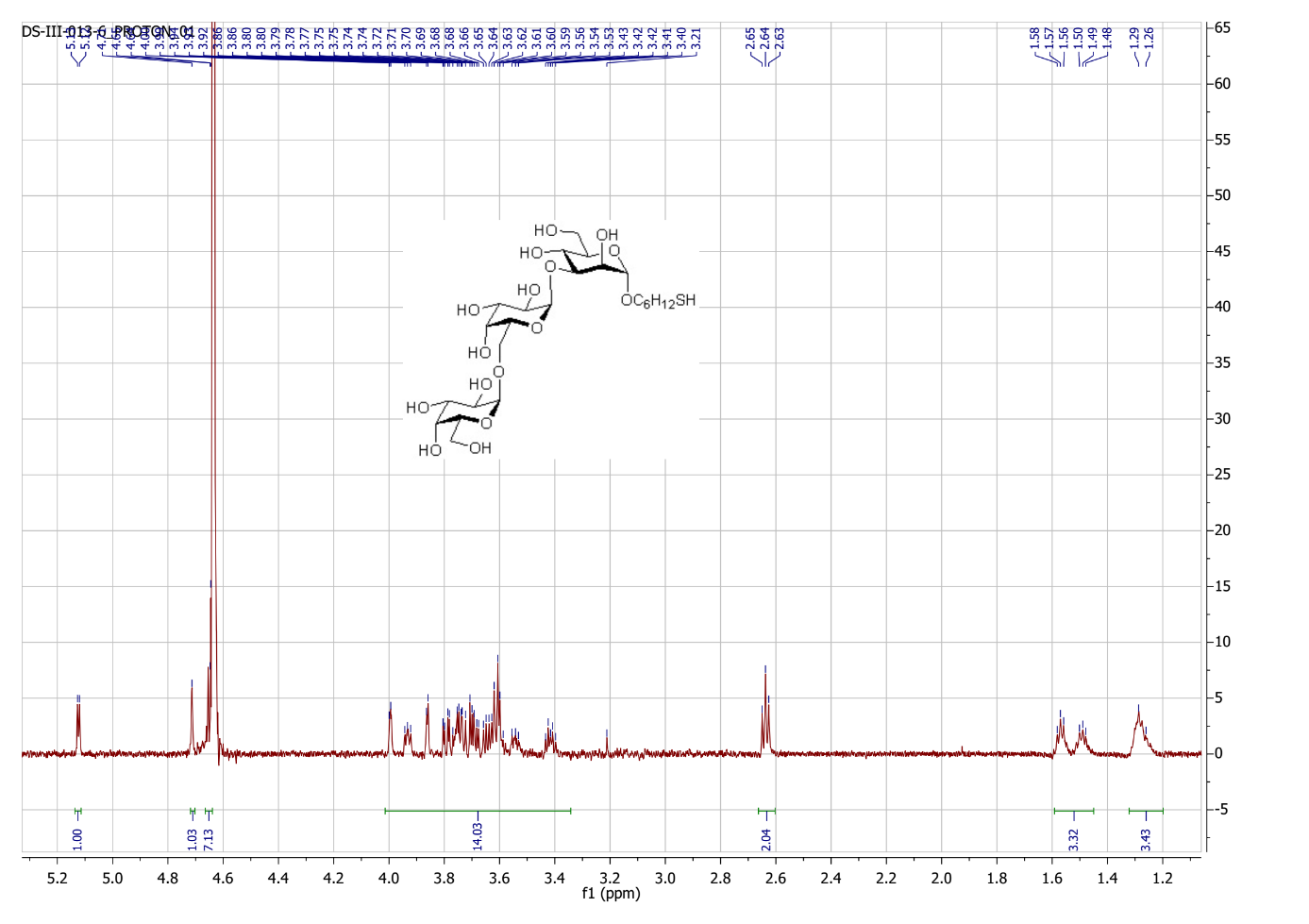


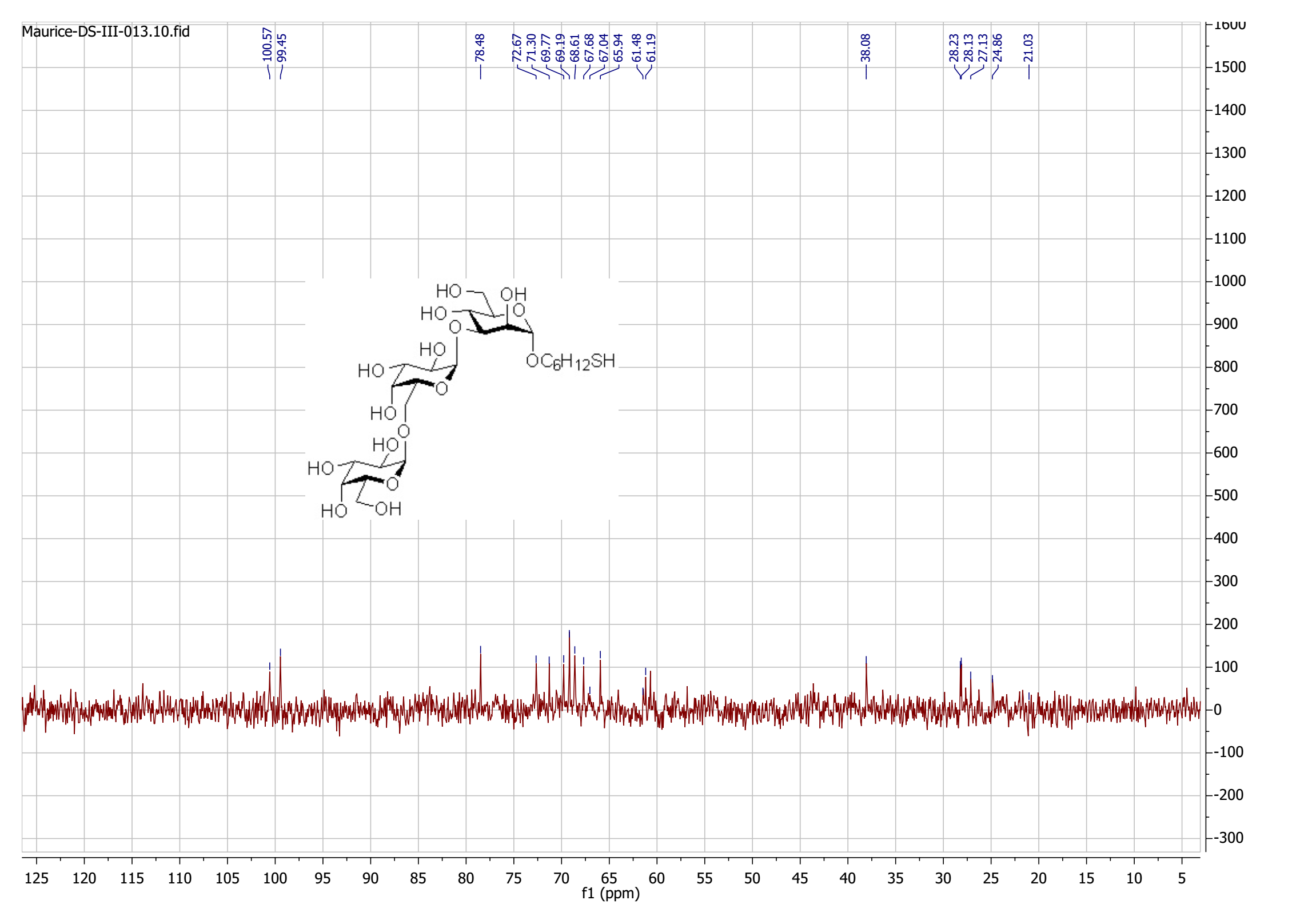


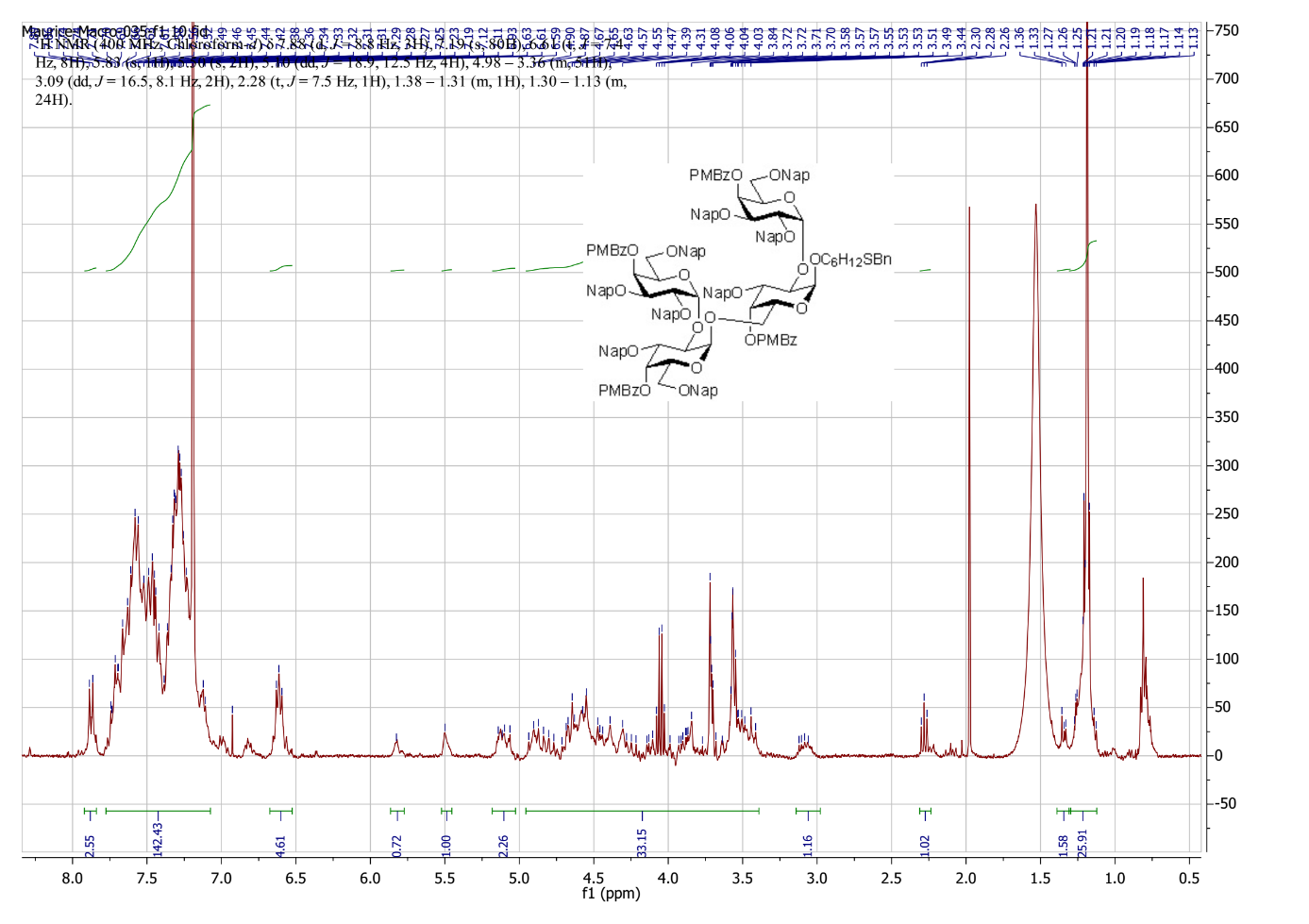


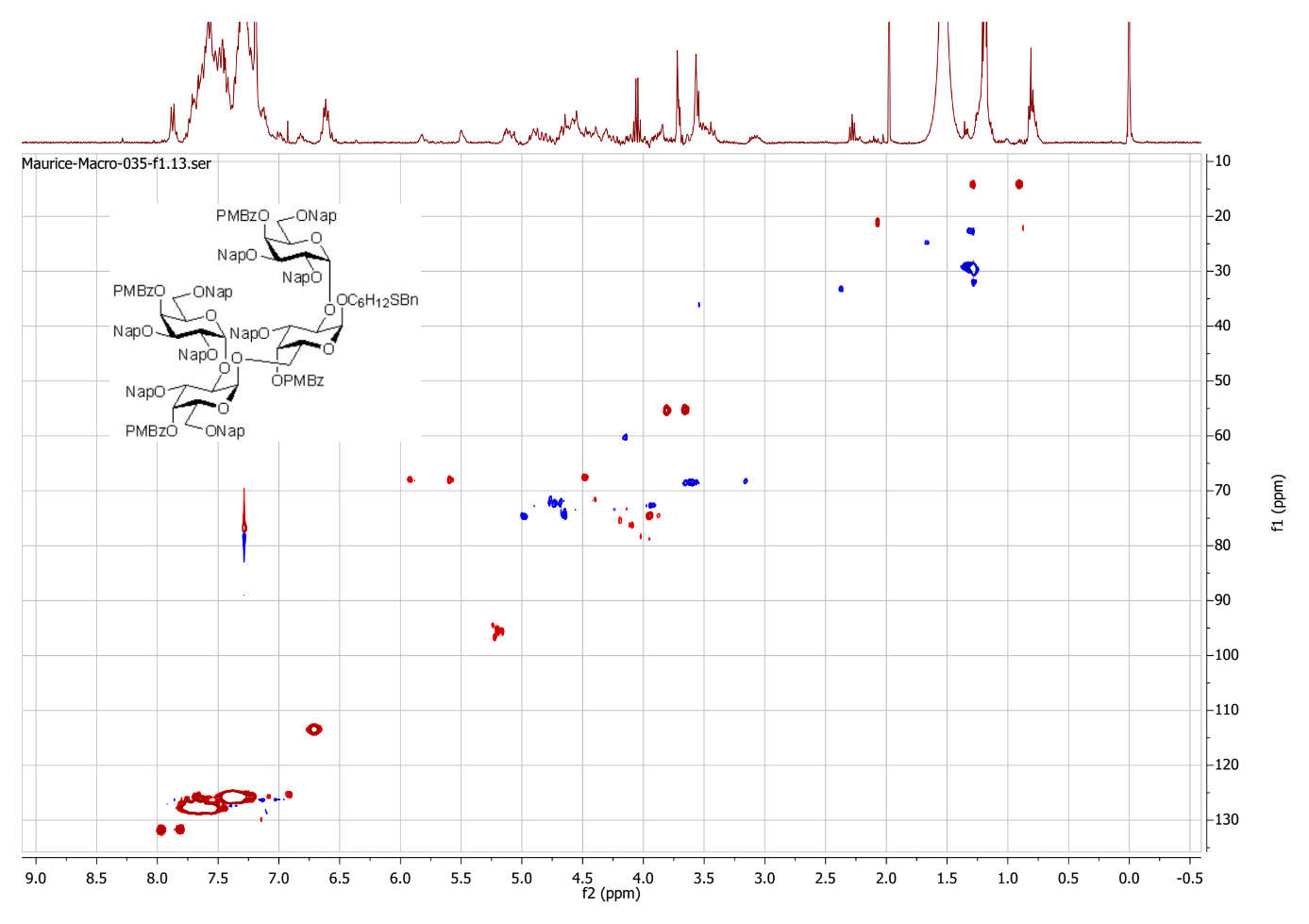


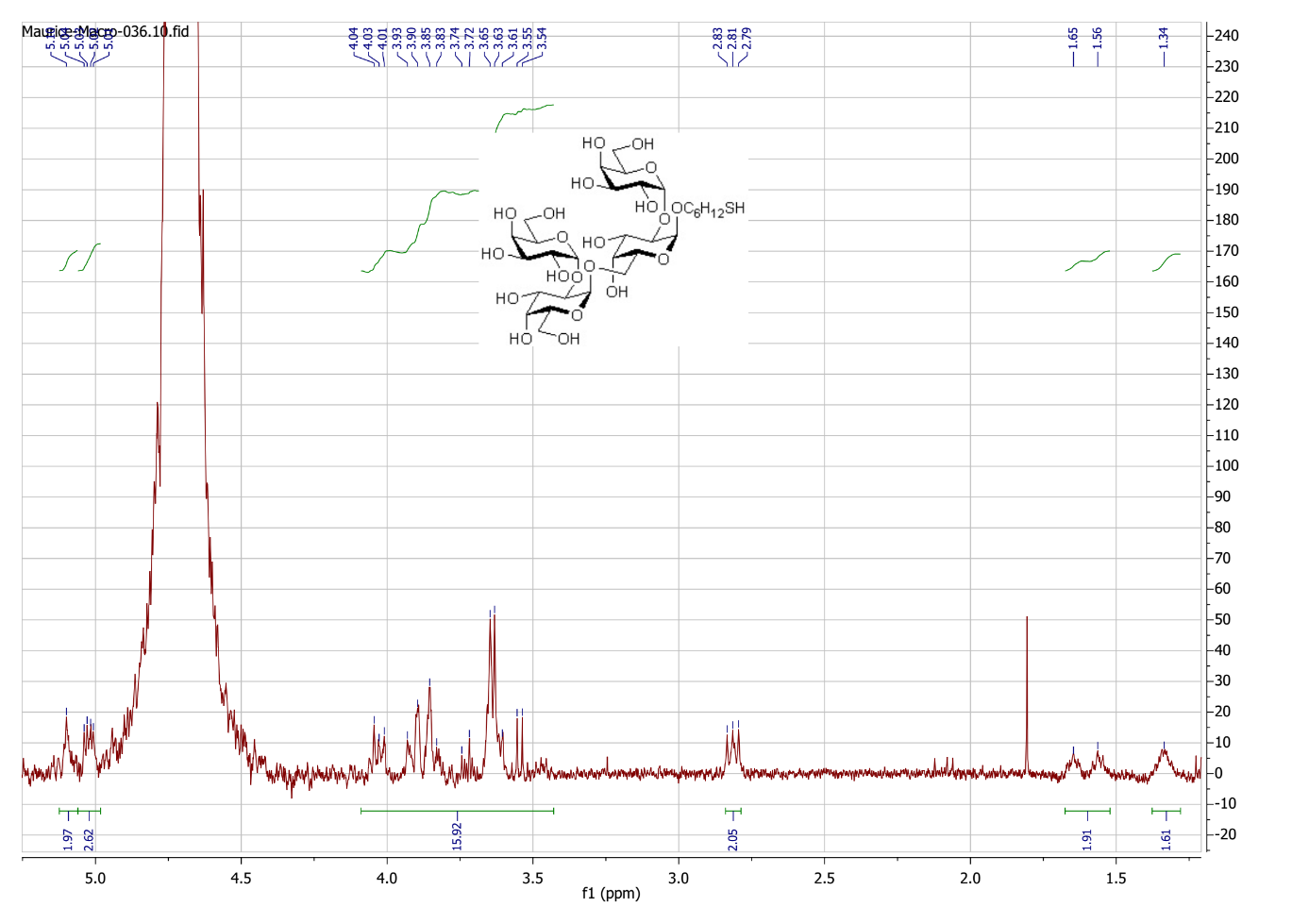


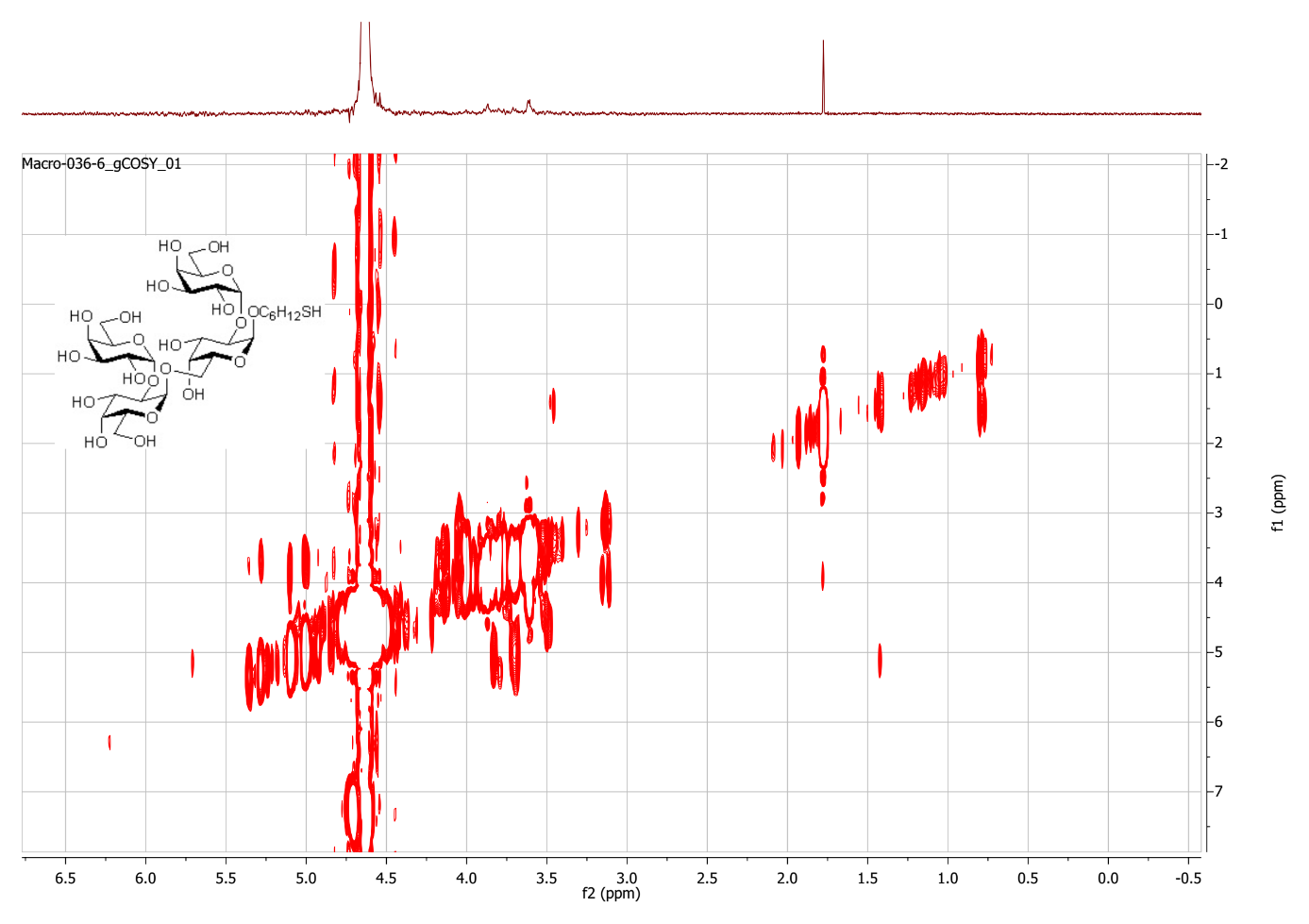


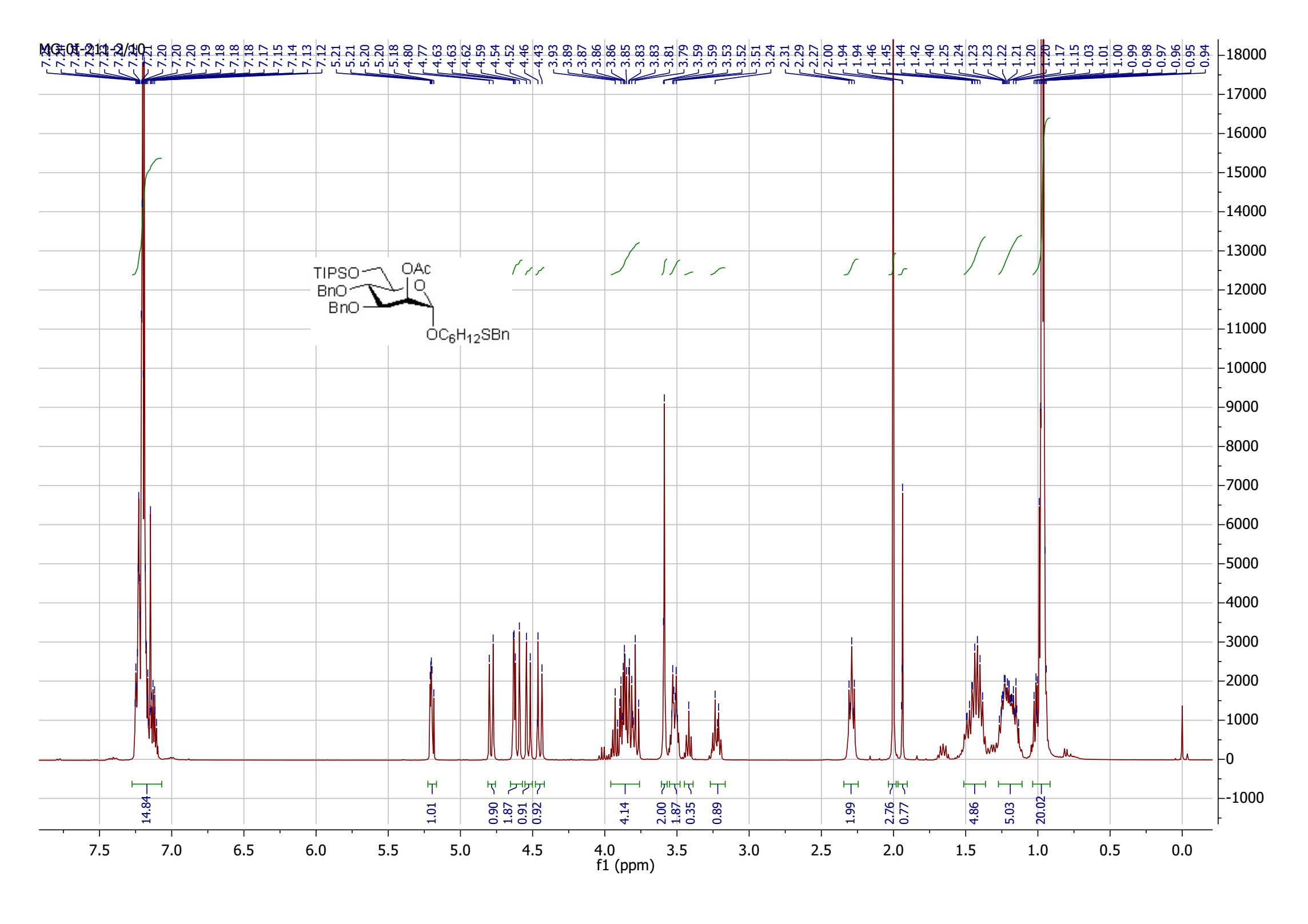


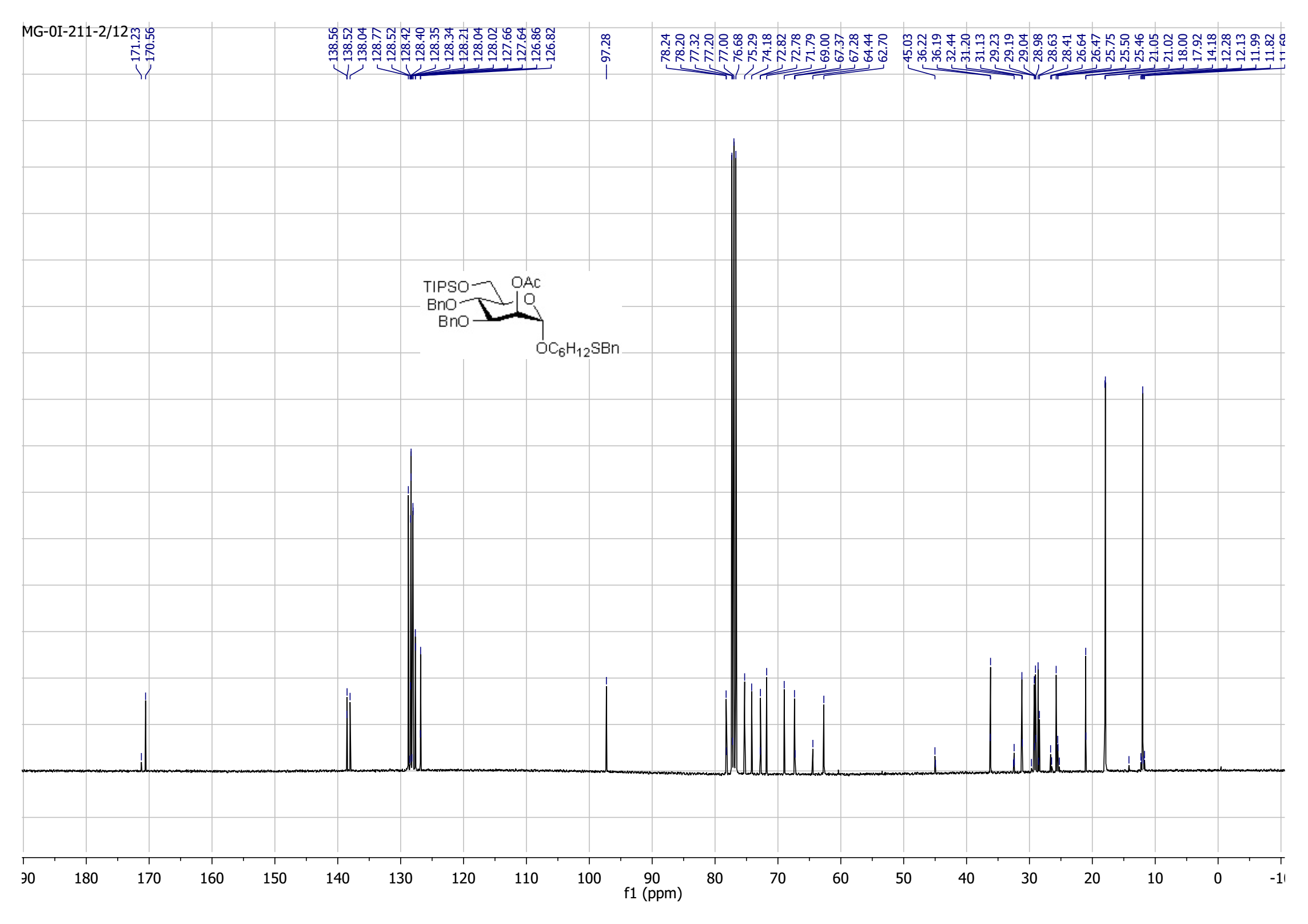


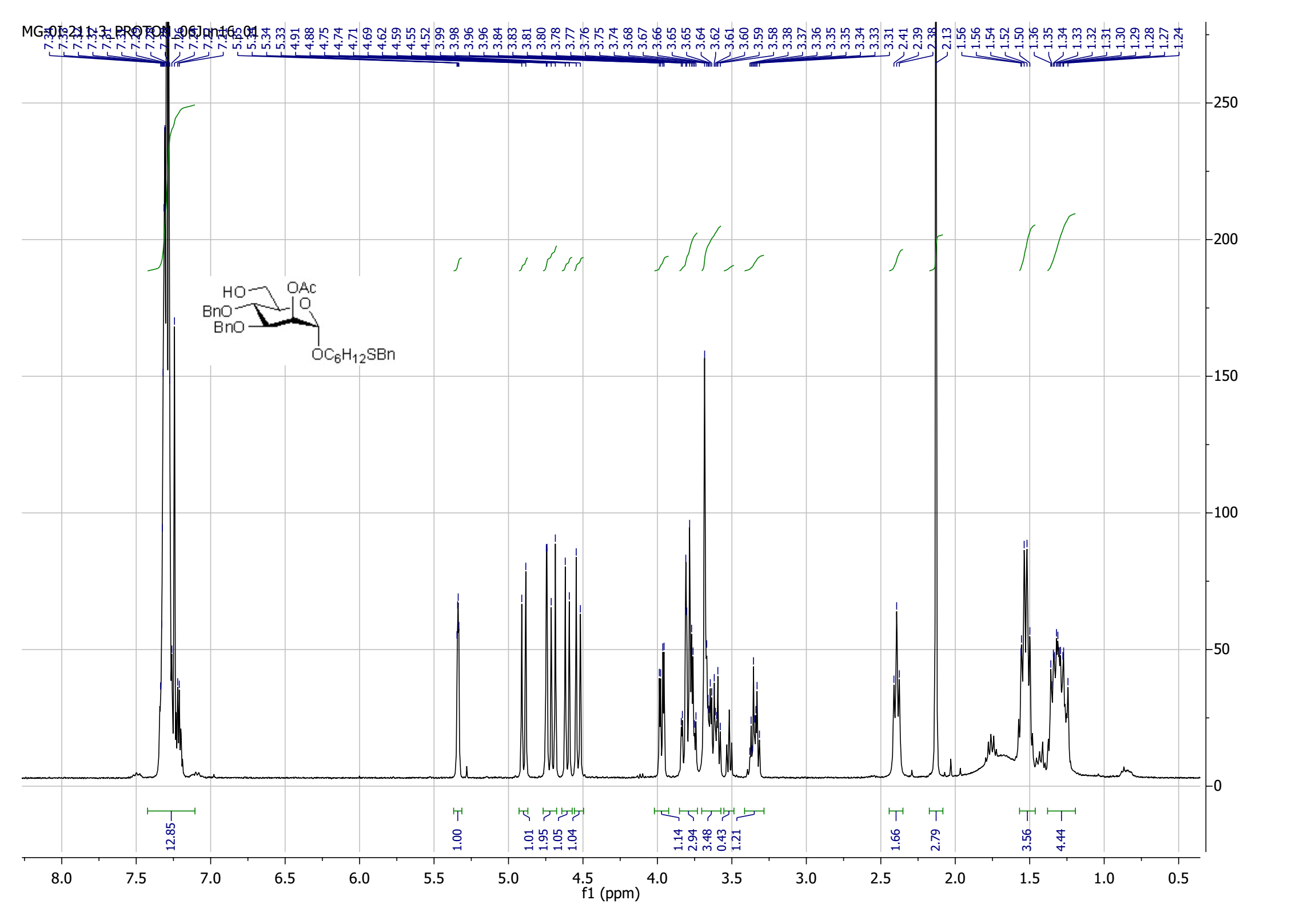


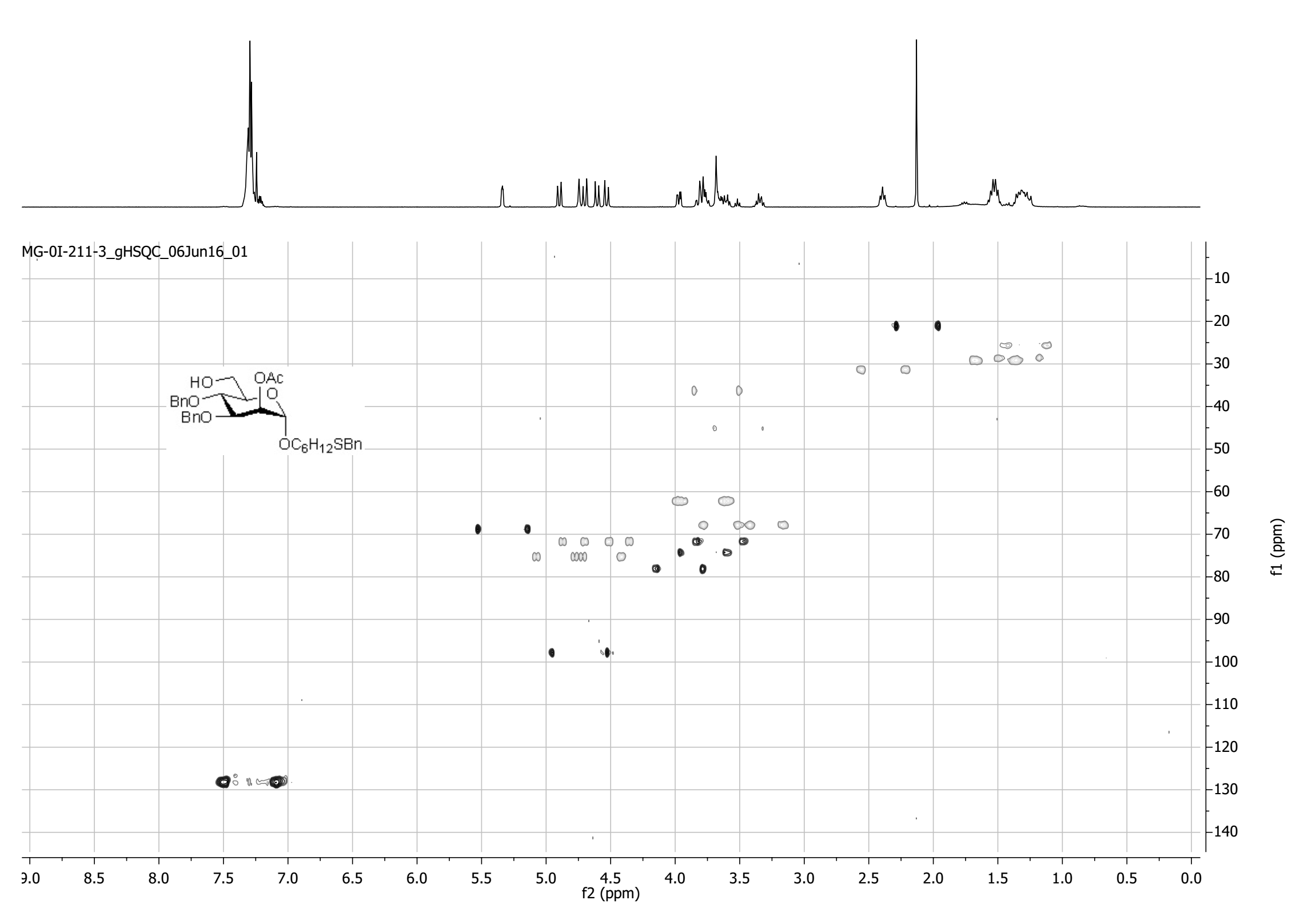


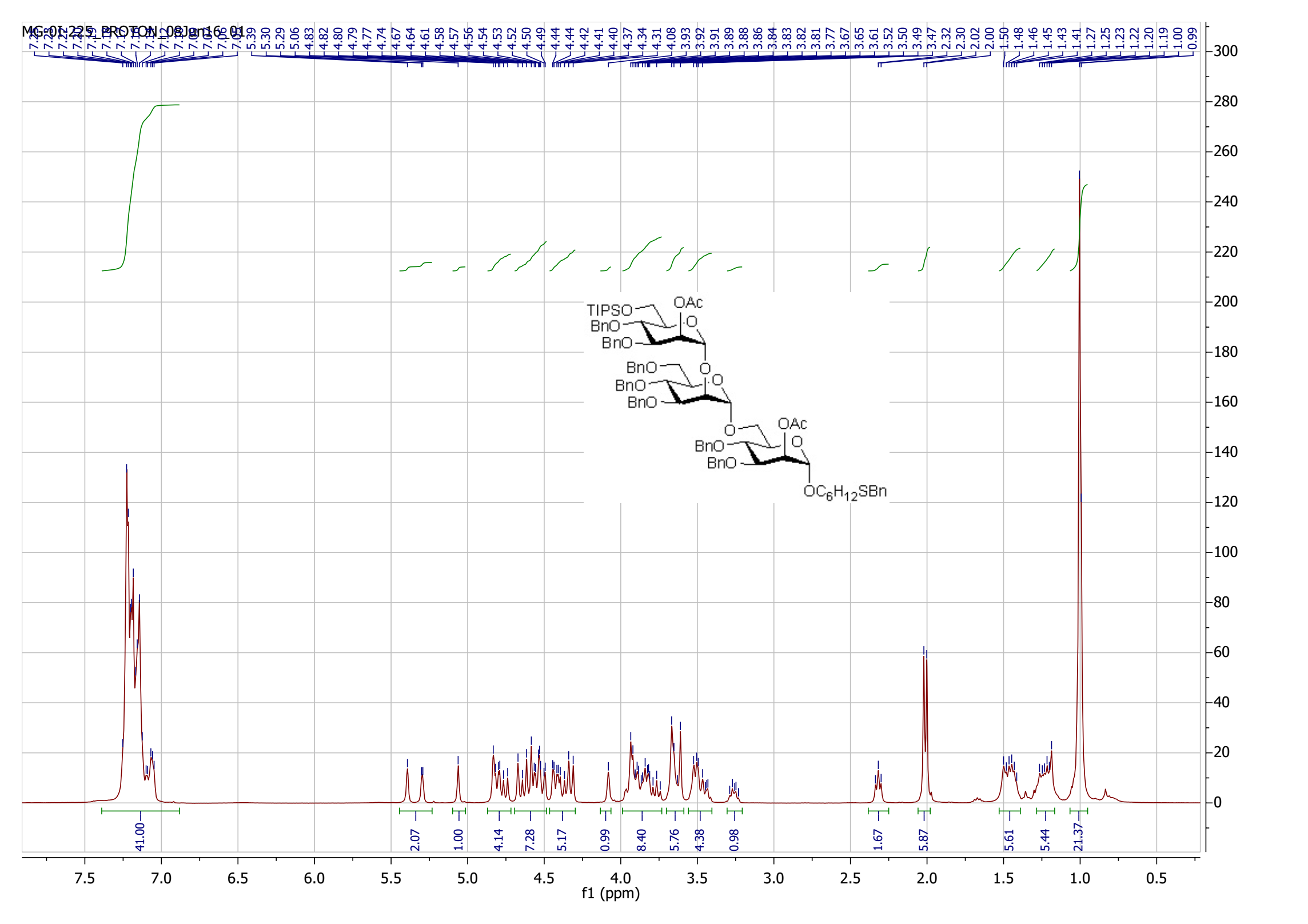


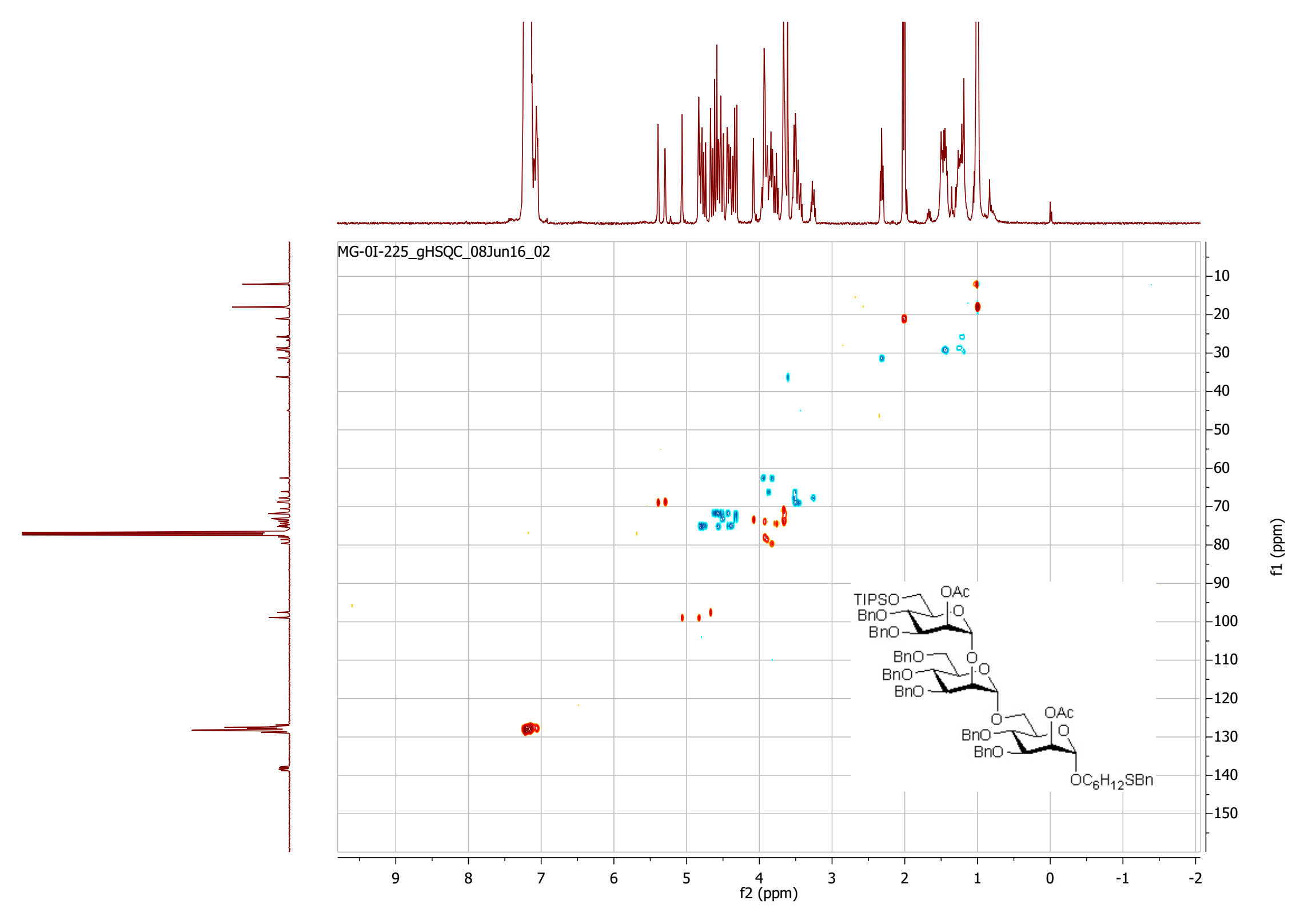


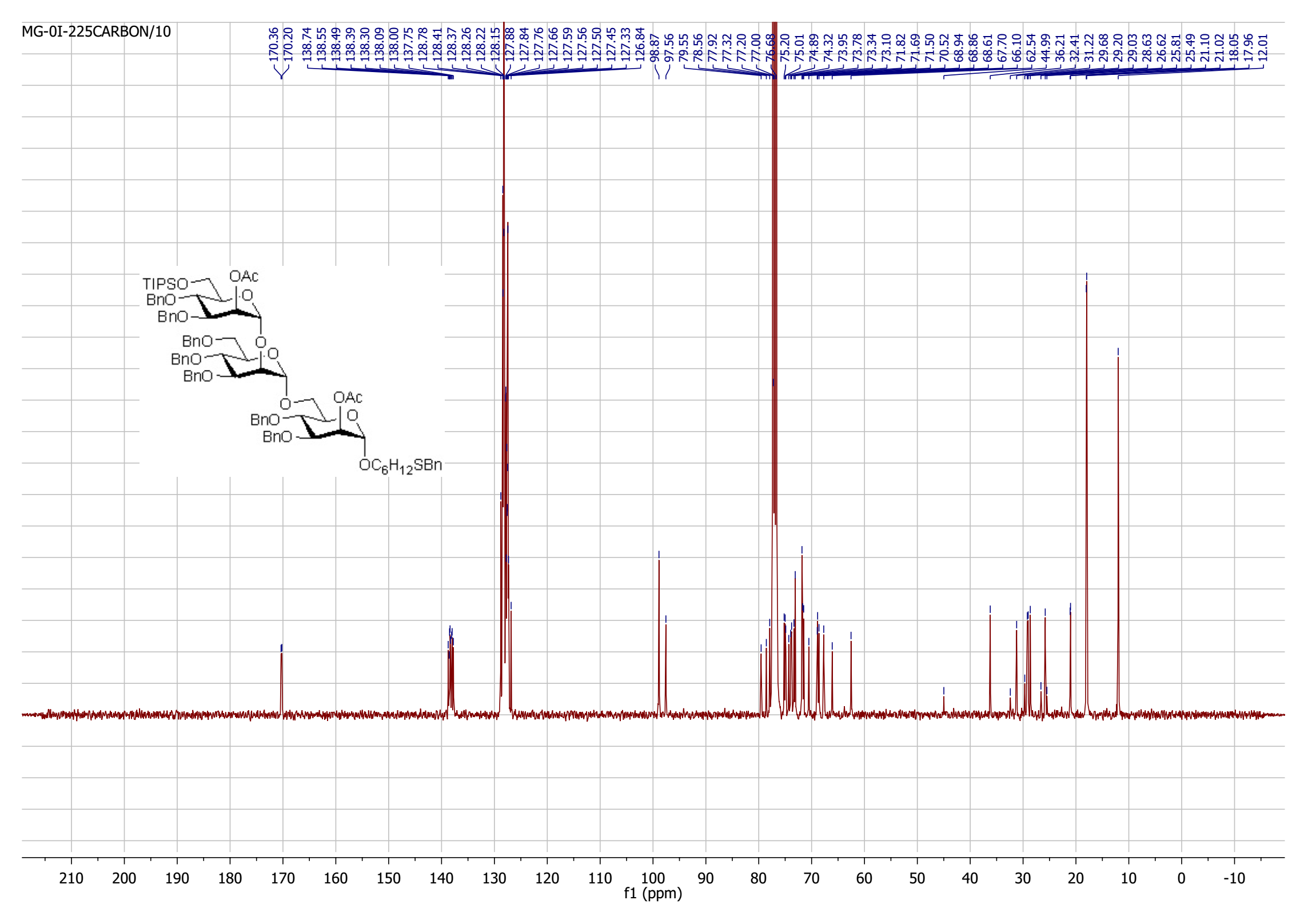


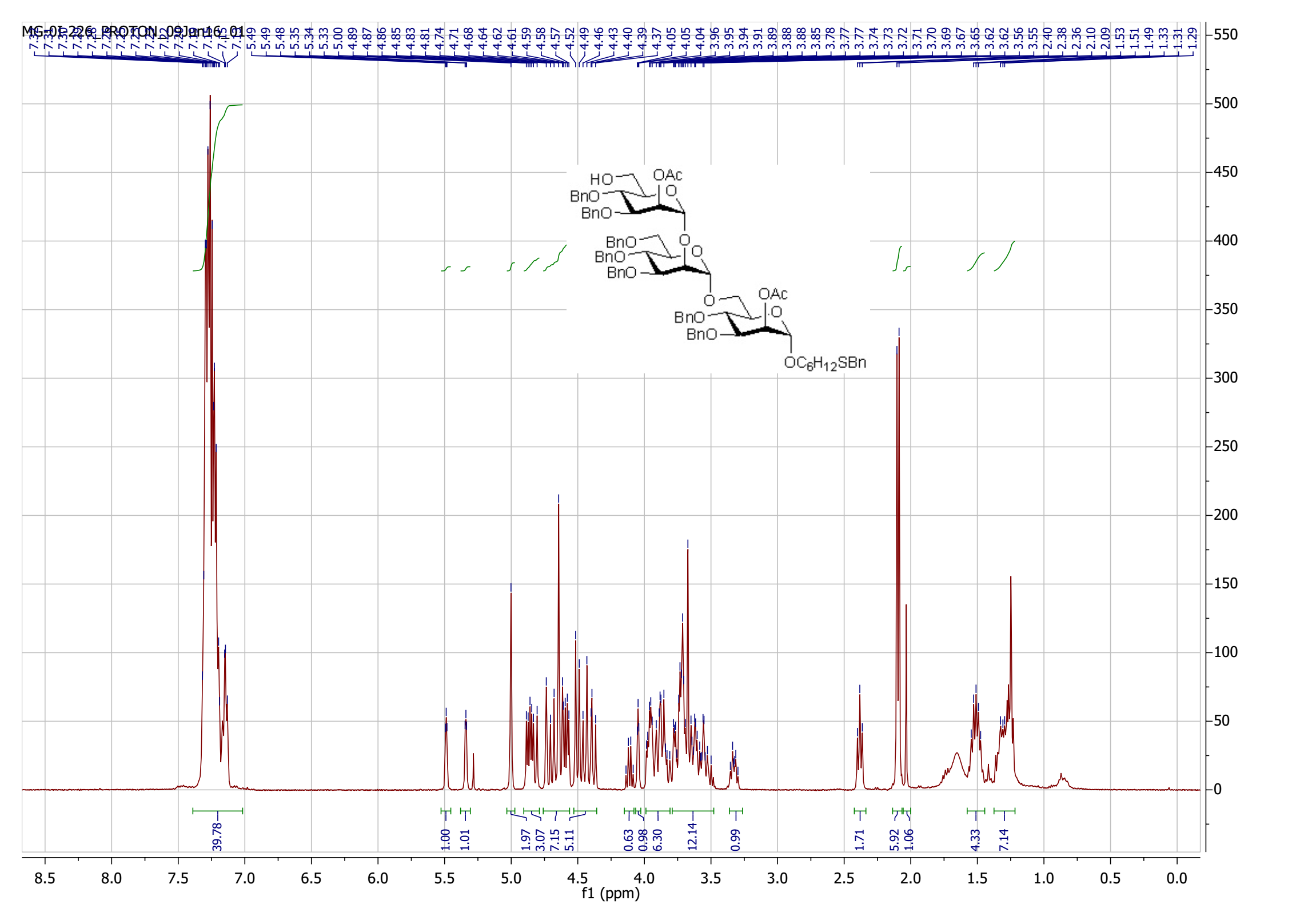


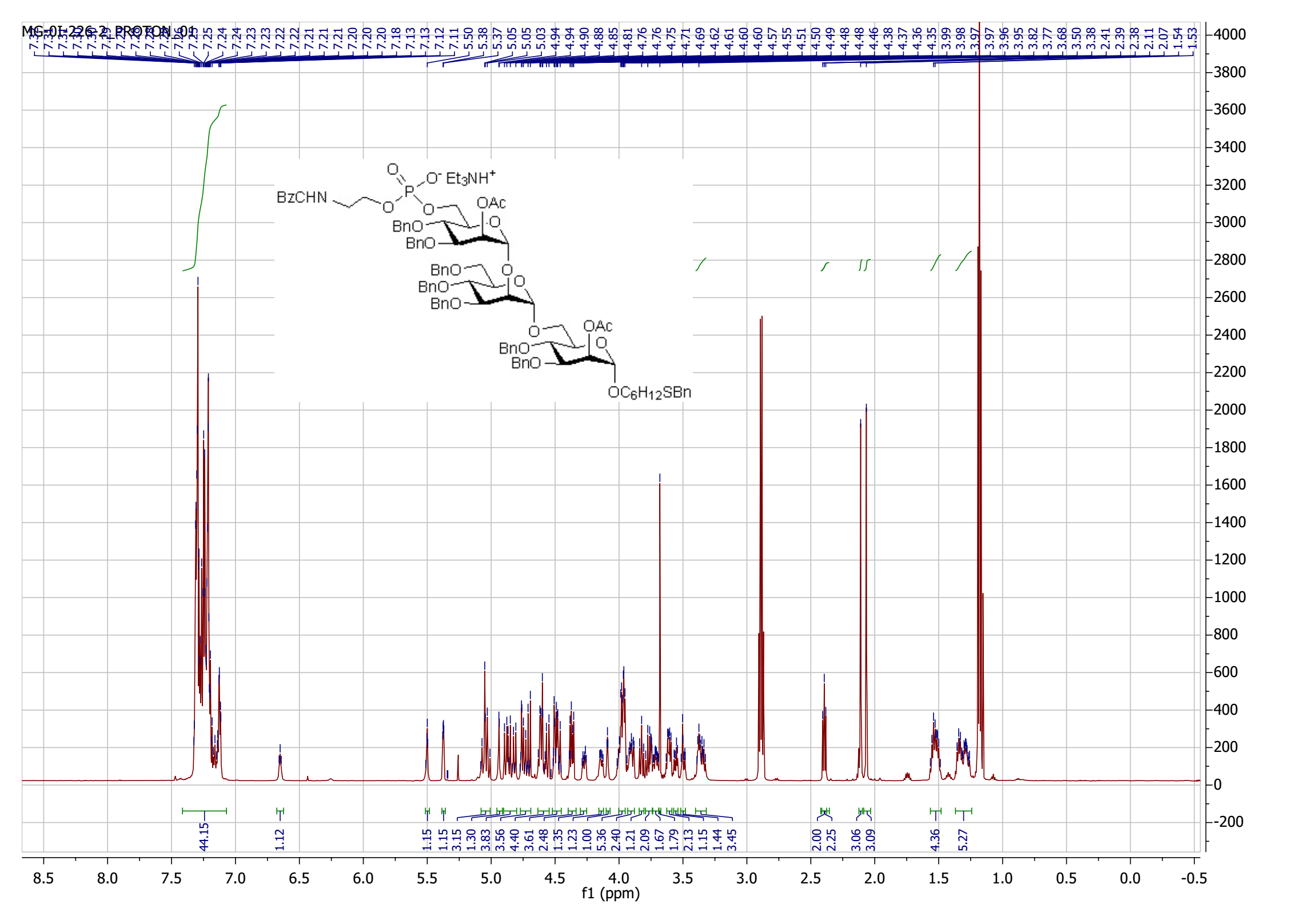


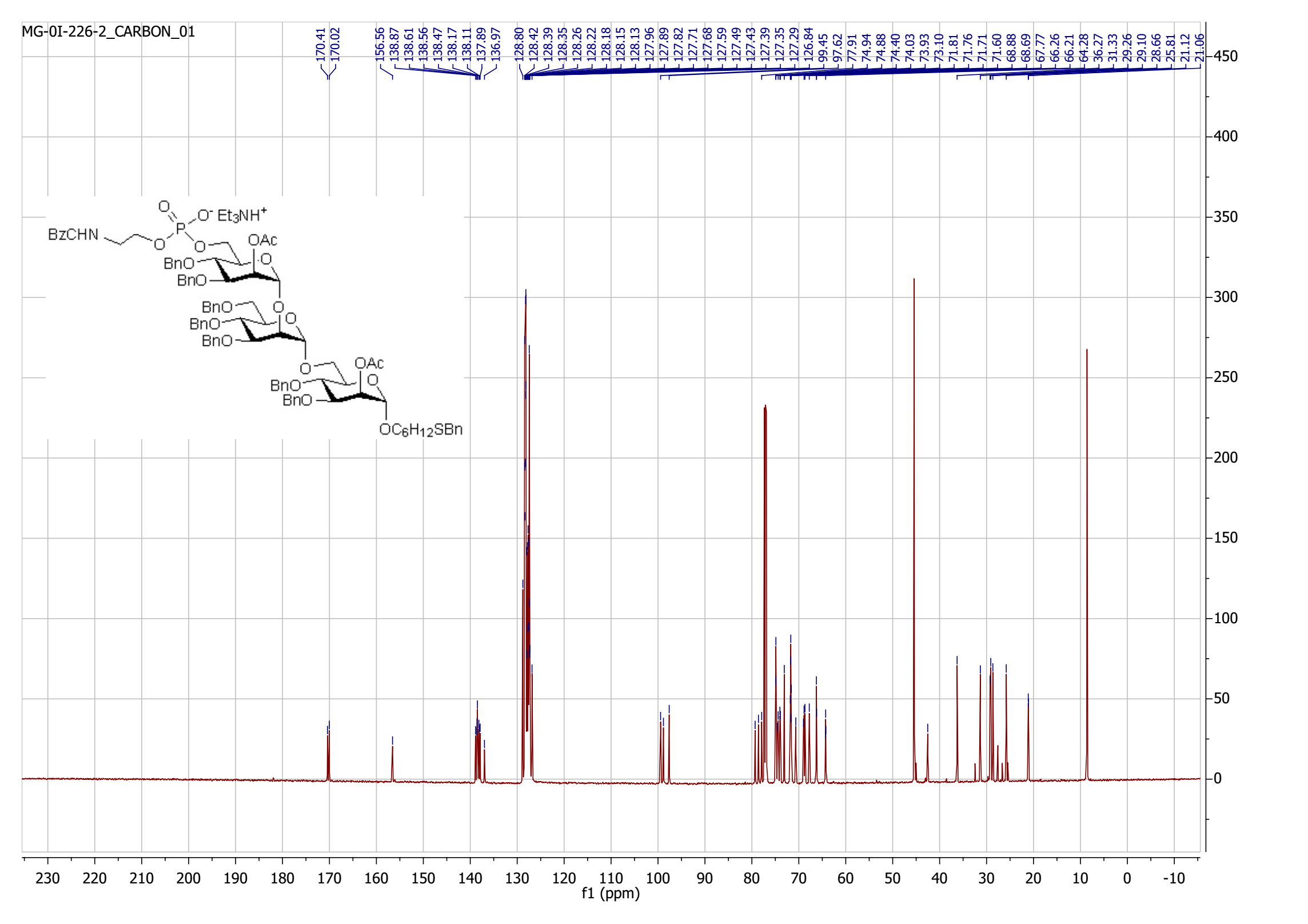


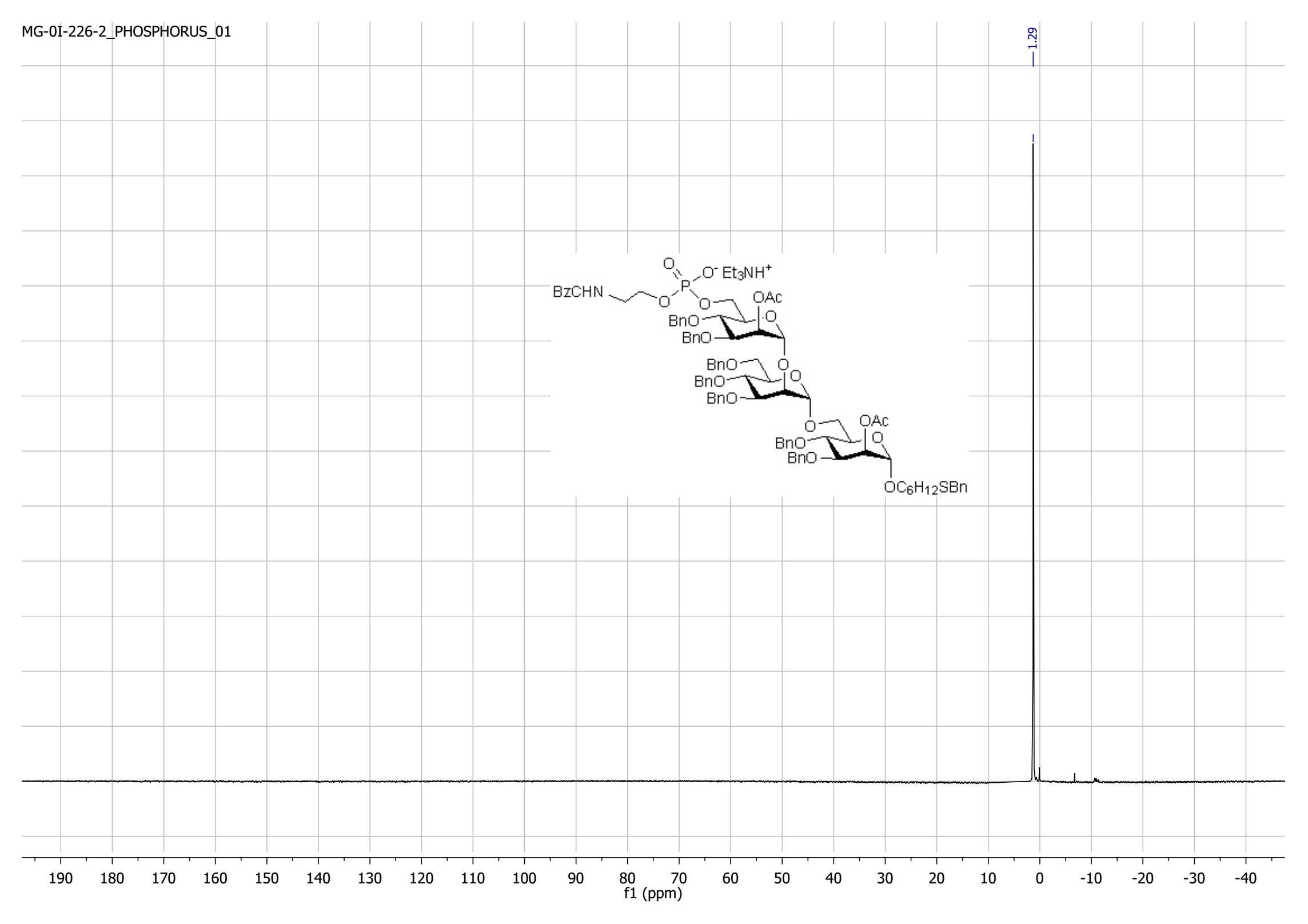


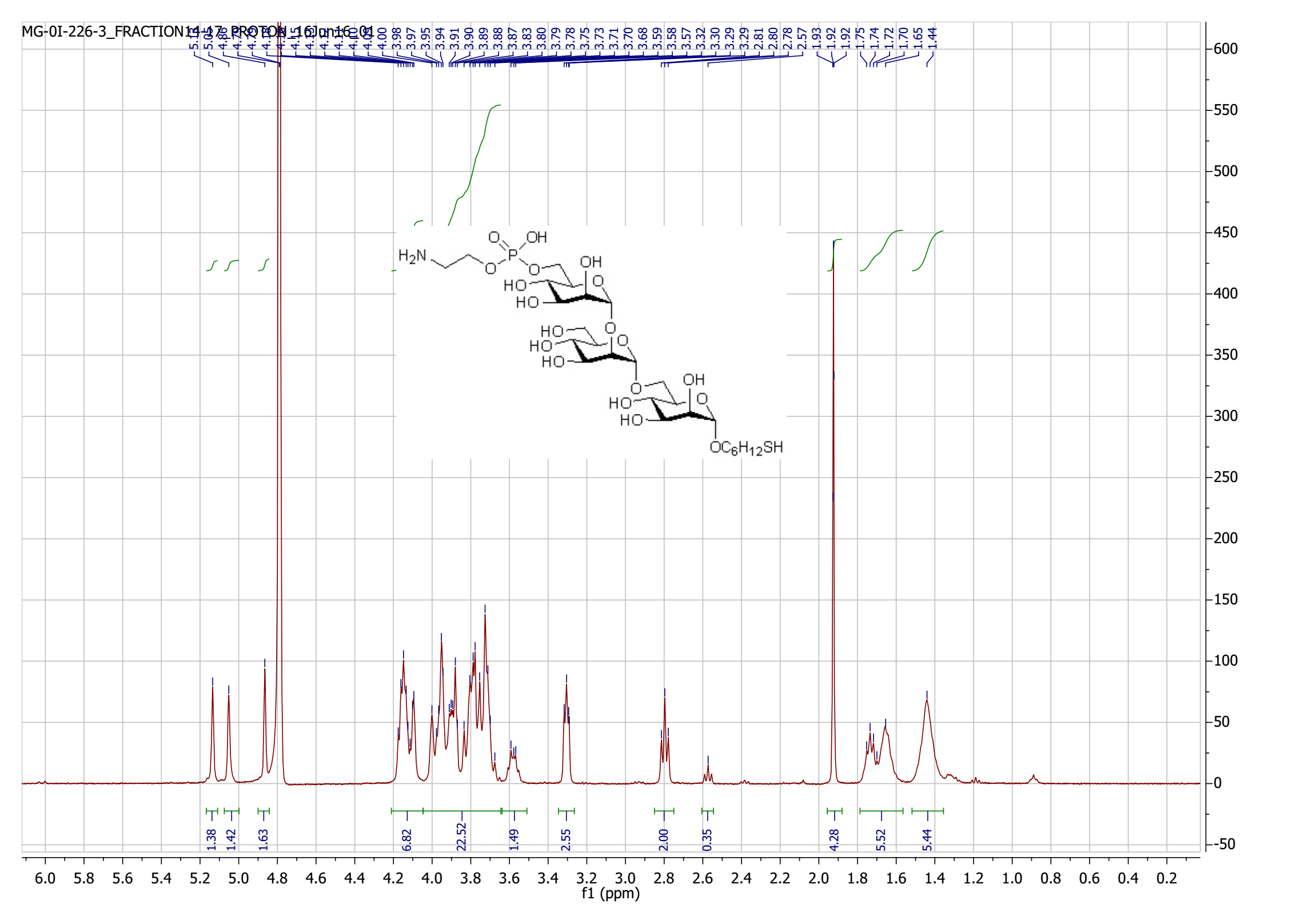


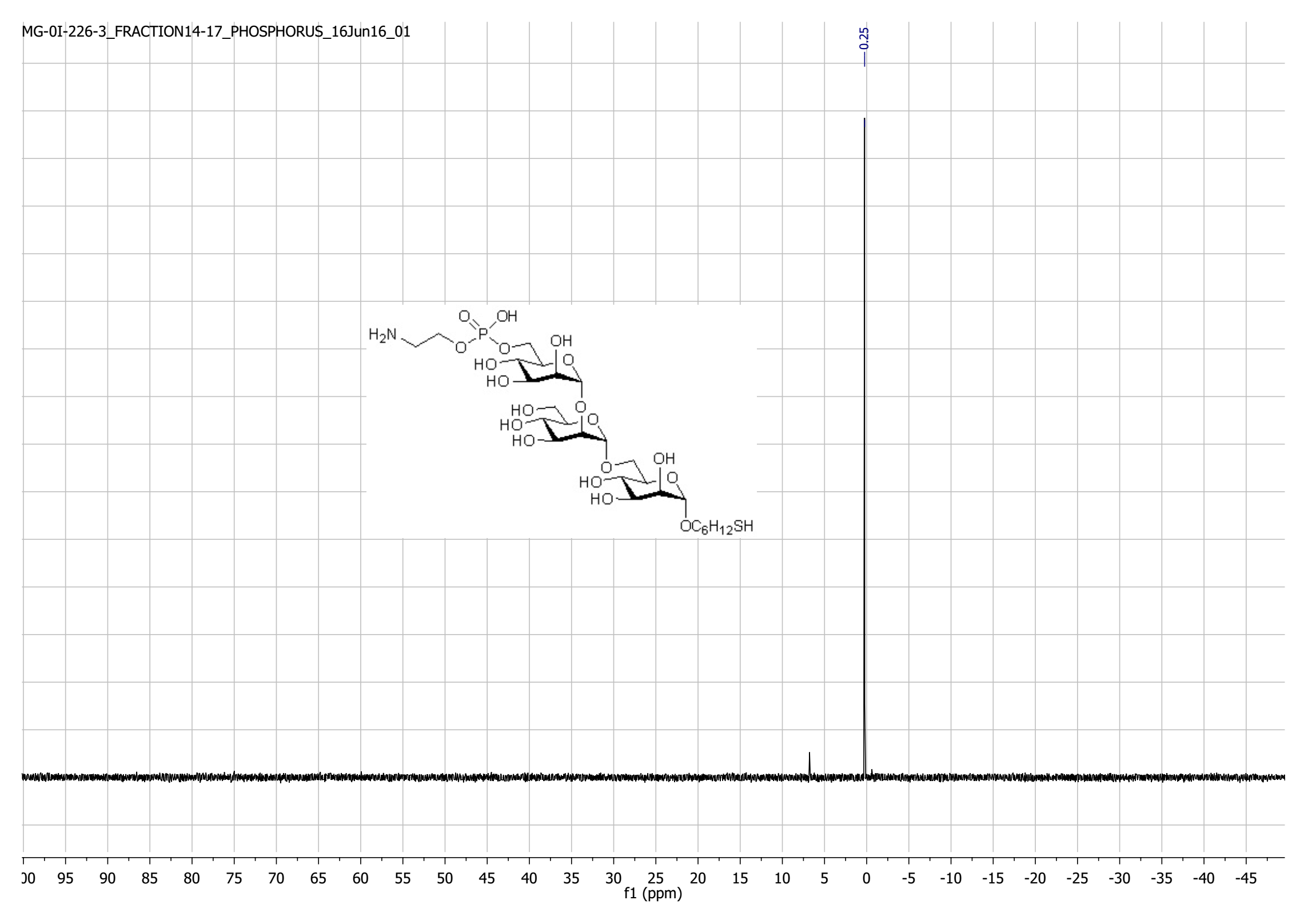


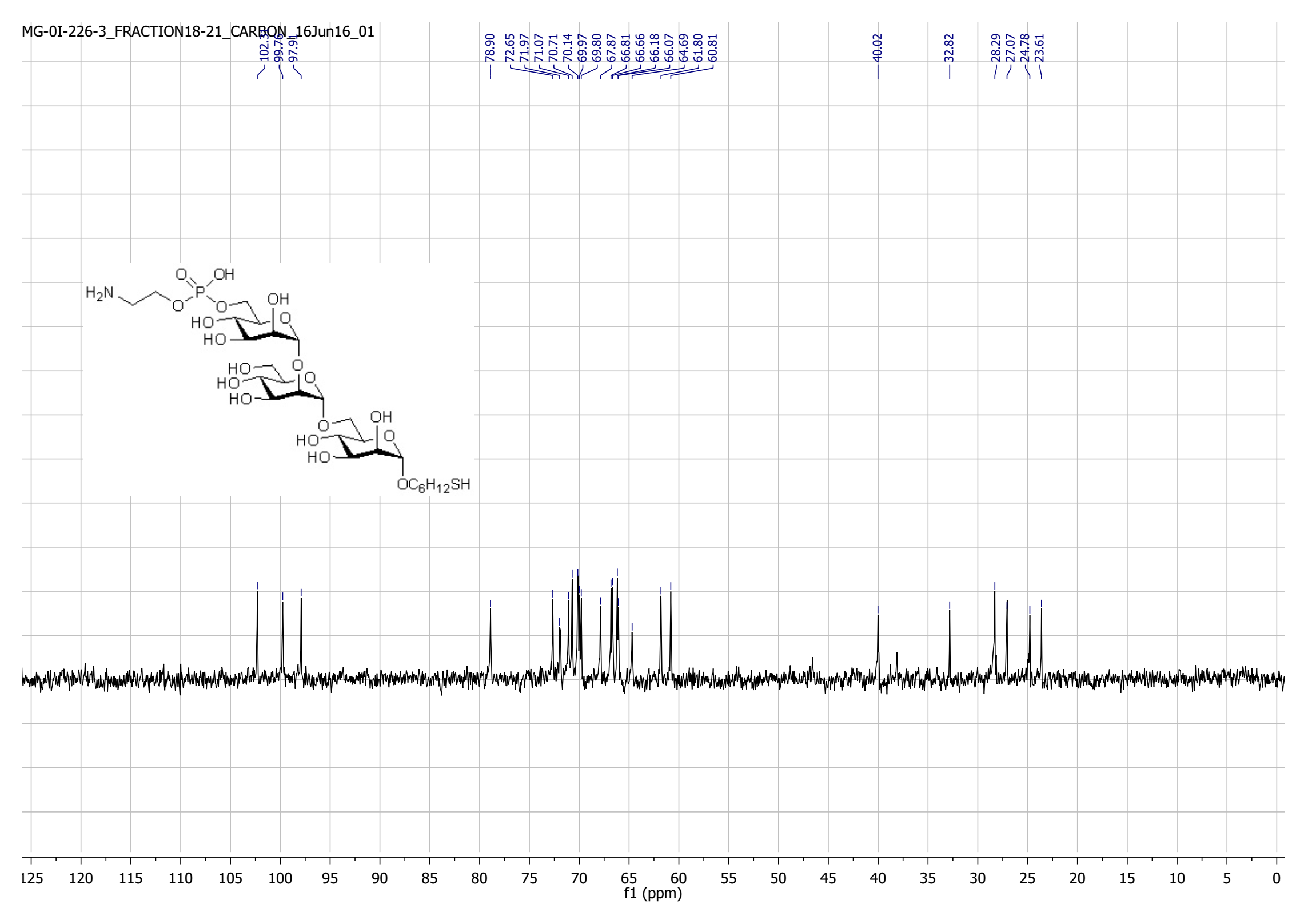


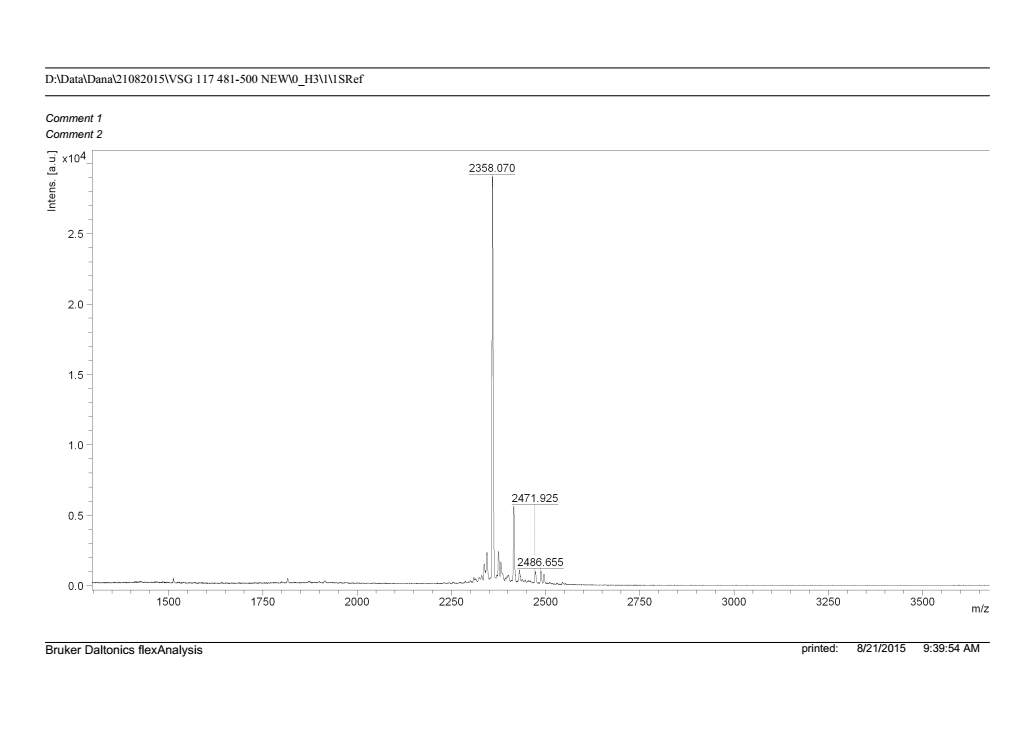


**References**

1. Grube Maurice, Lee Bo‐Young, Garg Monika, Michel Dana, Vilotijević Ivan, Malik Ankita, et al. Synthesis of Galactosylated Glycosylphosphatidylinositol Derivatives from Trypanosoma brucei. Chemistry – A European Journal. 2018;24: 3271–3282. doi:10.1002/chem.201705511

2. Tsai Y-H, Götze S, Vilotijevic I, Grube M, Silva DV, Seeberger PH. A general and convergent synthesis of diverse glycosylphosphatidylinositol glycolipids. Chem Sci. 2012;4: 468–481. doi:10.1039/C2SC21515B
