# Supplementary figures and images for: Detection of antibodies against the African parasite Trypanosoma brucei using synthetic glycosylphosphatidylinositol oligosaccharide fragments
Source: Glycoconj J. 2025 Jun 24;42(3-4):147–58. doi: 10.1007/s10719-025-10186-x (PMC12350571; doi:10.1007/s10719-025-10186-x)

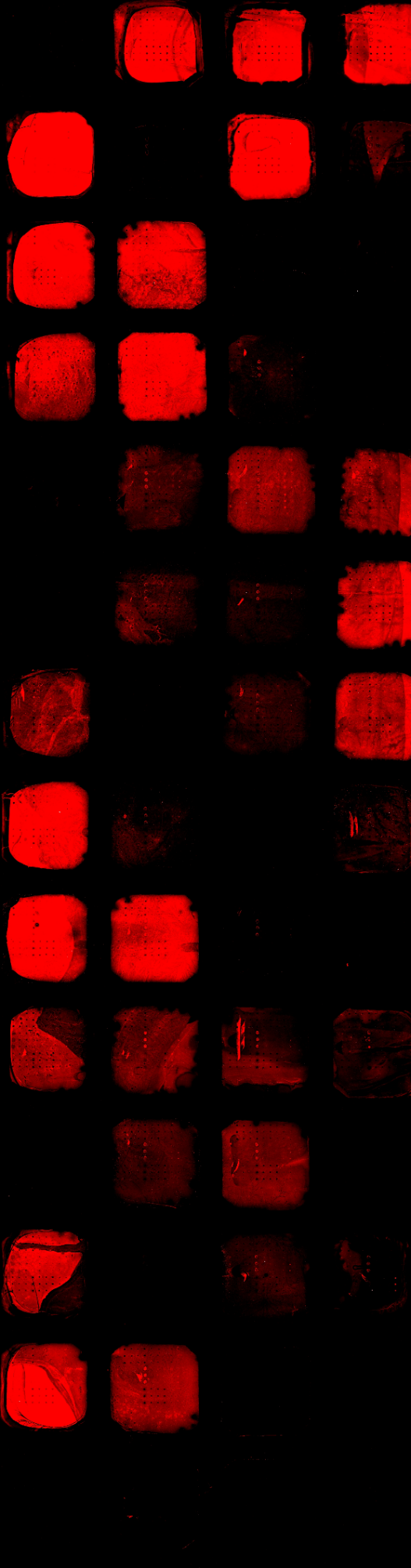

Supplement: Supplementary file 2 — Supplementary Material 2 [file 10719_2025_10186_MOESM2_ESM.png]

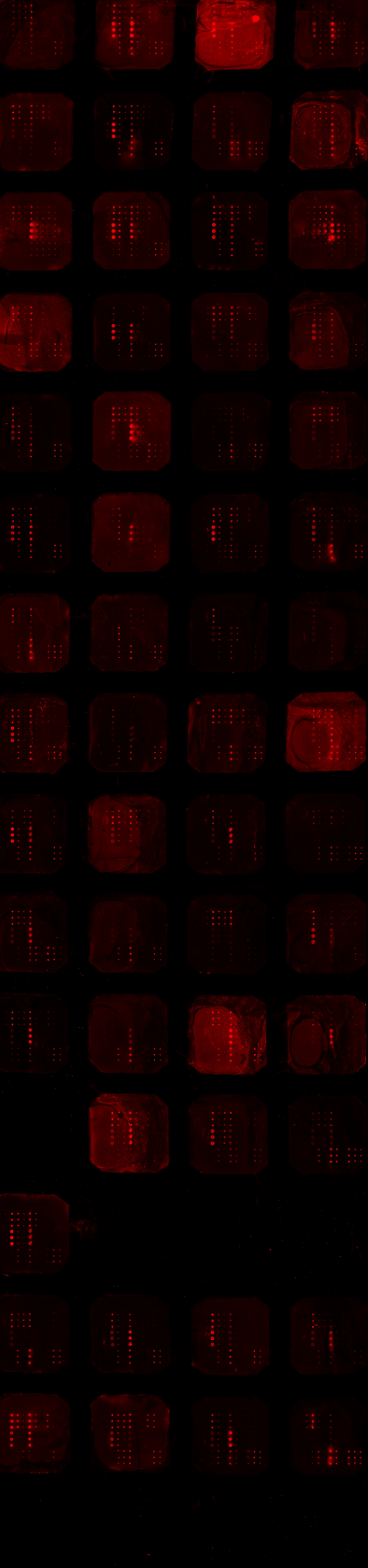

Supplement: Supplementary file 3 — Supplementary Material 3 [file 10719_2025_10186_MOESM3_ESM.png]

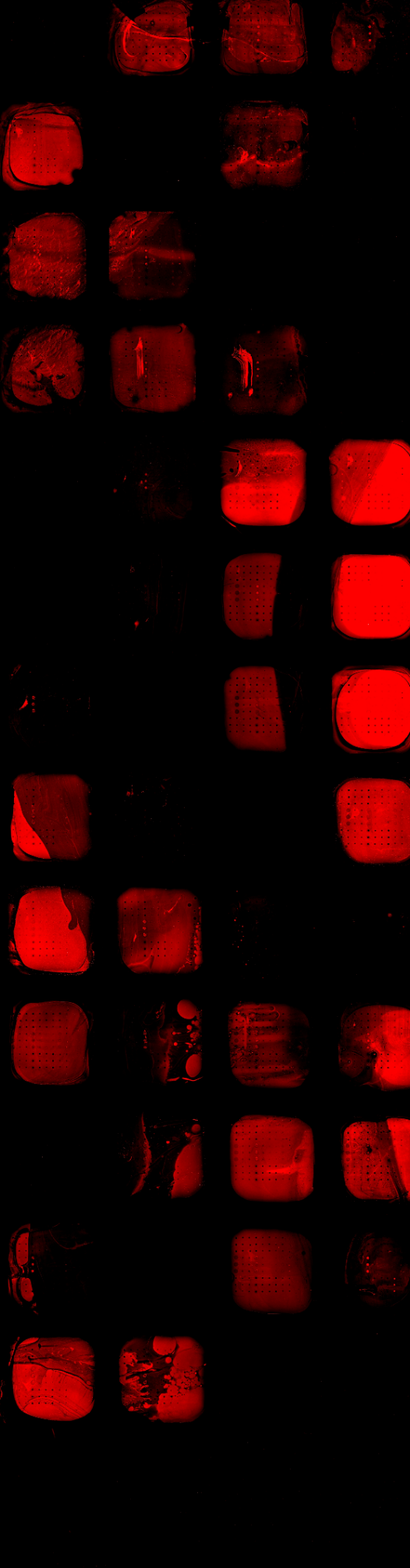

Supplement: Supplementary file 4 — Supplementary Material 4 [file 10719_2025_10186_MOESM4_ESM.png]

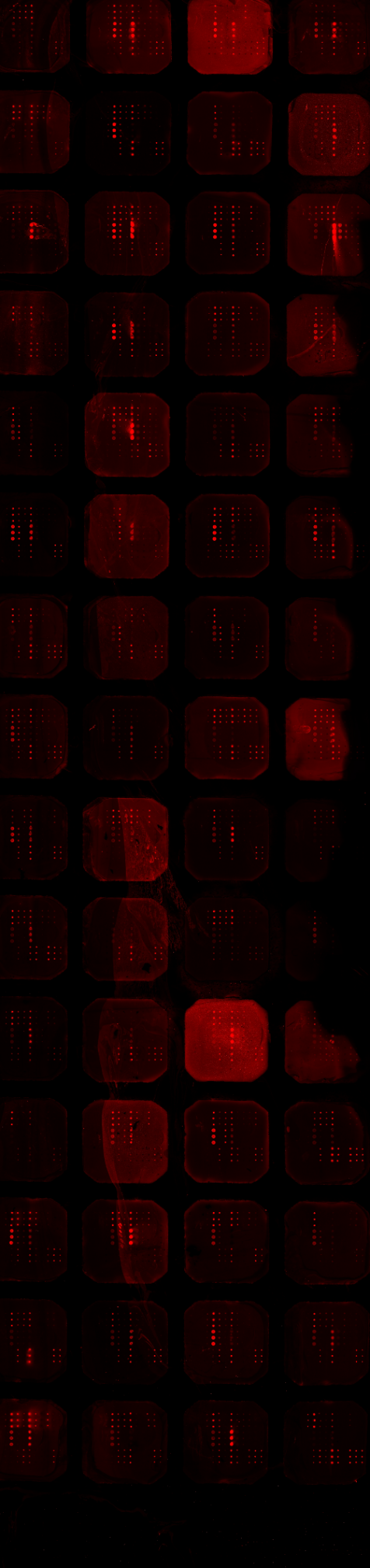

Supplement: Supplementary file 5 — Supplementary Material 5 [file 10719_2025_10186_MOESM5_ESM.png]

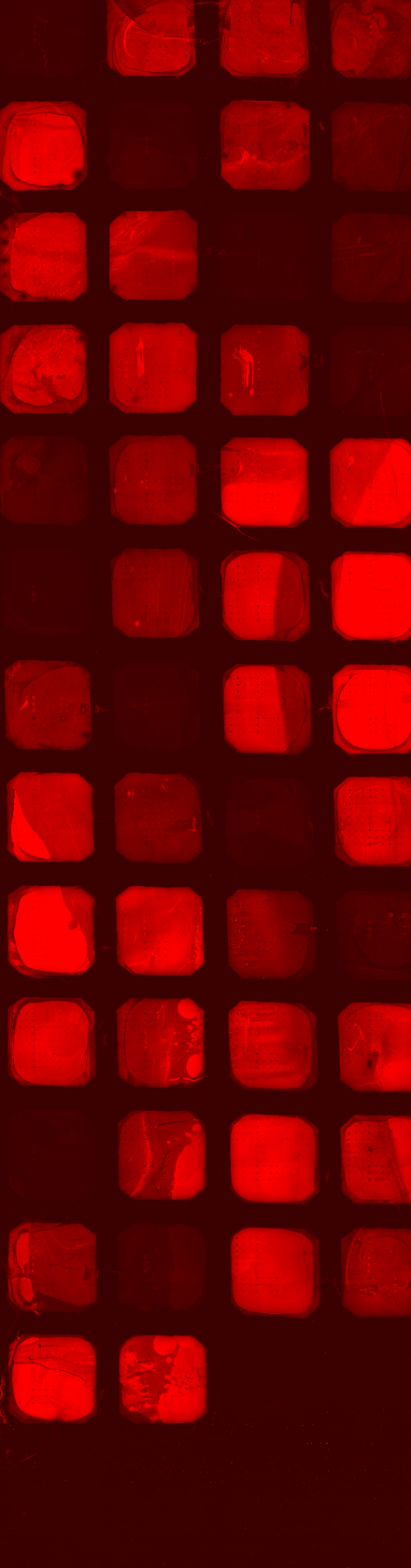

Supplement: Supplementary file 6 — Supplementary Material 6 [file 10719_2025_10186_MOESM6_ESM.png]

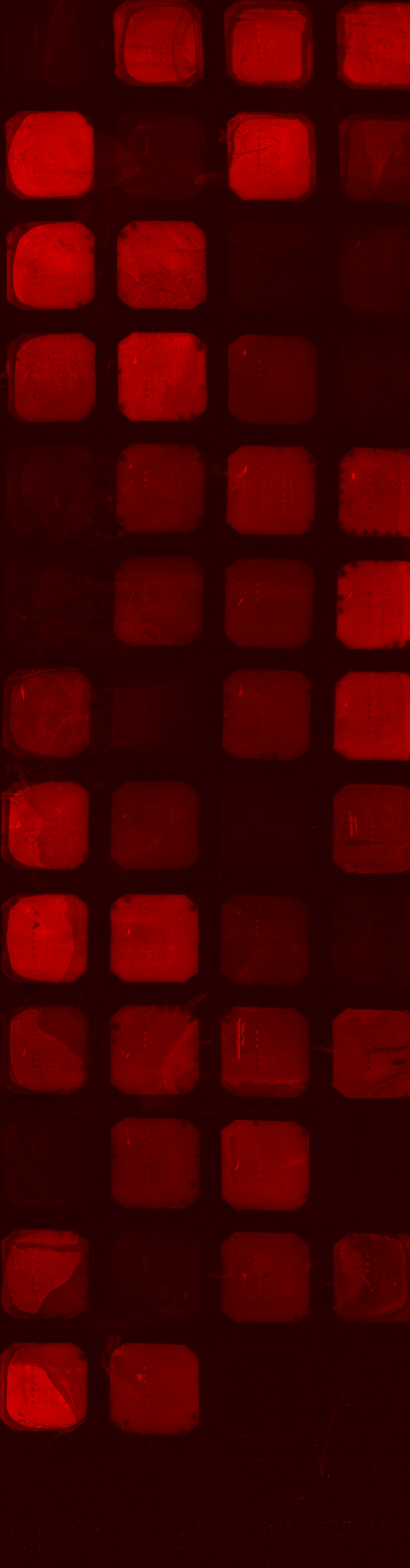

Supplement: Supplementary file 7 — Supplementary Material 7 [file 10719_2025_10186_MOESM7_ESM.png]

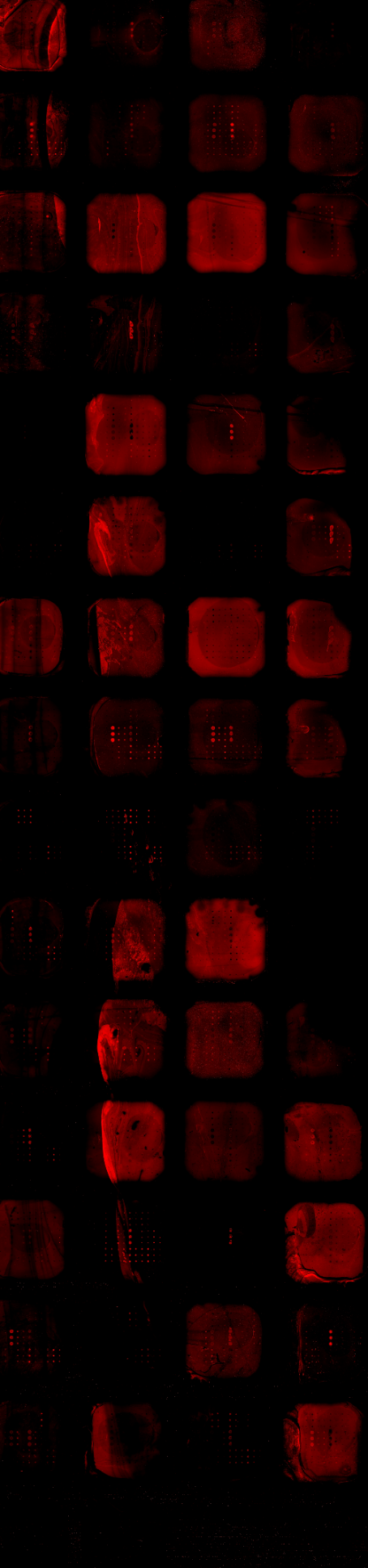

Supplement: Supplementary file 8 — Supplementary Material 8 [file 10719_2025_10186_MOESM8_ESM.png]

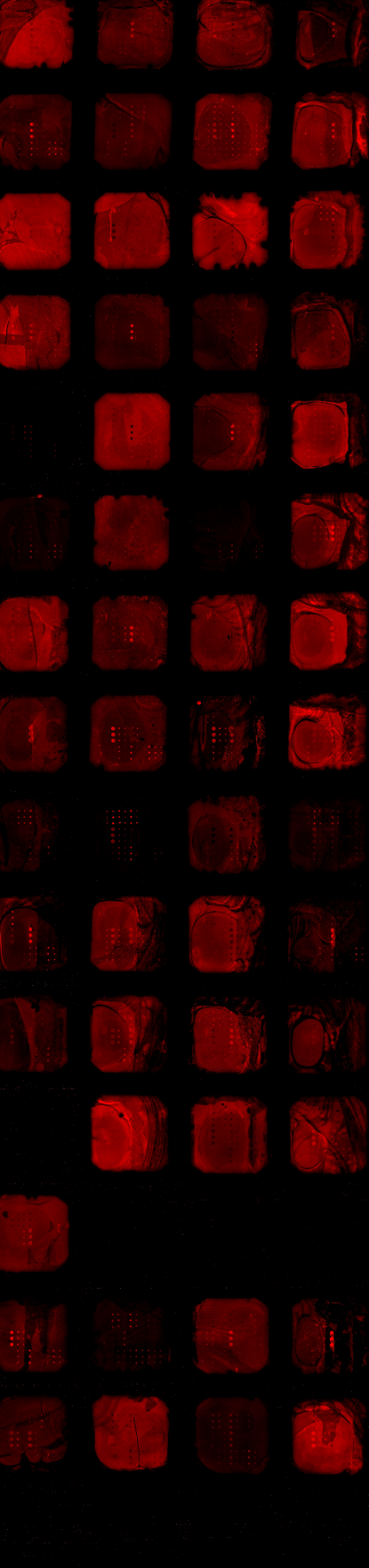

Supplement: Supplementary file 9 — Supplementary Material 9 [file 10719_2025_10186_MOESM9_ESM.png]
